# Past and future trends of Egypt’s water consumption and its sources

**DOI:** 10.1038/s41467-021-24747-9

**Published:** 2021-07-23

**Authors:** Catherine A. Nikiel, Elfatih A. B. Eltahir

**Affiliations:** Department of Civil and Environmental Engineering, Ralph M. Parsons Laboratory, Massachusetts Institute of Technology, Cambridge, MA USA

**Keywords:** Socioeconomic scenarios, Sustainability, Hydrology

## Abstract

For millennia the Nile supplied Egypt with more water than needed. As the population grew and the economy expanded, demand on water increased accordingly. Here, we present a comprehensive analysis to reconstruct how total demand on water outstripped supply of the Nile water in the late 1970s, starting from a surplus of about 20 km^3^ per year in the 1960s leading to a deficit of about 40 km^3^ per year by the late 2010s. The gap is satisfied by import of virtual water. The role of economic growth in driving per capita demand on water is quantified based on detailed analysis of water use by agriculture and other sectors. We develop and test an empirical model of water demand in Egypt that relates demand on water to growth rates in the economy and population. Looking forward, we project that within this decade of the 2020 s, under nominal scenarios of population and economic growth, Egypt is likely to import more virtual water than the water supplied by the Nile, bringing into question the historical characterization of Egypt as “the gift of the Nile”.

## Abstract (web summary)

A historical reconstruction of water use in Egypt shows the change in relative use of Nile water versus virtual water import, especially in the highly consumptive agriculture sector. A range of future projections of water demand are offered based on several plausible socioeconomic scenarios.

## Introduction

“The Egypt to which the Hellenes come in ships is a land which has been won by the Egyptians as an addition, and that it is a gift of the [Nile].” (Herodotus Book 2:5)^1^.

Following expansion of the Sahara Desert, thousands of years ago, and migration of native populations to shelter in the Nile Valley, an intimate relationship developed between an emerging Egypt and the Nile. This connection has manifested in historical, political, ecological, and hydrological dimensions. However, Egypt’s fast-growing population and developing economy have strained already scarce water resources through dietary changes and municipal and industrial consumption. Egypt is facing external pressures on perceived water rights, limited national water resource availability, and a struggle to fashion a sustainable development vision for its future. The current policies regarding irrigation in the New Lands, the current rate of water reuse, and the level of success achieved in reducing fertility rates will not be enough to close the demand gap in the future^2^.

**Fig. 1:**
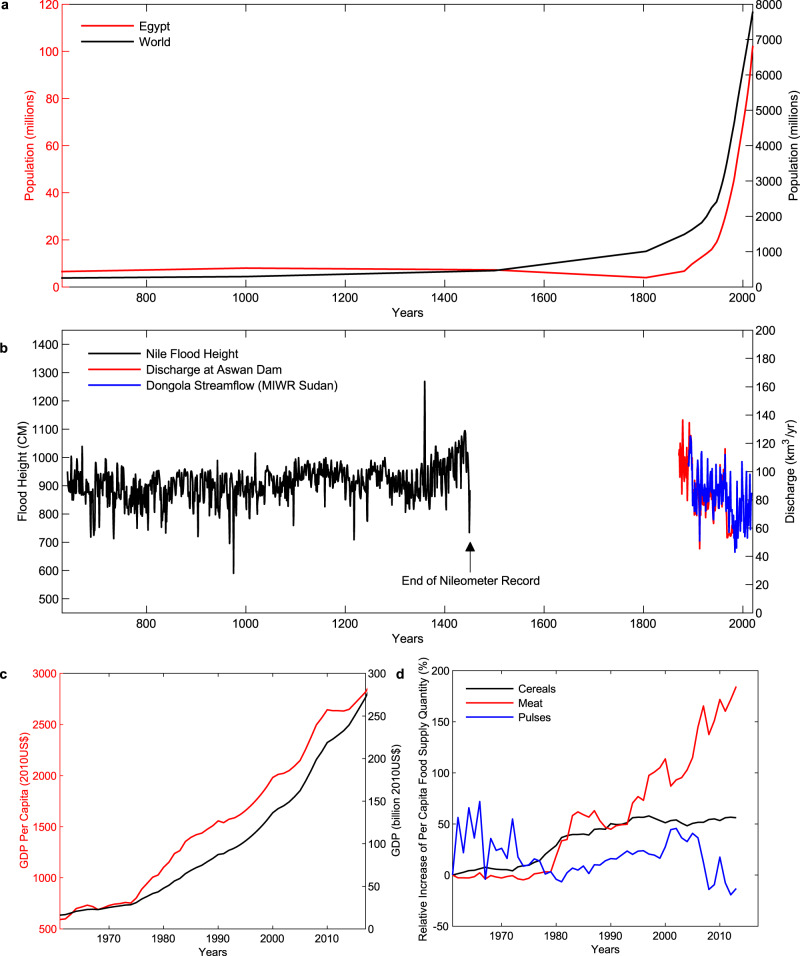
Context for water challenges in Egypt. **a** Egyptian population [red line] and world population [black line] from 1 to 2020 C.E^3^. (Supplementary Table 1.4). **b** Qualitative comparison of stable Nile Flood heights from 641 to 1451 [black line – left axis]^13^ and decreasing modern yearly average discharge from Aswan Dam [red line – right axis] and flow at Dongola [blue line – right axis]^16, 17^ (Supplementary Tables 1.14 and 1.20). We note that the reader should not interpret a 1:1 correlation between the y-axes, and that the two separate groupings of data have been presented together to reflect that there has been little change in the Nile based on geologic survey^15^. **c** Gross Domestic Product (Constant 2010 $US) [black line] and GDP per Capita [red line] in Egypt^6^ (Supplementary Table 1.6). **d** Relative change in per capita food supply (kg/capita/year) relative to 1961 for meat [red line], cereals [black line], and pulses (beans, chickpeas, lentils) [blue line]. 1961 values for meat, cereal, and pulses are 10.71, 161.43, and 6.5 kg/capita/yr respectively^7^ (Supplementary Table 1.10).

Egypt’s population has been growing rapidly in recent decades, at a rate of 2.1% annually from 1989 to 2018^3^, following a similar trajectory of world population growth (Fig. 1a). This added population places pressure on limited water resources, both through direct consumption and through increased demand for food and other products. In 2017, the total renewable water resource per capita was 628 m^3^/yr already below the level for water scarcity according to the Falkenmark Index^4,5^. This pressure due to population growth, while straightforward, is essential to include while drawing the picture of historical and future demand for water, as Egypt faces increasing scarcity of natural resources.

Egypt has also experienced rapid economic growth since the mid 1950’s (Fig. 1c). From 1989 to 2018, the Egyptian GDP grew at 4.4% annually, while GDP per capita grew at 2.3% annually over the same period^6^. This growth has been concurrent with an increase in water consumption for both municipal and agricultural purposes and an increase in both domestic production and imports of agricultural commodities. As GDP has grown, the diet of Egyptians has changed dramatically; increasing trade connections with other food producers have increased availability of some goods, particularly animal products (Fig. 1d). The average per capita supply of proteins from animal origins increased by 39% from 18 g/cap/day in 1999–2001 to 25 g/cap/day in 2011–2013^7^. At the same time, the prevalence of undernourishment dropped by 0.7 to 4.5%^7^. Egypt currently ranks among the top countries globally in the daily per capita caloric supply (3522 kcal/capita/day), just behind the United States^8^. These trends are projected to continue, especially in meat consumption which has a strong relationship with GDP per capita^9^. The projected consumption of red meat in 2030 is 1,581,000 tonnes up from 2001 to 2017 average of 1,136,050 tonnes; the projected consumption of white meat is 1,681,000 tonnes up from 2011 to 2017 average of 1,054,740 tonnes^10^. However, calories coming from meat are still a small percentage of overall daily caloric intake, increasing from 2.4% to 3.5% from 1961 to 2013^11^.

At the same time, water supply from the Nile, which accounts for 98% of renewable water resources in Egypt, has remained relatively steady (Fig. 1b)^12^. While detailed flow records do not exist prior to the installation of modern gauging systems (circa mid to late 1800’s), the Nile’s floods have been monitored and recorded for millennia. Records of flood heights from the Rhoda Nileometer show these levels remained relatively constant over the 800-year record^13,14^. Geologic research has suggested “very little downcutting [in the riverbed has occurred] in Nubia since [the time of the New Kingdom some 3000 years ago]” suggesting that flood heights across the full 700-year record are directly comparable^15^. In the modern record, flows recorded at Aswan and Dongola have been slightly decreasing, as a result of increased withdrawal of natural flows upstream from Sudan’s withdrawal of 4 km^3^ in 1959 to current withdrawals of 13–16.7 km^3^ (refs. ^16–21^).

In this work we identify and quantify actions that Egypt has taken over the past six decades to manage internal pressures on water resources. A detailed, long term picture of the changes in water demand and water use is constructed and used as a foundation to project demand on water in the near future, and further to propose solutions that can be explored towards more efficient water use. While much past work^22–25^, including governmental literature, has presented snapshots in time of water use and virtual water trade in Egypt, we use water and crop data to quantitatively describe in significant detail water use in Egypt, over a period of six decades. The key innovations of our study are in the detailed year-by-year reconstruction of trends in water use down to the individual crop level, the improved understanding of the factors that drive these trends, and the use of this context to project water demand into the near future based on empirical demand relationships. The detailed diagnosis of water use in Egypt facilitates identification of opportunities for water saving, water reuse, and improved water use efficiency in general.

## Results

### Water in Egypt: historical and future

**Fig. 2:**
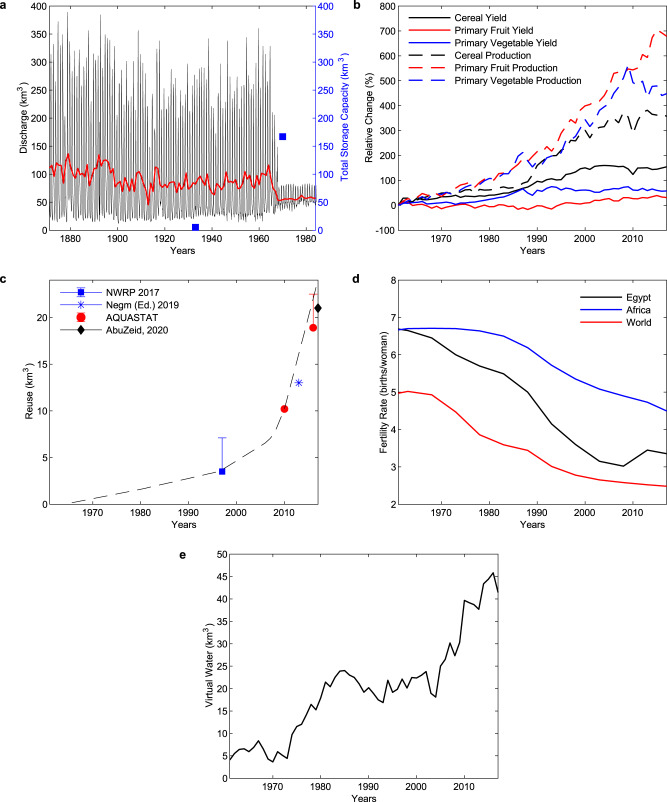
Egypt’s response. **a** Flow of the Nile at Aswan in monthly [black line] and yearly [red line] averages^16^ (Supplementary Table 1.20). Blue squares represent the total storage capacity and marks the construction of the Aswan and High Aswan Dams^12^ (Supplementary Table 1.19). **b** production (harvested tonnes) [dotted lines] and yield (production/area) [solid lines] of cereal [black], primary fruit [red], and primary vegetable [blue] crop groupings. 1961 production values are 5.0, 1.9, and 2.8 million tonnes respectively, and 1961 yield values are 29,057, 169,113, and 152,611 hg/ha respectively (Supplementary Table 1.7). **c** Estimated water available for reuse from direct agricultural drainage reuse, Nile Delta and Valley groundwater, and treated wastewater reuse. The error bars shown account for estimates of wastewater reuse in the absence of data for those years^22, 23, 25, 51^. Sources for individual components in Supplementary Fig. 9. The black dotted line shows the reuse timeseries used in analysis. **d** Total Fertility Rate (births/woman) plotted for Egypt [black line], Africa on average [blue line], and the world on average [red line]^29^ (Supplementary Table 1.5). **e** Virtual water imports (km^3^) of agricultural goods (primary and secondary crops and animal products) scaled to Egypt use equivalent (Supplementary Tables 1.1, 1.2, 1.3, and 1.11).

This paper focuses on historical and future trends in Egyptian water management: first, in the historical period, Egypt has managed water supply and demand through five avenues (Fig. 2): improving water infrastructure and management by building the High Aswan Dam; increasing in-country agricultural production through harvested area expansion and improving crop yields; expansion of water reuse; reducing population growth rate; and increasing import of agricultural products – especially staples such as wheat and maize. We integrate extensive data sets to rigorously document this historical adaptation process.

Second, future population and economic growth will increase water demand dramatically and require Egypt to rely more heavily on virtual water imports, at a higher annual growth rate than we have seen in the past. The concept of virtual water, coined in the 1990’s by Dr. Tony Allan, was first applied to the Middle East and North Africa as a region that addressed water scarcity with importation^26^. We show through our own bottom-up reconstruction of water-use that Egypt’s demand for water passed the carrying capacity of the Nile in the late 1970’s and was importing the use equivalent of at least 40 km^3^ of virtual water in the late 2010’s. Assuming persistence of the recent socioeconomic trends, we project that Egypt will import 61.5 km^3^/year during this decade of the 2020s. At that point Egypt will be importing more virtual water from abroad than they have been withdrawing from the Nile internally on average for the past 30 years. A comprehensive analysis framework is shown in the results and methods sections for reconstruction of past water uses and projection of future water demand leading to these conclusions.

### Egypt’s response to increasing water demands

Rapid changes in demographic and economic factors in Egypt have spurred equally large responses in an attempt to control and manage water supply and demand. Egypt’s effort in the areas of controlling variability in the Nile water supply, agricultural productivity, water reuse, fertility rates, and food imports have been substantial. However, future pressures will require Egypt to intensify some of these measures and adopt new approaches.

Much of the work done by Egypt can be viewed as a transition through several water states. As we have seen in Fig. 1b, the Nile river was largely uncontrolled for most of its history. Agriculture, and Egyptian life in general was guided by the seasonal flood and drought cycles of the river and water still flowed freely into the Mediterranean Sea. This began to change in earnest when Egypt built the High Aswan Dam from 1960 to 1970; the initial filling curtailed the flow of the river, and the resulting reservoir with a total design capacity of 160 km^3^ reduced interannual variability of flow, providing a steady and controlled supply of water to farms, which could now control the application of irrigation to fields while minimizing losses to the Mediterranean (Fig. 2a)^27^. These decades also saw the advent of major use treaties. Through the 1959 Water Agreement between Sudan and Egypt, Egypt would be allocated 55.5 km^3^ of the Nile water annually while Sudan was allocated 18.5 km^3^ (ref. ^20^). When the treaty was established, Egypt was consuming 48 km^3^ according to the agreement.

In the 1980’s Egypt began to experience an agricultural boom. This was achieved partly through expansion of agricultural area, especially on the edges of the Delta (dubbed the “New Lands”), and partly through yield increases in virtually every major crop (Fig. 2b). Egypt ranks among the highest yield producers of many crops including wheat, maize, rice, and cotton^28^. While this increased water demand, a concurrent push to increase the reuse of water – from direct agricultural drainage, groundwater pumping, and wastewater reuse – made up for some of the increased consumption (Fig. 2c).

At the same time, Egypt began a push towards reducing population growth, and reduced its total fertility rate by nearly half in thirty years, down to a rate of 3 births per woman in the late 2000’s (Fig. 2d)^29^. This decrease has been much steeper than the fertility rate drops in Africa as a whole and on par with global fertility rate reductions.

Even with these adaptation measures, Egypt’s demand continued to rise at a time when it was at full utilization of available natural water resources. Consequently, the 1970’s were also the beginning of increases in agricultural imports, viewed here through the lens of their virtual water equivalent (Fig. 2e). Import rate increased dramatically after 2000, and the cereal import dependency ratio has increased from 34% to 42% from 1999–2001 to 2011–2013^30^.

### Reconstruction of historical Nile water use

**Fig. 3:**
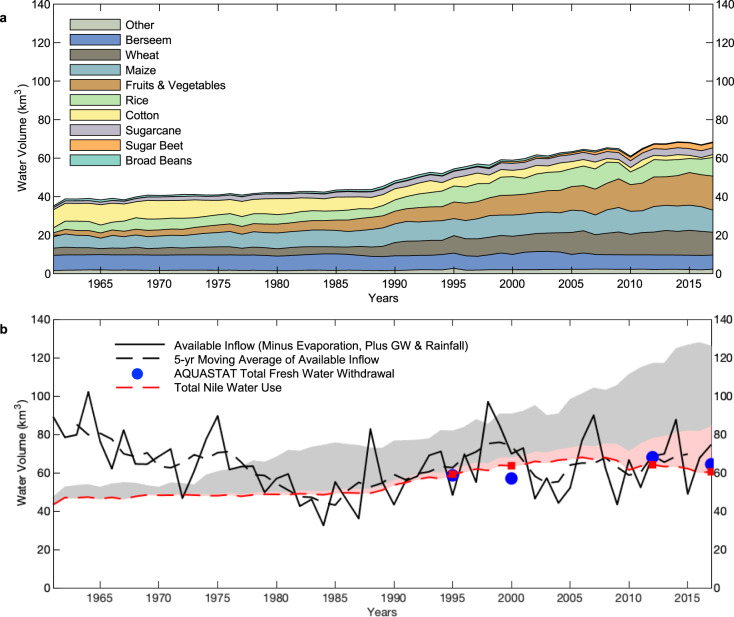
Bottom-up estimates of historical use. **a** Historical water application in Egypt for agriculture broken down by crop (Supplementary Tables 1.1, 1.2, and 1.7). **b** Historical total Nile water use (agriculture + municipal + industrial − reuse) [red dotted line] compared to available supply [black lines]. Red squares mark values for 1995, 2000, 2012, and 2017. This is the flow at Dongola station adjusted by evaporation at Lake Nasser and from other surface water bodies (estimated at 10 km^3^ and 2 km^3^ respectively)^2^, with added rainfall and primary groundwater abstraction (1.5 km^3^ and 0.5 km^3^ respectively)^12, 52^. Storage change in Lake Nasser is not considered in this availability estimate. These are compared to the AQUASTAT numbers for freshwater withdrawal in Egypt [blue dots]^46^ (Supplementary Table 1.16). Dark Gray shading represents the amount of Virtual Water Import (shown in Fig. 2e), and light red shading shows the amount of drainage, wastewater reuse, and GW reuse (shown in Fig. 2c). These shaded areas represent the additional demand met by these sources, and not a range of values.

We apply a bottom up, individual crop-based analysis of agricultural water use (see Methods section) to dissect water demand in Egypt, from in-country production and trade, and identify which crops dominate in-country water use over time (Fig. 3a). This analysis is made possible by the extensive and influential work in water footprint accounting for agriculture at the global and regional scale^31,32^. Figure 3b shows the agricultural use estimates combined with other water demands (municipal, industrial, reuse), compared to the available Nile water flow into Egypt which encompasses all water not consumed upstream. Two major conclusions about Egypt’s water demand are clear from this comparison.

First, Egypt began fully utilizing available local water resources in the late 1970’s and has only met total water demand through increasing virtual water imports (dark gray shading) and increasing reuse (light red shading). Increases in irrigation application efficiency, and to a greater extent water reuse, have improved the water productivity of major crops and allowed Egypt to survive on a relatively constant Nile water use in the past two decades (Supplementary Figs. 3–4).

Second, even after accounting for reuse utilization in Egypt (Fig. 2c) we show that Egypt’s direct consumption of the Nile is roughly 61.5 km^3^ on average from 1988 to 2017 (Fig. 3b). This aligns closely with water accounting in the literature for the modern period^22^. Adding the environmental flow to the Mediterranean of ~2–4 km^3^ (ref. ^33^), Egypt utilizes 8.0–10.0 km^3^ more than the share of 55.5 km^3^ allocated through the 1959 Nile Agreement between Egypt and Sudan^20^. This additional water comes partially from Sudan’s unconsumed share of 1.8–5.5 km^3^ out of the 18.5 km^3^ enumerated in the 1959 Agreement^23^, and partially from increases in the Nile flow of ~5–6 km^3^ (refs. ^17,34,35^). An accounting of Sudan’s historical use of the Nile water is beyond the scope of this Egypt centric study but is reported to be 13–16.7 km^3^/year^18,19,21^. Much of the increased water demand in Egypt has been met by virtual water imports, which reached 40 km^3^ in the 2010’s (Fig. 2e), a figure supported by other studies that quantified historical virtual water trade^22,35,36^.

**Fig. 4:**
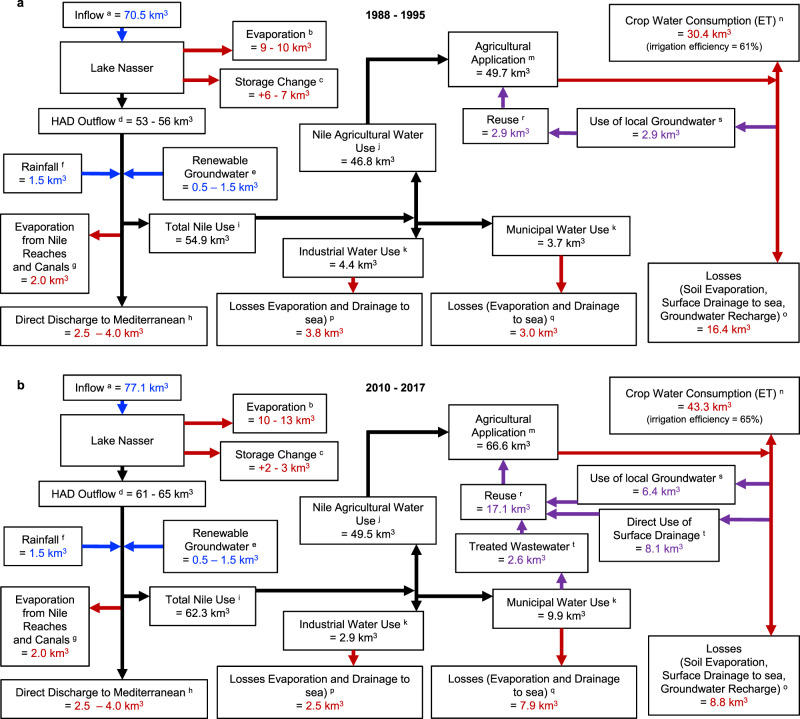
Egypt’s Evolving Water Fluxes. **a** Egyptian Nile water system 1988–1995 average annual fluxes (km^3^/year). Red values are sinks, blue are sources, and purple indicates reuse. **b** Same as panel **a** but for the period 2010–2017. A figure for the average fluxes from 1988 to 2017 can be seen in Supplementary Fig. 16. a. Average annual inflow at Dongola. (Supplementary Table 1.14). b. Average annual Evaporation from Lake Nasser, 10  km^3^ from literature^2^ and outer bound calculated using Lake Nasser height (Supplementary Table 1.17), Height–Volume equation (Supplementary Table 1.15), and CRUTS4.04 Potential Evapotranspiration (Supplementary Table 1.13). c. Average annual storage change calculated using Lake Nasser height (Supplementary Table 1.17 and Height–Volume equation (Supplementary Table 1.15). d. Average annual outflow calculated through water balance of (a)–(b–c). Range of values reflect uncertainty in Lake Nasser balance components. e. AQUASTAT^12^. f. Abdel-Shafy et al., 2010 ^52^. g. Calculated through estimation of surface area and evaporation rate. Confirmed in refs. ^22, 25^. h. Hamza, 2006 ^33^. i. Calculated through methodology described in Methods section. j. (m)–(r). k. AQUASTAT Database^44^ (see Supplementary Fig. 9). m. (n) × Irrigation Application Efficiency (see Supplementary Fig. 5). n. Calculated from production and water consumption data as described in Methods section. o. (m)–(n)–(t)–(u). p. (k) × 0.86. Loss rate from Omar and Moussa, 2016 ^36^. q. (l)  ×  0.80. Loss rate from Omar and Moussa, 2016 ^36^. r. (s) + (t) + (u). See Supplementary Fig. 9. s. Refs. ^5, 51, 53, 54^. t. AQUASTAT Database^44^. (Supplementary Table 1.16).

Virtual water import is calculated identically to in-country use which is detailed in the Methods section. A comparison of the Egyptian Nile water system for 1988–1995 and 2010–2017 is presented in Fig. 4 to further document the evolving dynamics of water supply and demand in Egypt. This schematic relies on several independent sources of data where each flux is uncertain, and hence it does not necessarily satisfy strict water balance. A notable feature is the difference in evaporation from Lake Nasser between the two periods which arises from a 1000 km^2^ difference in lake area (roughly 30%). (Supplementary Tables 1.15 and 1.17).

### Projections of future water demand

Historical analysis has shown that Egypt is fully utilizing the available resources of the Nile River and yet is facing increasing internal and external pressures that will raise water demand and decrease availability of water. As detailed in the Methods section we develop an empirical model of historical and future water demand in Egypt with population growth and economic growth as inputs. Assuming a range of economic and population scenarios (Supplementary Fig. 11), and the empirical relationships between demand for crops and economic growth (Supplementary Fig. 1), we project future water demand for Egypt.

**Fig. 5:**
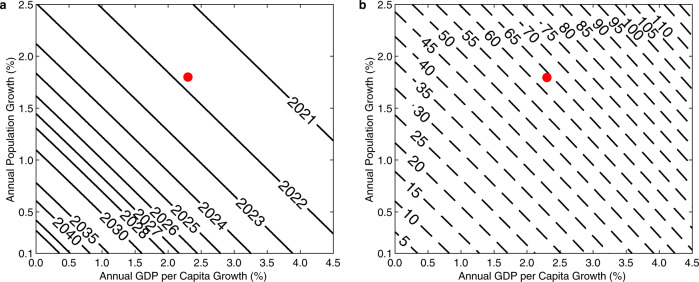
Future projection of demand. **a** Projected Year that Virtual Water Imports will Reach 61.5 km^3^ with population increase and GDP per capita increase. Future increases in demand are added to the Hindcast (model) estimate of total demand minus total estimated Nile use. The red dot marks the nominal scenario of 1.7% population growth and 2.3% GDP per capita growth. **b** Additional virtual water (km^3^) needed in 2030 to satisfy increased demand (i.e. projected virtual water Imports in 2030 values minus 2017 virtual water import amount). The red dot marks the nominal scenario of 1.7% population growth and 2.3% GDP per capita growth.

In keeping with our discussion about Egypt’s characterization as the gift of the Nile, we project when Egypt’s imports of virtual water will reach 61.5 km^3^ (the 30-year average Nile water use for all purposes, accounting for both reuse and application losses), driven by an increase in demand that must be met externally (Fig. 5a). For scenarios that assume population and economic growth rates close to the growth rates over the past 30 years, this important benchmark will be reached in this decade of the 2020s.

**Fig. 6:**
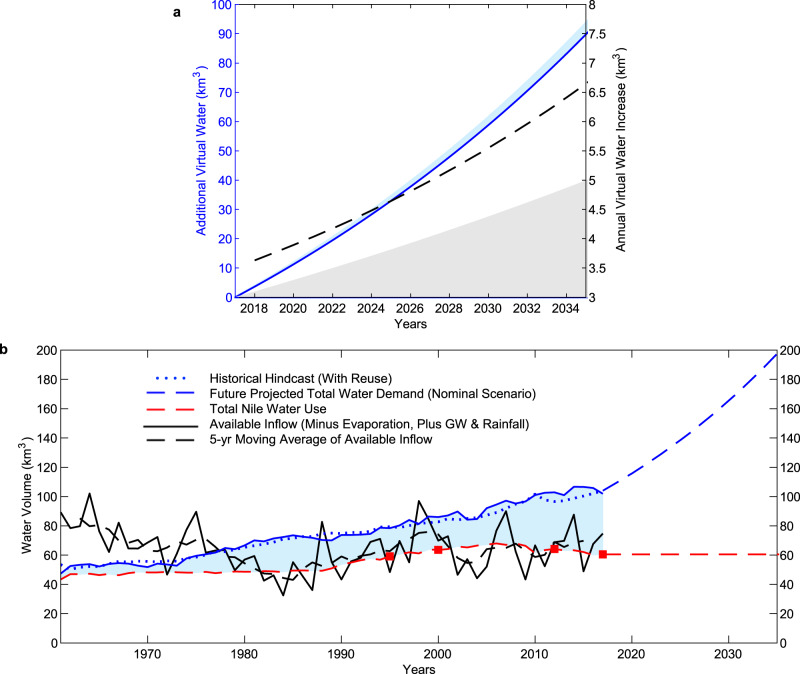
Future scenarios. **a** Additional Virtual Water Demand (km^3^) [blue line] and Annual Increase Rate (km^3^) [black dotted line] for a nominal growth scenario (1.7% population growth, 2.3% GDP per capita growth) versus 2017 levels. Light blue shading represents the increased municipal water demand. Gray shading represents the additional virtual water imports in a 0% GDP growth, 1.7% population growth scenario. **b** Historical hindcast of total water use paired with the nominal projections of total water needed in panel **a**. The blue dotted line shows the total hindcasted water use, including efficiency scaled total agricultural demand, municipal use and industrial use, and reuse. This is compared to the sum of historical virtual water imports [blue shading] and the total Nile use estimate [red dashed line] and is shown as the solid blue line. Future projections are divided between in-country consumption [red dashed line] and increased virtual water demand  [area between red dashed line and blue dashed line]. Future Projected Total Water Demand is shown by blue dashed line. Red squares mark total Nile use estimate for 1995, 2000, 2012, and 2017.

By 2030 the projected trend corresponds to a significant increase in virtual water imports in most population and economic growth scenarios (Fig. 5b). To visualize this process along one growth trajectory, we show the projected virtual water imports for a nominal scenario with a GDP per capita growth rate that matches the 1988–2017 average (2.3%) and a population growth rate that matches the UN Medium Variant population projection in 2035 (1.7%) (Fig. 6a). This is paired with increased demand in the municipal sector, which will need to be met by reallocating internal resources. The rate of increase of virtual water goes up over time (dotted black line) due to the compounding nature of the population and economic growths, as more people enjoy higher consumption rates per capita.

We apply the same demand relationships (Methods) to hindcast the historical water demand using data on population and GDP per capita data. As expected, the hindcasted water demand is close to the sum of the in-country Nile water use (which also accounts for municipal and industrial use and the water reclaimed from reuse) and virtual water import, which together meet the historical demand (Fig. 6b). The future projection is less linear than the historical hindcast, mainly because the hindcast accounts for increasing reuse, which bends the demand curve downwards at an increasing rate in the early 2000’s. The future projection assumes the rate of reuse to be constant post-2017. This is further evidence of the importance of improving irrigation application efficiency. Uncertainty in the projections of future imports of virtual water are discussed in Supplementary Note 1.

## Discussion

Egypt’s responses to increasing water demand in the past have demonstrated the severity of the water scarcity situation, and historical adaptations to rising demand on water will need to continue and strengthen. Efforts targeting an increase in productivity of agriculture should pivot to recognizing and leveraging the true value of water as a limited resource through a robust water pricing system^37^. As virtual water imports increase, smart management of agricultural export and import portfolios can leverage Egypt’s high agricultural yields and amplify their share of natural water resources through the export of high value, high water efficiency crops (fruits and vegetables) and the import of low value, low water efficiency crops (grains). Inter-basin connectivity will be key in the future and using these relationships to import water-intensive commodities like meat can allow allocation of water elsewhere^38^. Finally, studies have shown that a high rate of population growth is one of the most important factors in worsening future water deficits^35^, and further reducing rates of growth through proven methods like healthcare expansion and education^39^ will slow decreases in per capita water share, a key metric of water scarcity. No single solution will be able to compensate for all additional needs.

Increased industrial demand for water will come with economic growth and diversification, and tourism and urbanization will drive municipal demand. As Egypt’s neighbors grow alongside Egypt, they have already begun to exert new pressures. This is especially important with the imminent completion and filling of the Grand Ethiopian Renaissance Dam (GERD). The effect of the GERD was not quantitatively included in our analysis, but is acknowledged as an additional potential stressor, especially with regards to increased upstream withdrawals^21,40^.

Demand and supply of water will also be affected by climate change, although we do not analyze those changes in this study. At a large scale, while climate change will result in small increases in the mean flow of the river, it will increase interannual variability of flow and increase the need for additional storage^34^. Sea level rise and saltwater intrusion are already a threat to freshwater resources in the Nile Delta and will further affect the potability of water for agricultural and municipal purposes^41,42^. Increased temperatures will affect both agricultural productivity and crop suitability, and also increase rates of evaporation from surface water and the field^43^. Optimizing ecological suitability and irrigation systems is crucial for being able to adapt to future changes more easily.

In the framing of this study, we posed the question of whether Egypt will continue to be the gift of the Nile. Historically and culturally the two are synonymous, and Egypt was, is, and will continue to be dependent on the resources the Nile provides. However, historical reconstruction and future projections show that the level of dependence has been and must continue to change. In the near future, Egypt will be dependent on external virtual water to the same level as its level of dependence on the River, and policies and attitudes will need to reflect and adapt to this new reality. Through the reconstruction of Egypt’s water demand we have shown here that Egypt is approaching a threshold between the Nile as a dominant force in sustaining Egypt’s growth and existence, and a new paradigm characterized by an equally important role for basin and global interconnection and cooperation.

Our results illustrate that the future of water in Egypt is just as reliant on external cooperation with its neighbors as it is on its own ability to optimally manage internal demand and use of water. Adaptations are ultimately in Egypt’s best interest, as they allow for continued growth and prosperity with more careful management of resources. Egypt has the chance to be an example for other developing water scarce nations, and a leader in the Nile Basin. If changes are not made it will soon serve as an ecological cautionary tale with implications for the entire region.

## Methods

### Terminology

In this study water use is used to define the water taken from the Nile. Total Use is defined as the water withdrawn for Industrial, municipal, and agricultural purposes. This use is added to reuse in order to determine the total demand for water in the country. In the case of agricultural use, crop water consumption is scaled by irrigation application efficiency, and then reuse is accounted for. Water consumption is based solely on the crop ET water requirement numbers taken from the literature. Our definition of total Nile water use aligns with the AQUASTAT definition of freshwater withdrawal, which is the total withdrawal less desalination, direct use of treated municipal wastewater, and direct use of agricultural drainage water. Our estimate counts subsequent pumping of local groundwater recharge as a reuse component, which is not the case in the AQUASTAT totals.

### Data

All data sources used can be found in Supplementary Table 1. Agricultural and trade data used in this study comes from the Food and Agriculture Organization of the United Nations and is augmented by population and economic data from the World Bank and United Nations Population Division of the Department of Economic and Social Affairs. Water use numbers for available years are obtained from AQUASTAT, a division of the FAO.

The bottom-up analysis focuses on a group of crops that are selected with the goal of including the most significant water consumers. The primary agricultural crops considered in the analysis are wheat, maize, rice, seed cotton, sugarcane, sugar beet, banana, barley, broad beans, berseem, grape, groundnut, olive, onion, orange, potato, sorghum, tomato, dry beans, chickpea, lentils, green beans, lemons and limes, apples, mango, dates, watermelon, tea, sunflower seeds, garlic, strawberry, artichoke, cabbage, carrots & turnips, cauliflower & broccoli, chili pepper (green), cucumber, eggplant, melons, nectarines & peaches, pumpkins & squash, sweet potato, tangerine, vegetables fresh and leguminous (n.e.s.), and soybean. The grouping Fruits & Vegetables include Banana, Orange, Tomato, Potato, Onion, Olive, Lemons & Limes, Apple, Watermelon, Mango, Strawberry, Artichoke, Cabbage, Carrots & Turnips, Cauliflower & Broccoli, Green Chili Peppers, Cucumber, Eggplant, Melons, Peaches & Nectarine, Pumpkin & Squash, Sweet Potato, Tangerine, and other Vegetables Fresh & Leguminous n.e.s. Production, imports and exports focused on the primary commodity (i.e. no juices and processed forms) except when noted below. Secondary crops include cottonseed oil, maize oil, palm oil, raw sugar, molasses, cotton lint, cottonseed cake, sunflower seed cake, and soybean cake. Animal Products include beef, buffalo, sheep, chicken, milk (dried, whole fresh, whole skim), butter (cow & buffalo), eggs (hen in shell), and cheese (buffalo, whole cow, skim cow).

Per capita demand relationships developed for the period 1975-2014 with GDP per capita can be seen in Supplementary Fig. 1 for each individual crop. Only primary products are considered in production (in country) water use numbers for the historical period, to avoid double counting of feed and meat products. In the demand model used to project both future demand and create the historical hindcast, primary product demand is taken as the production and import quantity less the export quantity. Several of the crop products considered are traded primarily in their secondary form. When considering crop production, sugarcane, sugar beet, and seed cotton were used. The secondary products sugar (raw equivalent), molasses, and cotton lint were used in terms of trade demand (imports minus exports). Therefore, products that are primarily exported are already accounted for in the production of the primary crop. Additionally, some crops have demand relationships that are heavily influence by policy decisions – berseem, cotton, and sugarcane–and use a time-based relationship rather than one based on GDP per capita. All estimates of crop water consumption (historical production, historical imports, historical hindcast, and future projections) are scaled by irrigation application efficiency in order to simulate the Egypt equivalent use needed in order to meet the demand for those crops. Therefore, empirical and model-based estimates are consistent.

FAO data was available from 1961 to 2013 for most commodities, and available to 2017 in many cases. In the event that data was not available for the full period, the nearest recorded value to that date was used, and extrapolated outwards to ensure full period coverage for analysis. 2013 values were extrapolated to 2017 for cottonseed cake, soybean cake, sunflower seed cake, and artichoke imports. Berseem area, yield, and production values for 1978 are used for 1961–1977 and 2007 values are used for 2008–2017. Strawberry area, yield, and production values for 1980 are used for 1961–1979. Soybean area, yield, and production values for 1972 are used for 1961–1971. Sugar beet area and yield for 1979 were used for 1961–1978. Sunflower seed area, yield, and production values for 1971 were used for 1961–1970, and sunflower cake data from 1995 was used from 1961 to 1994. 2014 data was expanded to 2017 for Buffalo butter, Cow Butter production, buffalo cheese, skim cow cheese, whole cow cheese production, skim cow milk production, and eggs (hen in shell). Import and Export data for skim cow milk was filled with zeros prior to 1994. Finally large amounts of missing import data was replaced with zeros: pre-2000 for cabbages, all data except 2014/2016 for carrots and turnips, pre-1996 and post 2014 for cauliflower and broccoli, everywhere except 2013–2016 for chili peppers (green), pre-1993 for garlic, 1961–1999 for strawberries, pre-1997 and post-2013 for cucumber, everywhere except 2015 for eggplant, pre- 2005 for melons, in 2014 and 2016 for sweet potato and tangerine and in 2016–2017 for fresh vegetables (n.e.s.). For 2014–2017, food supply data was filled in using the New Food Balances data where available. This may cause slight inconsistencies due to differences in classification or calculation method. Beyond this extrapolation of data to ensure consistent availability of data between commodities, all publicly available data was integrated into the analysis, including FAO estimates. No data was removed due to flagging by the FAO as being an estimation or reconstruction based on a secondary source. Production, yield, and harvested area data for primary crops can be seen in Supplementary Fig. 2.

### Water use numbers

Overall water use numbers for Egypt have low independence between sources and are often available for only a handful of years in the last several decades. Most of the official numbers provided are dictated by the bounds established in the 1959 agreement between Egypt and Sudan where the former was allocated 55.5 km^3^/yr and the latter was allocated 18.5 km^3^/yr. According to flow measurements at Dongola, near the Sudan-Egypt border, the 30-year average flow (1988–2017) was 74.7 km^3^/year. All sources of water except desalination are included in our analysis. Municipal use, industrial use, and reuse values are interpolated and extrapolated from available data and literature values (Supplementary Fig. 9). So far, the increase in per capita water usage seems to have tracked closely with increases in per capita GDP. This growth may have also been driven by the increase in city population. The percentage of the population living in cities decreased from 42.8% in 1995 to 42.7% in 2017 (United Nations), however, because of the increase in population this means that in 2017, roughly 14.5 million more people lived in urban areas compared to 1995. We do assume though that per capita municipal consumption will remain constant in the future at roughly 112 m^3^/capita/year (305.4 liters/capita/day)^44^. A projection of municipal water demand for a range of population projections used in this analysis can be found in the Supplementary Fig. 12.

### Crop water numbers

Crop ET requirement numbers were taken from a single source for consistency and because they were specific to Egypt and the use of irrigated water^45,46^. For commodities that are traded in substantial amounts but not grown in Egypt (e.g. tea), a blue water number taking into account the source of the imports was used^47^. This study is concerned with irrigated water from the Nile and therefore uses only blue water numbers, which refer to the amount of surface and groundwater consumed^45^. In general, the numbers align with the water requirement numbers collected from the FAO which are given in terms of irrigation requirement^48^. Difference likely arise because FAO numbers are non-region or cultivar specific. We use in-country water consumption numbers for all crops and animal products, even when we are exploring virtual water and trade, as we are interested in the magnitude of water that trade replaces and what would otherwise be in-country production and consumption of water. Berseem water use numbers are not available from the same source and are taken from a study on evapotranspiration needs of berseem in northern India, a location at roughly the same latitude and average PET during the growing season^49^. This number is taken as the 2007–2011 average and scaled in the same manner as other crop water requirements discussed below.

In order to determine the water use requirements of different crops through time, we are aware that water application is a factor in determining yield. However, yield growth is also influenced by fertilizer application, climate, seed variety, soil salinity, and other management and ecological characteristics. In order to develop a realistic water requirement function, we use the 1996–2005 average crop ET water requirement from the literature^45^ and the available FAO data on production, area harvested, and yield. Two scenarios for Nile water consumption for historical crop production were devised, both based on the literature value for crop specific, Egypt specific water consumption and the 1996–2005 averages of production and harvested area. The first is an estimate using a constant m^3^/ha for each crop, and the second uses a constant m^3^/tonne value. These estimates, scaled for irrigation application efficiency, can be seen as the blue and red lines in Supplementary Fig. 6. In order to account for factors such as increasing yield and increasing water application, as well as the other yield influencing factors above, we settle on a m^3^/tonne water consumption requirement that equates to the average of these two estimates. This can be seen as the black line in Supplementary Fig. 6. It is important to note that we assume all crops receive exactly their water requirement in any given year. Per tonne water requirements for secondary commodities remain constant due to the absence of comparable yield and area data and use the 1996–2005 average water requirement throughout.

### Historical reconstruction assumptions

We apply a bottom up, individual crop-based estimate of agricultural water consumption and virtual water trade, using the assumptions covered above. This empirical reconstruction of water use is completely independent from official use estimates, and depends only on agricultural data, the physiologically based estimates of water requirement, and an estimation of irrigation application efficiency based on the use of different irrigation technologies. A comparison to AQUASTAT agricultural withdrawal figures adjusted with reuse estimates can be seen in Supplementary Fig. 6.

Adjustments must be made to account for system losses between withdrawal of water from the Nile and field application, known as irrigation application efficiency (water use efficiency at the field scale). This irrigation application efficiency is calculated using proportions of irrigation type and the attributed efficiencies of the technologies^50^. The curve of irrigation application efficiency moves from roughly 61% in the 1960’s to 66% in the mid 2010’s and can be seen in Supplementary Fig. 5. This loss rate is consistent with figures given in the literature^35^. As seen by these low efficiencies, and the small progress made in improving them in the last 50 years, the main true loss in the system is soil evaporation at the field scale.

### Future projection assumptions

Future projections for water demand are made starting in 2017 and shown in this study until 2035. The main assumption in the projections is that all additional demand, which is driven by population increase and GDP per capita increase, will need to be satisfied through imports as Egypt is at its in-country production limit already.

There are two drivers of growth: population increase adds a full person’s worth of demand, and economic growth adds marginal demand to existing populations. The range of population projections considered in the future scenarios span from 0.1% to 2.5% annual growth and encompass the range of growth scenarios given by the United Nations^3^, and the 30-year historical rate falls in this range as well. Similarly, the economic range of scenarios considered was 0.1–4.5% annual growth, which also contained the historical 30-year growth rate. In order to present a single likely scenario to focus on when interpreting the projections, we highlight a nominal scenario. This scenario reflects 2.3% GDP per capita growth to match the last 30 years, and 1.7% population growth to match the UN Medium Variant Projection by 2035^3^.

In order to anchor the future projection and also make it compatible with the hindcast reconstruction, all future values were calculated as additional values versus 2017 levels.

Extrapolation of future demand for water is done through a historical linear regression of GDP per capita and demand or trade demand depending on the commodity from 1975 to 2014. No upper limit is imposed on the estimates of demand. We also hold berseem, seed cotton, and sugarcane per capita demand constant in the future to account for demand changes that are policy rather than growth driven, and to avoid combining an increased demand for meat from out of country and forage crops. The total tonnage demand of these crops will still increase due to population growth. As with historical imports, the water value given is in terms of an Egypt equivalent use by scaling with irrigation application efficiency.

Future projections include water demand coming from agricultural goods, as well as from increased municipal use. Industrial use and reuse were not extrapolated and were held at 2017 levels, since they are influenced more strongly by policy and other drivers not considered in this analysis. To maintain the scaling performed in the historical analysis, future water requirement numbers for individual commodities are the 2012–2017 average water requirement values.

## Supplementary information

Supplementary Info

Peer Review File

Description of Additional Supplementary Files

Supplementary Data 1

Supplementary Movie 1

## Data Availability

All data used in this analysis is publicly available at the locations listed in Supplementary Table 1, with the exception of new streamflow data at Dongola. Dongola streamflow data (1890-2020) used in this study are available from the corresponding author upon reasonable request.
