# Supplementary material for: Past and future trends of Egypt’s water consumption and its sources
Source: Nat Commun. 2021 Jul 23;12:4508. doi: 10.1038/s41467-021-24747-9 (PMC8302683; doi:10.1038/s41467-021-24747-9)
Supplement: Supplementary file 1 — Supplementary Info [file 41467_2021_24747_MOESM1_ESM.pdf]

PAST AND FUTURE TRENDS OF EGYPT'S WATER CONSUMPTION AND ITS SOURCES

Catherine A. Nikiel[1*] and Elfatih A.B. Eltahir[1]

1. Department of Civil and Environmental Engineering, Parsons Laboratory, Massachusetts Institute of Technology, Cambridge, MA 02139

Corresponding Author: nikiel@mit.edu

SUPPLEMENTARY MATERIAL

Including Supplementary Notes (1-2), References (1-20), Tables (1-6), Figures (1-16)

SUPPLEMENTARY NOTES

Supplementary Note 1 | Uncertainty

There are three main areas of input uncertainty in the agricultural analysis: crop water consumption numbers, the accuracy of agricultural commodity data (production, yield, area harvested etc.), and availability of water use data. Mekonnen and Hoekstra (2010) list a number of uncertainties in their calculation of the water consumption numbers including the lack of crop specific irrigation data, the low spatial resolution of crop growth seasons and fertilizer application, the actual level of irrigation supplied, and the effect of factors other than water stress on crop yield [1]. Despite these uncertainties, most of the crops fall within the range of FAO water requirements. FAOSTAT does not release official measures of uncertainty or error for their data as it is aggregated by the individual countries. However, in some cases official data is supplemented with estimates or aggregate data from other sources. As mentioned in the methods and materials, all available data from FAOSTAT was used in the analysis, and missing data was filled in as noted. Only in the calculation of trade value/quantity was the data limited to official numbers only. Finally, only a few data points were available for the construction of time series of municipal and industrial use and reuse. Interpolation was used between the available data, and for reuse, error bounds were added in Figure 2c where data for a component was missing or inconsistent across sources.

The largest component of uncertainty in the historical reconstruction of water use was the scaling of crop water consumption requirement through time. An explanation of several analysis options

is presented in Methods, and a visual comparison is available in Supplementary Figure 6. Additionally, we included most of the main players, but there are some commodities and products not included and the analysis focuses on the production and trade of the primary commodity except where noted.

Supplementary Note 2 | Policies to Meet Future Water Demand and Calculation of Virtual Water Trade Balance (VWTB)

We have shown through our reconstruction that Egypt has taken several measures to manage historical water demand and increase available supply. However, the country is increasingly constrained by its ecological bounds. Throughout the last several decades it has increased land productivity and water productivity, decreased human fertility rates, and increased trade in order to meet demand. However, these measures have only kept Egypt at the edge of satisfying water demand. Egypt reached full utilization of the Nile in the 1980's and has been cutting and compensating ever since. Our reconstruction of past water uses and adaptations allows us to identify avenues for management changes that grow out of historical successes and failures. In order to understand to underscore major principles underlying proposed policies, we recommend the consideration of a hybrid of policies that we have grouped under two conceptual umbrellas:

(i) Water Value Appreciation (WVA) strategies revolve around recognizing and leveraging the true value of water as a limited resource through a robust water pricing system, improving irrigation application efficiency at the field scale, and adjusting cropping patterns to emphasize ecological suitability and water use efficiency.

(ii) Water Share Amplification (WSA) strategies focus on maximizing per capita water share through an approach of smart and efficient management of agricultural import and export (virtual water trade), applicable to arid countries in general, that amplifies Egypt's share of natural water resources. Part of this efficient trade balance includes sourcing meat from neighboring countries and strengthening inter-basin ties.

We also propose further focus on reduction of population growth rate as a means of reducing strain on already scarce per capita water supplies. These six measures will help balance the supply of water and Egypt's growing demand and are discussed further below.

Egypt must place more concrete value on water in the future. Despite Egypt's status as a water scarce nation, there has been a disconnect between this scarcity of water and its price. Water tariffs for residential customers are among the lowest in the world (between \$0.03 and \$0.12 per m^3 in 2017) and are slightly higher for industrial and commercial customers [2]. More problematic is that farmers are not charged for irrigation water [3]. This does not encourage water use efficiency, and actually incentivizes waste, as increased application of water is often thought to boost crop productivity all else being equal. Water pricing for farmers should be tied to either the marginal cost of increasing water supplies or to the cost of increasing water use efficiency though others have noted the obstacles to increasing this efficiency [4].

One of the main changes in attitude that must occur is shifting value from land to water. Land in Egypt is plentiful, and water is not (Supplementary Movie); previous policies have acted as if the opposite is true. From 1980 to 2000, Egypt dramatically increased its crop yields through the use

of more water and the adoption of agricultural technologies such as fertilizer (Figure 2b; Supplementary Fig. 2) [5]. This effort was focused on land productivity (tonnes per unit area), a resource that is much less constrained than water. Water productivity (tonnes/m^3) did not improve as drastically until the more recent aggressive implementation of reuse technologies (Figure 2c; Supplementary Fig. 3-4). Even now, though yields of maize are as high or higher than much of the world, their local water consumption requirement is up to 200 m^3/tonne higher than in the countries where Egypt imports maize from [6]. Egypt has improved their water reuse – from groundwater, wastewater, and direct drainage reuse – but has made comparatively little progress in irrigation application efficiency at the field scale, where the true loss occurs through soil evaporation. This rate of soil evaporation is higher for low-efficiency technologies that do not target the plant specifically and deliver controlled amounts of water that can be fully utilized for transpiration. Reducing conveyance and application losses are key in increasing water productivity in agriculture [7] [8] however there are policy challenges in incentivizing change and ensuring that savings in one sector do not incentivize waste in another [9].

More efficient application necessarily reduces the amount of available reuse. Additionally, attempted increase in reuse in areas such as local groundwater use may lead to unsustainable practices of over pumping. Egypt has made great strides in exploiting available sources of reuse, so much that in 2017 they captured and reused as much as the applied agricultural water that did not go to plant evapotranspiration (Supplementary Fig. 15). Figure 4 shows that municipal and industrial uses are still a viable target for loss reduction, as Omar and Moussa (2016) cite the respective loss rates of these systems at 80% and 86% [7].

Reducing loss in these systems is also important as population growth will create increased demand. Measures need to be taken in order to maximize the available water on a per capita basis. This can take many forms; first Egypt needs to strive further to reduce fertility rates and slow population growth. Although fertility rates have fallen below the average fertility in Africa (3.3 versus 4.4 births per woman in 2018, see Figure 2d) they are still well above the global average of 2.5 [10].

Subsequently, selection of crops that are ecologically suited and easily irrigated using high efficiency measures should be a key goal for future agricultural policy. For example, the expansion of olives is an option as a crop that is well suited to the regional climate and to the use of drip irrigation methods. This expansion is already planned for Egypt, and their efficiency under drip irrigation suggests a promising role in Egypt's export portfolio [11] [12].

Our projections of future demand show that Egypt will also need to rely more on trade, both from outside the Nile basin and from basin trading partners [13]. There is resistance to increasing imports of food products – especially staple goods such as wheat – due to national security concerns, as well as the importance of agriculture in the labor sector [14]. The 2012-2017 FAO Country Programming Framework (CPF) proposes ambitious targets to increase self-sufficiency by 2030 [15]. However, food security priorities are best coordinated with efforts to decrease water demand in order to increase overall resilience. This is especially important in light of difficulties imposed by the COVID-19 pandemic. Currently 69% of Egypt's main import, wheat, comes from Russia, and roughly 90% from Russia and former Soviet Republics [16]. As exporters of staple products, such as Russia, cut back in light of their own production concerns

during the coronavirus pandemic [17], Egypt needs to re-examine their relationships and dependencies. Future import policies need to ensure that Egypt is not totally dependent on one import or export crop, and not dependent on one country for buying or selling goods.

One opportunity for Egypt to increase their imports of virtual water while saving water internally is through the import of meat from neighboring countries, particularly Sudan. In 2007-2011 Egypt consumed an average of 4.9 km^3 (7.4 km^3 withdrawn) on in-country production of Berseem, also known as Egyptian clover, which is a major feed crop. In total, feed crops consumed in 2010 had a consumptive demand of 15 km^3 (Supplementary Fig. 10). At the same time their import of red meat (beef and buffalo) has not increased substantially despite an increasing appetite (Supplementary Fig. 8). On average in 1998-2004, Egypt imported the equivalent of 18.3 Mm^3 of virtual water in sheep from Sudan, although livestock movement across the Egypt-Sudan border is often under documented [18]. Sudan currently raises livestock almost entirely under rain-fed, nomadic conditions although this could change with future irrigation development [19]. Reducing cultivated area of berseem and importing meat instead would save water, free up land, and increase inter-basin ties that could encourage greater cooperation down the road on water and economic issues. There are caveats to this approach, as Berseem has long been used as a rotation crop with other cereals to preserve and renew soil quality [20].

We investigate the Virtual Water Trade Balance (VWTB) as a concept of maximizing the water available to a country, both in terms of water productivity and in terms of market value. Using the trade relationships between crops, we can quantify the impact of shifting production away

from water intensive but relatively cheap to import goods, and instead focus on high value crops that have lower water consumption requirements or are better candidates for higher efficiency irrigation technologies.

In order to develop this policy proposal, a tabulation of the tradeoffs between crops was necessary. A calculation of overall VWTB for exports and imports can be found in the Supplementary Table 2 and uses data from FAO for 2007-2011. In order to avoid inconsistencies in reporting over such a short period, only years with official data were used in the calculation of trade value and quantity. A crop-by-crop comparison table can be found in the Supplementary Table 4, and was calculated using the following formula:

VWTB = (tonnes of Crop A purchased using 1 km^3 of water to grow and sell crop B) / (tonnes of crop A grown with 1 km^3 of water)

In a hypothetical trade transaction in which all the revenue from exporting crop B is used to import crop A, VWTB is calculated as the ratio of the virtual water volume imported and embodied in crop A per unit volume of virtual water exported and embodied in crop B. Simply, the VWTB number given is a measure of units of virtual water imported for each unit of water exported.

The VWTB for Egypt overall is currently 2.31 (Supplementary Table 2). Meaning that they are making 2.31 times more revenue than they are spending, per unit of consumed water traded, or in other terms each unit volume of virtual water exported facilitates 2.31 units of virtual water

imports. Using a set of water share amplification metrics calculated for current crops produced and traded we develop a policy proposal presented in Supplementary Table 5. Additional considerations of irrigation method may make some crops more advantageous, but only if irrigation systems are well maintained and operated to reduce losses.

Regarding the suite of policy umbrellas proposed above, no single solution will be able to compensate for all additional needs. Measures must work to mitigate internal demand (water pricing, reduced fertility rates, and optimized cropping patterns), increase internal supply (through improving irrigation application efficiency at the field scale), as well as leverage Egypt's advantages to form stronger ties with neighbors and the global market.

SUPPLEMENTARY TABLES

Supplementary Table 1: DATA SOURCES AND DOWNLOAD LINKS

1	Crop Water Requirements (Except Berseem, Tea, Palm Oil)	Mekonnen, M.M. and Hoekstra, A.Y. The green, blue and grey water footprint of crops and derived crop products, Value of Water Research Report Series No. 47, Volume II, Appendix II (UNESCO-IHE, Delft, the Netherlands., 2010) https://research.utwente.nl/files/59480760/Report47_WaterFootprintCrops_Vol2.pdf
2	Crop Water Requirements (Tea, Palm Oil)	Mekonnen, M.M. and Hoekstra, A.Y. National water footprint accounts: the green, blue and grey water footprint of production and consumption, Value of Water Research Report Series No. 50, Volume II, Appendix VI (UNESCO-IHE, Delft, the Netherlands, 2011) https://research.utwente.nl/files/5146139/Report50-NationalWaterFootprints-Vol2.pdf.pdf
3	Animal Product Water Requirements	Mekonnen, M.M. and Hoekstra, A.Y. The green, blue and grey water footprint of farm animals and animal products, Value of Water Research Report Series No. 48, Volume II, Appendix V, Grazing Method (UNESCO-IHE, Delft, the Netherlands, 2010) https://research.utwente.nl/files/59481201/Report48_WaterFootprint_AnimalProducts_Vol2.pdf
4	Population Data (Historical & Projection)	United Nations, Department of Economic and Social Affairs, Population Division. Total population (both sexes combined) by region, subregion and country, annually for 1950-2100 (thousands)). World Population Prospects 2019, Online Edition. Rev. 1. (File POP/1-1) (2019). Available Online at https://population.un.org/wpp/Download/Files/1_Indicators%20(Standard)/EXCEL_FILES/1_Population/WPP2019_POP_F01_1_TOTAL_POPULATION_BOTH_SEXES.xlsx https://ourworldindata.org/grapher/absolute-increase-global-population https://ourworldindata.org/grapher/population https://ourworldindata.org/grapher/world-population-1750-2015-and-un-projection-until-2100
5	Fertility Data	United Nations, Department of Economic and Social Affairs, Population Division. Total fertility by region, subregion and country, 1950-2100 (live births per woman). World Population Prospects 2019, Online Edition. Rev. 1. (File FERT/4). (2019) https://population.un.org/wpp/Download/Files/1_Indicators%20(Standard)/EXCEL_FILES/2_Fertility/WPP2019_FERT_F04_TOTAL_FERTILITY.xlsx
6	Economic Data	http://www.fao.org/faostat/en/#data/MK

7	Production, Yield, Area Harvested	http://www.fao.org/faostat/en/#data/QC<br>http://www.fao.org/faostat/en/#data/QD<br>http://www.fao.org/faostat/en/#data/QA<br>http://www.fao.org/faostat/en/#data/QL<br>http://www.fao.org/faostat/en/#data/QP
8	Berseem Production, Yield, Area Harvested	Dost, M., Bimal, M., El-Nahrawy, M., Khan, S., & Serkan, A. Egyptian Clover (Trifolium alexandrinum). King of Forage Crops. (Food and Agriculture Organization of the United Nations, Regional Office for the Near East and North Africa, Cairo, 2014) http://www.fao.org/3/a-i3500e.pdf
9	Animal Carcass Weights	http://www.fao.org/faostat/en/#data/QL
10	Feed & Food Supply	http://www.fao.org/faostat/en/#data/BC<br>http://www.fao.org/faostat/en/#data/BL<br>http://www.fao.org/faostat/en/#data/CC<br>http://www.fao.org/faostat/en/#data/CL<br>http://www.fao.org/faostat/en/#data/FBSH<br>http://www.fao.org/faostat/en/#data/FBS
11	Import and Export Quantity & Value	http://www.fao.org/faostat/en/#data/TP<br>http://www.fao.org/faostat/en/#data/TA
12	Cereal Import Dependency and other Food Security Indicators	http://www.fao.org/faostat/en/#data/FS
13	Potential Evapo-Transpiration Data	CRU TS 4.04<br>https://crudata.uea.ac.uk/cru/data/hrg/cru_ts_4.04/<br>https://crudata.uea.ac.uk/cru/data/hrg/cru_ts_4.04/cruts.2004151855.v4.04/pet/
14	Dongola Inflow Data	Republic of Sudan Ministry of Irrigation and Water Resources (2020). Monthly Streamflow at Dongola (1963-2020). Processed and Compiled By Elzeinn, A. S.
15	Height-Volume and Height Volume Equations for Lake Nasser	Shafik, N. M. Updating the surface area and volume equations of Lake Nasser using multibeam system. Presented at 19th International Water Technology Conference (2016) [http://iwtc.info/wp-content/uploads/2016/05/41.pdf]
16	Egypt Water Withdrawals	Food and Agriculture Organization of the United Nations (FAO). AQUASTAT Main Database. (2016) http://www.fao.org/nr/water/aquastat/data/query/index.html?lang=en<br>Food and Agriculture Organization of the United Nations. AQUASTAT Country Profile – Egypt. Water Resources Factsheet. (Rome, Italy, 2016).

		Available Online at https://storage.googleapis.com/fao-aquastat.appspot.com/countries_regions/factsheets/water_resources/en/EGY-WRS.pdf<br>Food and Agriculture Organization of the United Nations (FAO). AQUASTAT Country Profile – Egypt. (Rome, Italy, 2016). Available Online at http://www.fao.org/3/i9729en/I9729EN.pdf
17	Lake Nasser Levels	Gammal, E. A. E., Salem, S.M., and Gammal, A. E. A. E. Change detection studies on the world's biggest artificial lake (Lake Nasser, Egypt). *The Egyptian Journal of Remote Sensing and Space Science,* **13** (2) 89-99, (2010). [https://doi.org/10.1016/j.ejrs.2010.08.001.]<br>NASA/USDA. G-REALM – Lake Nasser (0331) Height Variations from Altimetry<br>https://ipad.fas.usda.gov/cropexplorer/global_reservoir/gr_regional_chart.aspx?regionid=metu&reservoir_name=Nasser
18	Irrigation Usage	FAO. 2016. AQUASTAT Main Database, Food and Agriculture Organization of the United Nations: Irrigation and Drainage Development. http://www.fao.org/aquastat/statistics/query/index.html?lang=en
19	Storage Capacity	FAO. 2016. AQUASTAT Country Profile – Egypt. Food and Agriculture Organization of the United Nations (FAO). Rome, Italy https://storage.googleapis.com/fao-aquastat.appspot.com/Excel/dams/EGY-dams_eng.xlsx
20	Aswan Monthly Discharge	Vörösmarty, C.J., Fekete, B. M. & Tucker, B. A. River Discharge Database, Version 1.1 (RivDIS v1.0 supplement). Aswan Dam Egypt. Available through the Institute for the Study of Earth, Oceans, and Space / University of New Hampshire, Durham NH (USA). (1998) http://dx.doi.org/10.3334/ORNLDAAC/199

Supplementary Table 2: CURRENT VIRTUAL WATER TRADE BALANCE (VWTB)

Monetary value, tonnage, and water equivalent of agricultural import and export in Egypt, using official trade values (2007-2011 average) (Source: FAOSTAT)

	Total Value (billion USD)	*Total Water Consumed (km^3)*	*Total Trade Quantity (million tonnes)*	*Total Water Value (Million USD/km^3)*
Imports	7.1	19.2	18.2	368.4 (a)
Exports	1.8	2.1	2.8	850.9 (b)
			**VWTB**	**2.31**

Supplementary Table 3: CROP AND ANIMAL PRODUCT DATA Key crop and animal product figures and water usage estimates (2007-2011 average). * Indicates commodities where the 1996-2005 water consumption value was used in absence of yield and harvested area data for scaling. All total water use estimates are given in terms of consumption (without irrigation efficiency scaling). Import and Export Price/Quantity are computed individually for all years with official data and then averaged over the period. (Source: Import, Export and Production data from FAOSTAT; Crop water Requirement data from [a] except for Berseem where the water requirement is calculated using figures from [b], Animal Product water requirement from [c], and water requirement for tea and palm oil from [d])

See Supplementary Data: Table 3

Supplementary Table 4: VIRTAL WATER TRADE BALANCE MATRIX Rows are exports and columns are imports. Irrigation efficiency not considered. Values used for calculation located in Supplementary Table 3.

	Maize	Wheat	Rice	Soybean	Broad Beans	Barley	Groundnuts (In Shell)	Sorghum	Beef *	Chicken *	Cottonseed Oil *	Maize Oil *
Maize	1.12	0.90	0.77	1.29	0.65	1.85	0.30	0.32	1.05	0.59	2.03	0.59
Wheat	1.56	1.26	1.08	1.80	0.91	2.59	0.43	0.45	1.47	0.83	2.85	0.82
Rice	2.23	1.79	1.54	2.57	1.30	3.69	0.61	0.65	2.09	1.18	4.07	1.18
Sugar beet	14.99	12.06	10.32	17.26	8.76	24.81	4.09	4.36	14.06	7.91	27.31	7.91
Sugar (Raw)	1.87	1.51	1.29	2.16	1.10	3.10	0.51	0.55	1.76	0.99	3.41	0.99
Soybean	3.60	2.90	2.48	4.15	2.11	5.96	0.98	1.05	3.38	1.90	6.56	1.90
Cotton Lint	1.49	1.20	1.02	1.71	0.87	2.46	0.41	0.43	1.40	0.78	2.71	0.78
Orange	3.59	2.89	2.47	4.14	2.10	5.95	0.98	1.04	3.37	1.90	6.54	1.89
Tomato	10.87	8.75	7.49	12.52	6.36	18.00	2.96	3.16	10.20	5.74	19.81	5.74
Potato	5.64	4.54	3.88	6.49	3.30	9.33	1.54	1.64	5.29	2.98	10.28	2.97
Banana	4.74	3.81	3.26	5.45	2.77	7.84	1.29	1.38	4.44	2.50	8.63	2.50
Broad Beans	2.49	2.00	1.71	2.87	1.46	4.12	0.68	0.72	2.34	1.31	4.54	1.31
Barley	0.67	0.54	0.46	0.78	0.39	1.12	0.18	0.20	0.63	0.36	1.23	0.36
Groundnuts (In Shell)	2.00	1.61	1.38	2.30	1.17	3.31	0.54	0.58	1.87	1.05	3.64	1.05
Sorghum	3.81	3.06	2.62	4.39	2.23	6.30	1.04	1.11	3.57	2.01	6.94	2.01
Olive	2.53	2.04	1.74	2.91	1.48	4.19	0.69	0.74	2.37	1.34	4.61	1.33
Onion	7.44	5.99	5.12	8.57	4.35	12.32	2.03	2.16	6.98	3.93	13.56	3.93
Beef *	1.50	1.20	1.03	1.72	0.88	2.48	0.41	0.44	1.40	0.79	2.73	0.79
Chicken *	2.70	2.17	1.86	3.11	1.58	4.47	0.74	0.79	2.53	1.43	4.92	1.43
Beans, Dry	1.89	1.52	1.30	2.17	1.10	3.12	0.51	0.55	1.77	0.99	3.43	0.99
Chickpea	2.90	2.33	2.00	3.34	1.69	4.80	0.79	0.84	2.72	1.53	5.28	1.53
Lentil	0.82	0.66	0.56	0.94	0.48	1.35	0.22	0.24	0.77	0.43	1.49	0.43
Beans, Green	12.83	10.32	8.83	14.77	7.50	21.23	3.50	3.73	12.03	6.77	23.37	6.76
Lemons and Limes	3.74	3.01	2.57	4.30	2.18	6.18	1.02	1.09	3.50	1.97	6.81	1.97
Apple	4.58	3.68	3.15	5.27	2.68	7.57	1.25	1.33	4.29	2.42	8.34	2.41
Watermelon	22.24	17.89	15.31	25.61	13.00	36.81	6.06	6.47	20.86	11.74	40.52	11.73
Mango	1.19	0.95	0.82	1.37	0.69	1.96	0.32	0.35	1.11	0.63	2.16	0.63
Molasses *	5.08	4.09	3.50	5.85	2.97	8.41	1.39	1.48	4.77	2.68	9.26	2.68
Cottonseed Oil *	0.99	0.80	0.68	1.14	0.58	1.64	0.27	0.29	0.93	0.52	1.80	0.52
Maize Oil *	1.90	1.53	1.31	2.19	1.11	3.14	0.52	0.55	1.78	1.00	3.46	1.00
Garlic	11.24	9.04	7.74	12.94	6.57	18.60	3.06	3.27	10.54	5.93	20.48	5.93
Strawberry	10.69	8.60	7.36	12.30	6.25	17.68	2.91	3.11	10.02	5.64	19.47	5.64
Artichoke	5.69	4.57	3.92	6.55	3.32	9.41	1.55	1.65	5.33	3.00	10.36	3.00
Cabbage	11.93	9.59	8.21	13.73	6.97	19.74	3.25	3.47	11.19	6.29	21.73	6.29
Carrot & Turnip	2.24	1.80	1.54	2.58	1.31	3.70	0.61	0.65	2.10	1.18	4.07	1.18
Cauliflower & Broccoli	18.84	15.15	12.97	21.69	11.01	31.17	5.14	5.48	17.67	9.94	34.32	9.93
Chili Pepper Green	19.99	16.08	13.76	23.02	11.69	33.09	5.45	5.81	18.75	10.55	36.42	10.54
Cucumber	18.40	14.80	12.67	21.19	10.76	30.45	5.02	5.35	17.26	9.71	33.52	9.70
Eggplant	11.18	8.99	7.69	12.87	6.53	18.50	3.05	3.25	10.48	5.90	20.36	5.89
Melon	13.75	11.06	9.46	15.83	8.04	22.75	3.75	4.00	12.90	7.25	25.05	7.25
Peaches & Nectarine	2.75	2.21	1.89	3.17	1.61	4.55	0.75	0.80	2.58	1.45	5.01	1.45
Pumpkin, Squash	7.58	6.10	5.22	8.73	4.43	12.55	2.07	2.20	7.11	4.00	13.81	4.00
Sweet Potato	16.38	13.18	11.28	18.86	9.57	27.11	4.47	4.76	15.36	8.64	29.84	8.64
Tangerines, Mandarin, Clementine	3.93	3.16	2.71	4.53	2.30	6.50	1.07	1.14	3.69	2.07	7.16	2.07
Vegetable Fresh Nes	13.09	10.53	9.01	15.08	7.65	21.67	3.57	3.81	12.28	6.91	23.85	6.90

Supplementary Table 5: VIRTUAL WATER TRADE BALANCE POLICY PROPOSAL

Policy Proposal for doubling exports. The principle for reduction is a proportional decrease based on the current amount of water currently used in Egypt to produce cereal crops. The principle for increase in export goods is a proportional increase in the amount of exports (i.e. increase the export of the selected goods by x%). Ultimately, a doubling of the export goods below produces enough revenue to buy cereal goods at a water share amplification of 4.4. (51.7 Mm^3 are consumed to buy cereal crops that would normally consume 280.3 Mm^3). Values used in calculation can be seen in Supplementary Table 3. Irrigation Method and efficiency are not considered.

Crop to Increase	Additional Exports (thousand tonnes)	Export Price (USD/m^3)	Money to Make (Million USD)	Water Needed (Mm^3)
Tomato	46.0	$3.16	20.6	6.5
Green Beans	33.6	$3.73	33.4	9.0
Watermelon	20.7	$6.47	12.3	1.9
Molasses	12.2	$3.27	6.6	6.6
Strawberry	76.2	$3.11	66.9	21.5
Cabbage	1.8	$3.47	1.3	0.4
Cauliflower & Broccoli	2.0	$5.48	1.9	0.3
Chili Pepper (Green)	10.0	$5.82	9.0	1.6
Cucumber	1.0	$5.35	1.1	0.2
Eggplant	0.5	$3.25	0.5	0.1
Melon	8.9	$4.00	6.0	1.5
Sweet Potato	19.6	$4.77	10.3	2.2
**Totals**	232.5		169.9	51.7

Crop to Reduce	Amount to reduce Production (Mm^3)	Reduction in Production (thousand tonnes)	Money Needed to Import Replacement (Million USD)
Wheat	62.5	69.4	22.6
Maize	65.8	62.0	19.1
Rice	51.1	52.1	22.5
Barley	2.7	1.3	0.5
Sorghum	3.6	7.1	3.6
Berseem	94.6	29.0 (Beef Reduction)	101.6
**Totals**	280.3	220.8	169.9
		**Trade Balance**	4.4

Supplementary Table 6: **R^2 FOR PER CAPITA DEMAND RELATIONSHIPS (1975-2014 GDP PER CAPITA).** Relationships for berseem, sugarcane, and seed cotton are done versus time instead of GDP per capita. * indicates significance at p = 0.05 ** indicates significance at p = 0.01

Commodity	R^2	Commodity	R^2
Maize	0.83 **	Bean (Dry)	0.13 *
Wheat	0.5 **	Chickpea	0.00
Rice	0.22 **	Lentil	0.22 **
Seed Cotton	0.89 **	Bean (Green)	0.00
Sugarcane	0	Lemon & Lime	0.1 *
Sugar beet	0.86 **	Apple	0.77 **
Banana	0.92 **	Mango	0.80 **
Barley	0.35 **	Date	0.77 **
Broad Bean	0.03	Watermelon	0.19 **
Berseem	0.84 **	Sunflower Seed	0.22 **
Grape	0.80 **	Garlic	0.04
Groundnut	0.67 **	Strawberry	0.90 **
Olive	0.87 **	Vegetable Leguminous n.e.s.	0.47 **
Onion, Dry	0.07	Artichoke	0.60 **
Orange	0.06	Cabbage	0.06
Potato	0.63 **	Carrot & Turnip	0.13 *
Tomato	0.77 **	Cauliflower & Broccoli	0.27 **
Sorghum	0.57 **	Chili Pepper (Green)	0.79 **
Soybean	0.69 **	Cucumber	0.22 **
Eggplant	0.79 **	Cotton Lint	0.50 **
Melon	0.41 **	Cottonseed Cake	0.05
Peach & Nectarines	0.54 **	Sugar (Raw Eq.)	0
Pumpkin & Squash	0.17 **	Molasses	0.14 *
Sweet Potato	0.62 **	Soybean Cake	0.36 **
Tangerine, Clementine, & Mandarin	0.89 **	Cottonseed Oil	0.75 **
Vegetables Fresh n.e.s.	0.04	Maize Oil	0.61 **
Beef	0.03	Palm Oil	0.43 **
Buffalo	0.02	Tea	0
Chicken	0.01	Sunflower Cake	0.45 **
Sheep	0.0	Milk (Whole)	0.49 **
Cheese	0.46 **	Milk (Skim)	0.38 **
Butter	0.00	Milk (Dried)	0.23 **
Eggs (Hen, In Shell)	0.17 **		

SUPPLEMENTARY FIGURES

SUPPLEMENTARY FIG. 1: DEMAND RELATIONSHIPS Demand per capita (kg/capita/year) vs. GDP per capita (2010 USD/capita/year) relationships for individual crops (except sugarcane, berseem, and seed cotton which are time dependent relationships).

SUPPLEMENTARY FIG. 2: PRODUCTION, YIELD AND HARVESTED AREA Change relative to 1961 (%) of production (tonnes) [blue], yield (hg/ha) [black], and harvested area (ha) [red] of primary crops used in analysis.

SUPPLEMENTARY FIG. 3: CROP WATER REQUIREMENTS Water consumption requirements for individual crops used in historical reconstruction (m^3/tonne). The red square indicates the base water consumption value from Mekonnen and Hoeskstra (2010) [30] for the 1996-2005 period.

SUPPLEMENTARY FIG. 4: FACTORS IMPACTING CROP WATER REQUIREMENTS (a) Water consumption requirement for major water consuming crops scaled using 1961 irrigation application efficiency (tonnes/m^3). 1961 values for wheat, maize, rice, cotton, berseem, and sugarcane are 0.0006, 0.0004, 0.0007, 0.0002, 0.0043, and 0.006 tonnes/m^3 respectively. (d) Evolving irrigation efficiency scaled water consumption requirement for major water consuming crops (tonnes/m^3). (e) Evolving irrigation efficiency scaled water consumption requirement for major water consuming crops also including proportion of reuse (tonnes/m^3).

SUPPLEMENTARY FIG. 5: AGRICULTURAL LAND AND IRRIGATION TECHNOLOGY IN EGYPT Development of agricultural area in Egypt and use of irrigation technologies. (a) Old vs. New Land agricultural area (ha) in Egypt using total cropland data (FAO) and details on new land acquisition (Source: Water and Agriculture in Egypt, 2011) (b) Proportion of land using various irrigation technologies (%). Values are available for 1995, 2000, and 2010 and interpolated between (Source: AQUASTAT). (c) Estimate of country average irrigation efficiency (%) determined using the proportion of irrigation type in panel b and the FAO stated efficiency of the relative irrigation technologies.

SUPPLEMENTARY FIG. 6: METHODS FOR RECONSTRUCTING AGRICULTURAL WATER USE Historical water use (km^3) for agriculture. Green data show official data for agricultural use (Source: AQUASTAT). Error bars represent literature estimates of agricultural drainage reuse, groundwater recharge reuse, and wastewater reuse with the assumption that all reuse is applied to agricultural purposes. The lines show bottom up crop-data based estimates for agricultural use using three different crop water consumption assumptions: 1) constant tonnage water requirement (m^3/tonne) [blue] 2) constant area water requirement (m^3/ha) [red] and 3) average of (1) and (2) (m^3/tonne) [black solid]

SUPPLEMENTARY FIG. 7: AGRICULTURAL IMPORTS Total import tonnage (million tonnes) and breakdowns of maize, wheat, and soybean imports (million tonnes).

SUPPLEMENTARY FIG. 8: PER CAPITA WATER CONSUMPTION a) Historical per capita water consumption (m^3/capita/year) for agricultural production [black solid] and historical per capita water embodied in primary and secondary crop imports (m^3/capita/year) [black dashed].

SUPPLEMENTARY FIG. 9: NON-AGRICULTURAL WATER USES AND REUSE Historical estimates for a) municipal use (km^3) and industrial use (km^3) and b) three components of reuse (km^3)

SUPPLEMENTARY FIG. 10: FEED AND ANIMAL PRODUCTS a) Feed products in Egypt (tonnes) b) Estimates of water consumption (km^3) associated with feed products in panel (a) vs. consumption associated with animal product production in Egypt (meat, milk, and eggs) c) Total virtual water import (km^3) (consumption equivalent) associated with animal products [black] and historical per capita water embodied in animal product imports (m^3/capita/year) [black dashed]

SUPPLEMENTARY FIG. 11: PER CAPITA GDP AND POPULATION SCENARIOS (a) High and low projections of GDP per capita growth (%) used in the study (black) along with the projection that follows the past 30-yr average growth (red). (b) High and low projections of population used in the study (black) alongside the high, medium, and low variant UN population projections (blue). Also plotted is the projection that follows the UN Medium projection scenario (red).

SUPPLEMENTARY FIG. 12: MUNICIPAL WATER USE PROJECTIONS Municipal water use projections based on population projections in Supplementary Fig. 11b (km^3)

SUPPLEMENTARY FIG. 13: FUTURE DEMAND TONNAGE Future additional demand (million tonnes) in a given year vs. 2017 for primary crops and cotton lint bounded by two scenarios: 4.5% GDP per capita growth and 2.5% population growth [black] and 0% GDP per capita growth and 0.1% population growth [blue]. The nominal growth scenario (2.3% GDP per capita growth and 1.7% population growth is shown in red.

SUPPLEMENTARY FIG. 14: CEREAL IMPORT DEPENDENCY a) Cereal Import Dependency Ratio (%) using FAO data (2000-2017) and projected data for maize, wheat, rice, barley, and sorghum in the nominal scenario (2.3% GDP per capita growth and 1.7% population growth) (2017-2035). This is compared to the historical ratio for Japan [blue] and S. Korea [red]. b) 2003-2012 Avg. Cereal Import Dependency Ratio vs. 2003-2012 Avg. GDP per capita. African countries [red] and other select countries important for import and export relationships [black], Projected ratio for Egypt [blue x] in the nominal scenario (2018-2035).

SUPPLEMENTARY FIG. 15: REUSE AND EFFICIENCY a) Volume of water withdrawn for agriculture but not used directly for crop evapotranspiration [black] and amount of reuse (treated municipal wastewater, direct use of agricultural drainage, use of local groundwater recharge) [red]. Note that the reuse exceeds the water surplus because a small amount of reuse is initially withdrawn for municipal purposes (treated municipal wastewater). b) a measure of the true system efficiency by using net losses (application losses minus reuse).

SUPPLEMENTARY FIG. 16: 1988-2017 EGYPTIAN NILE WATER SYSTEM FLUXES: Egyptian Nile Water System 1988-2017 average annual fluxes (km^3/yr). Red values are sinks, blue are sources, and purple indicates reuse. See Figure 4 for Data Sources.

SUPPLEMENTARY FIG. 1: DEMAND RELATIONSHIPS Demand per capita (kg/capita/year) vs. GDP per capita (2010 USD/capita/year) relationships for individual crops (except sugarcane, berseem, and seed cotton which are time dependent relationships). Data Sources: (4) (6) (7) (8) (11)

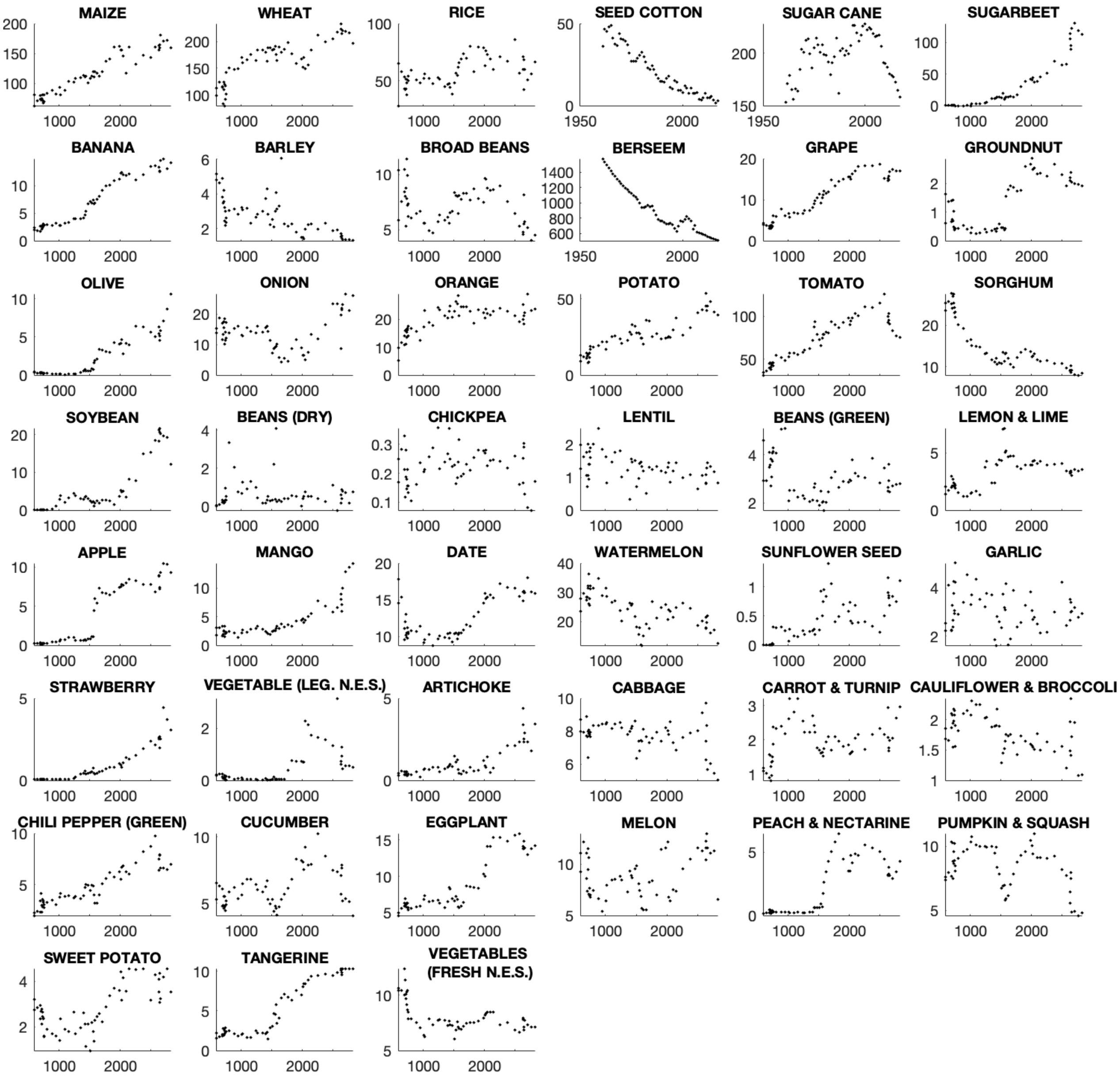

SUPPLEMENTARY FIG. 2: PRODUCTION, YIELD, AND HARVESTED AREA Change relative to 1961 (%) of production (tonnes) [blue], yield (hg/ha) [black], and harvested area (ha) [red] of primary crops used in analysis. Data Sources: (7) (8)

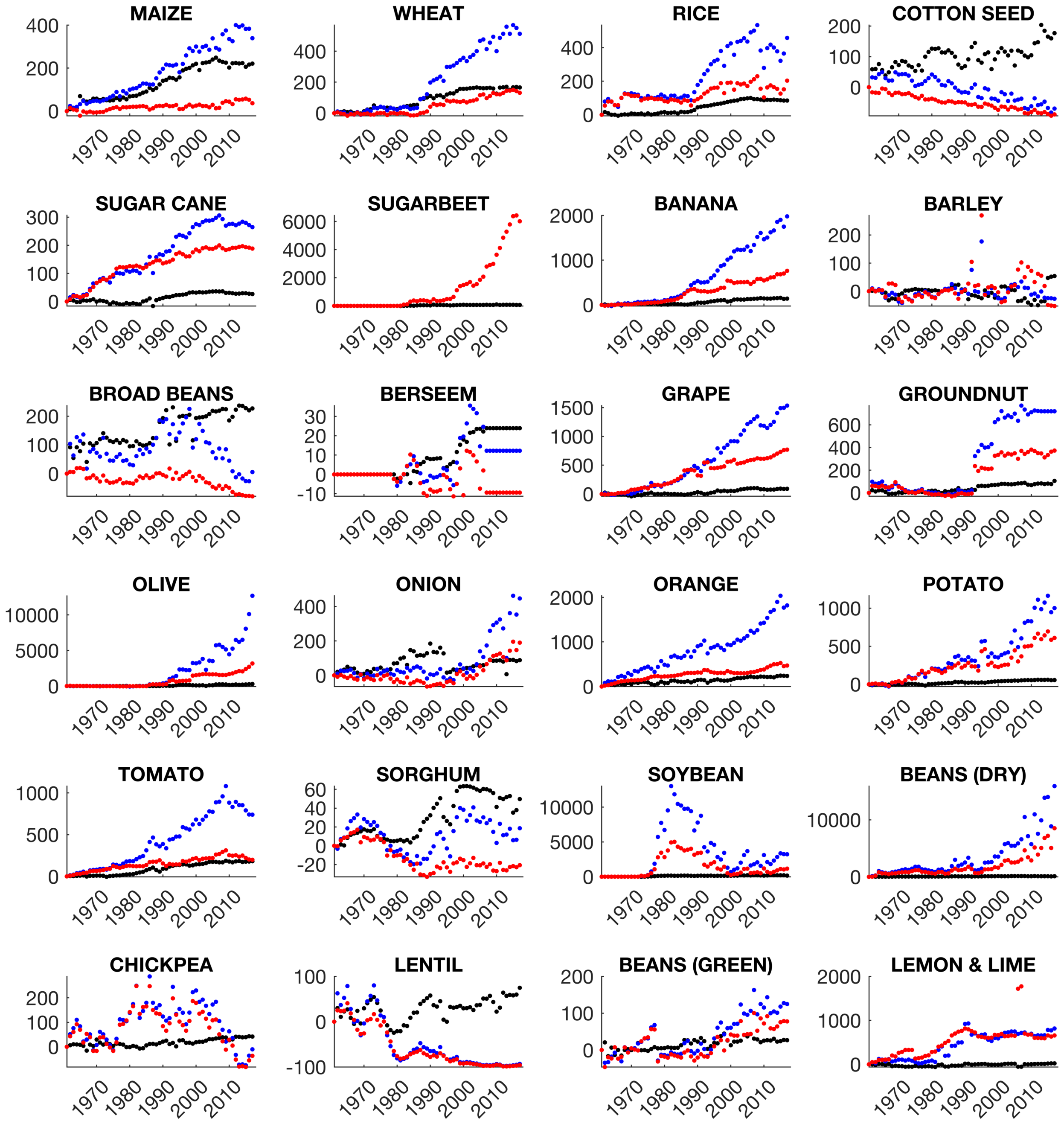

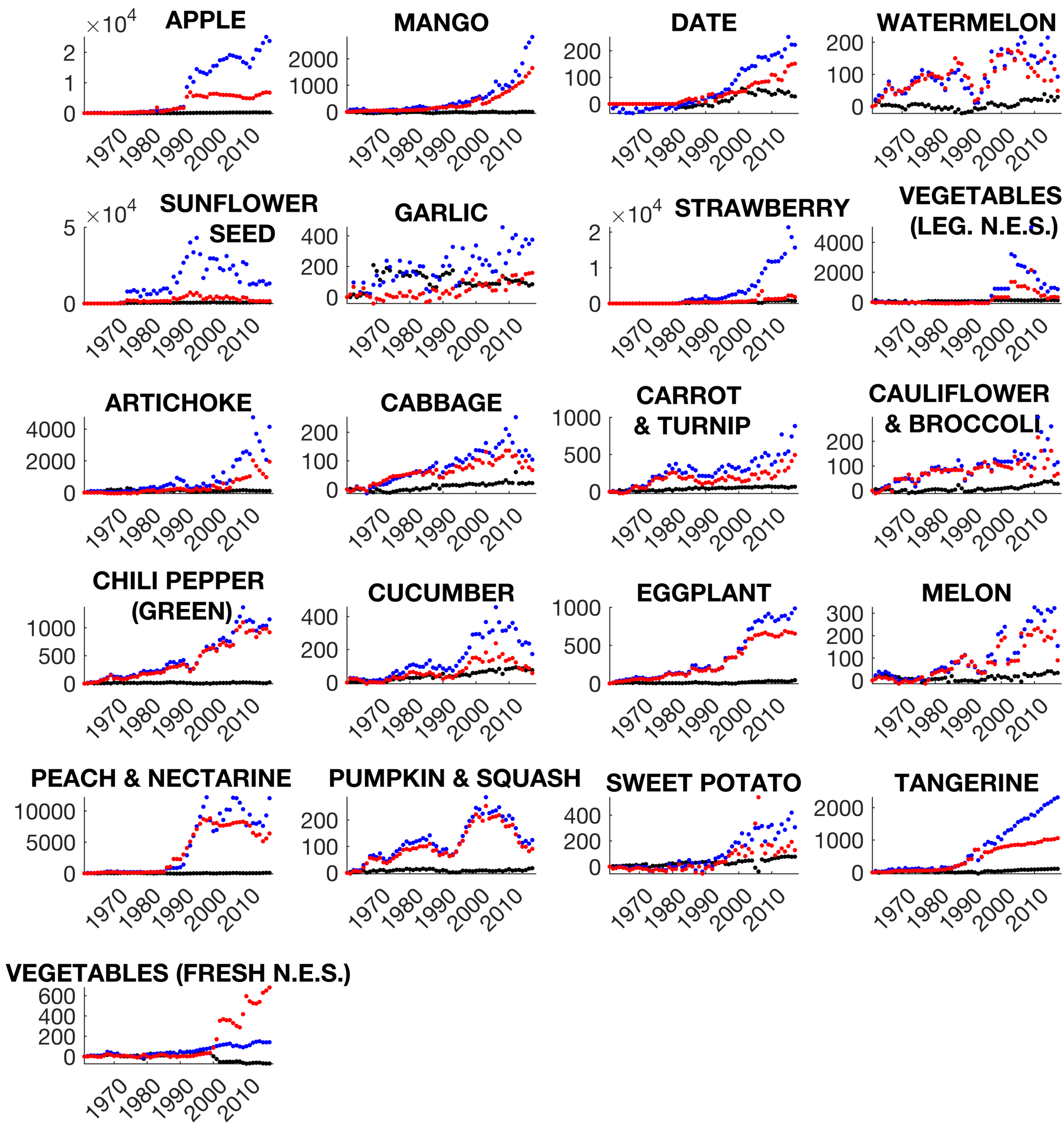
APPLE
MANGO
DATE
WATERMELON
SUNFLOWER SEED
GARLIC
STRAWBERRY
VEGETABLES (LEG. N.E.S.)
ARTICHOKE
CABBAGE
CARROT & TURNIP
CAULIFLOWER & BROCCOLI
CHILI PEPPER (GREEN)
CUCUMBER
EGGPLANT
MELON
PEACH & NECTARINE
PUMPKIN & SQUASH
SWEET POTATO
TANGERINE
VEGETABLES (FRESH N.E.S.)

SUPPLEMENTARY FIG. 3: CROP WATER REQUIREMENTS Water consumption requirements for individual crops used in historical reconstruction (m^3/tonne). The red square indicates the base water consumption value from the literature [31] for the 1996-2005 period. Data Sources: (1) (7) (8)

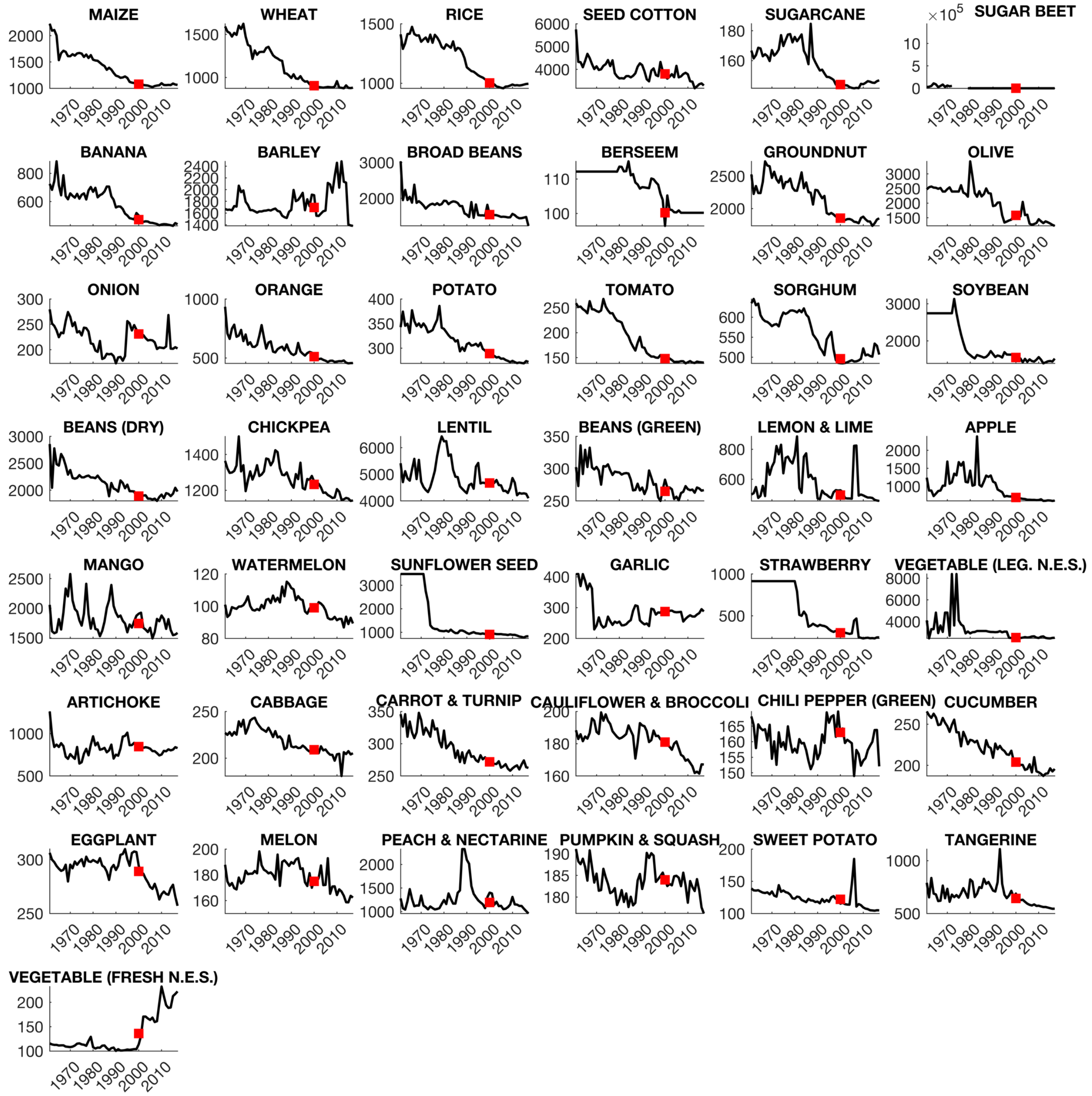

SUPPLEMENTARY FIG. 4: FACTORS IMPACTING CROP WATER REQUIREMENTS (a) Water consumption requirement for major water consuming crops scaled using 1961 irrigation application efficiency (tonnes/m^3). 1961 values for wheat, maize, rice, cotton, berseem, and sugarcane are 0.0006, 0.0004, 0.0007, 0.0002, 0.0043, and 0.006 tonnes/m^3 respectively. (b) Evolving irrigation efficiency scaled water consumption requirement for major water consuming crops (tonnes/m^3). (c) Evolving irrigation efficiency scaled water consumption requirement for major water consuming crops also including proportion of reuse (tonnes/m^3). Data Sources: (1) (7) (8) (16) (18)

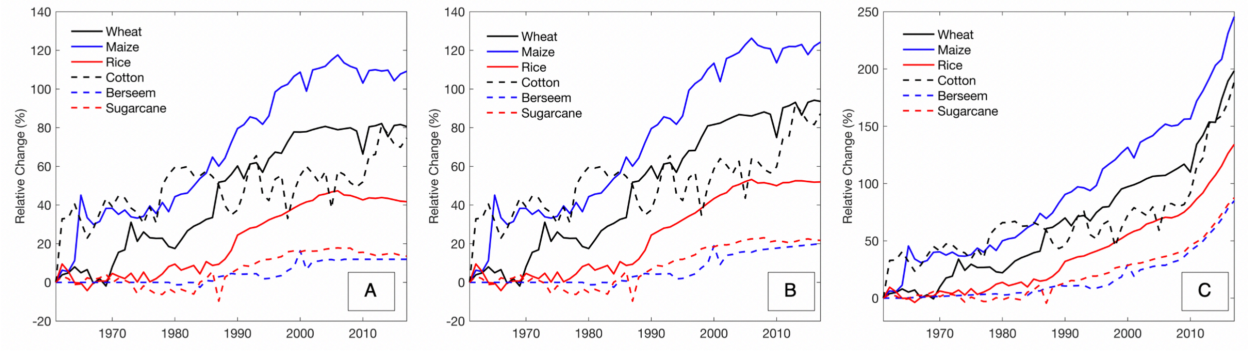

SUPPLEMENTARY FIG. 5: AGRICULTURAL LAND AND IRRIGATION TECHNOLOGY IN EGYPT Development of agricultural area in Egypt and use of irrigation technologies. (a) Old vs. New Land agricultural area (ha) in Egypt using total cropland data (FAO) and details on new land acquisition (Karajeh et al. (2013)) (b) Proportion of land using various irrigation technologies (%). Values are available for 1995, 2000, and 2010 and interpolated between (Data Source: (18)). (c) Estimate of country average irrigation efficiency (%) determined using the proportion of irrigation type in panel b and the FAO stated efficiency of the relative irrigation technologies. (Brouwer et al. (1989))

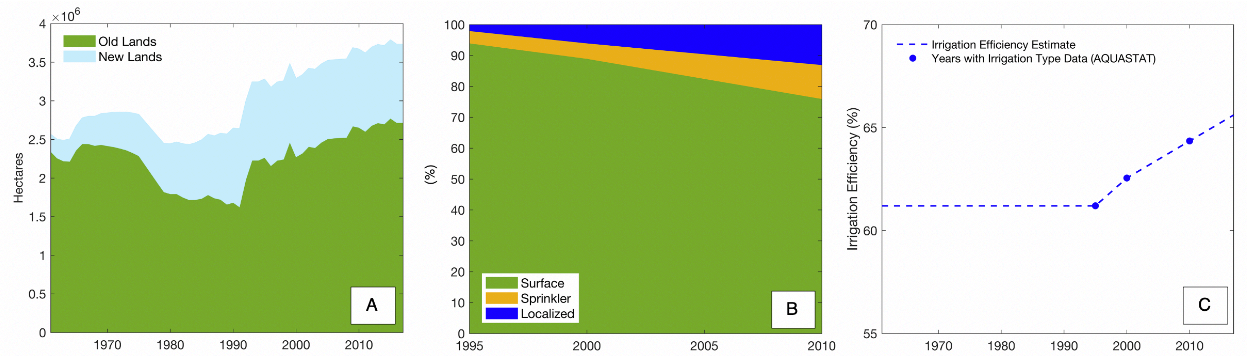

Karajeh, F., Oweis, T., Swelam, A., El-Gindy, A-G., El-Quosy, D. E. D., Khalifa, H., El-Kholy, M., El-Hafez, S. A. Water and Agriculture in Egypt. (Beirut, Lebanon: Food and Agriculture Organization of the United Nations (FAO), 2013) https://hdl.handle.net/20.500.11766/7872

Brouwer, C., Prins, K., & Heibloem, M. Annex 1: Irrigation Efficiencies. In *Irrigation Water Management: Irrigation Scheduling: Training Manual 4.* (1989) http://www.fao.org/3/t7202e/t7202e08.htm

SUPPLEMENTARY FIG. 6: METHODS FOR RECONSTRUCTING AGRICULTURAL WATER USE Historical water use (km^3) for agriculture. Green data show official data for Agricultural use. Error bars represent literature derived estimates of agricultural drainage reuse, groundwater recharge reuse, and wastewater reuse with the assumption that all reuse is applied to agricultural purposes. The lines show bottom-up crop-data based estimates for agricultural use using three different crop water consumption assumptions: 1) constant tonnage water requirement (m^3/tonne) [blue] 2) constant area water requirement (m^3/ha) [red] 3) average of (1) and (2) (m^3/tonne) [black solid]. Data Sources: (1) (2) (7) (8) (16)

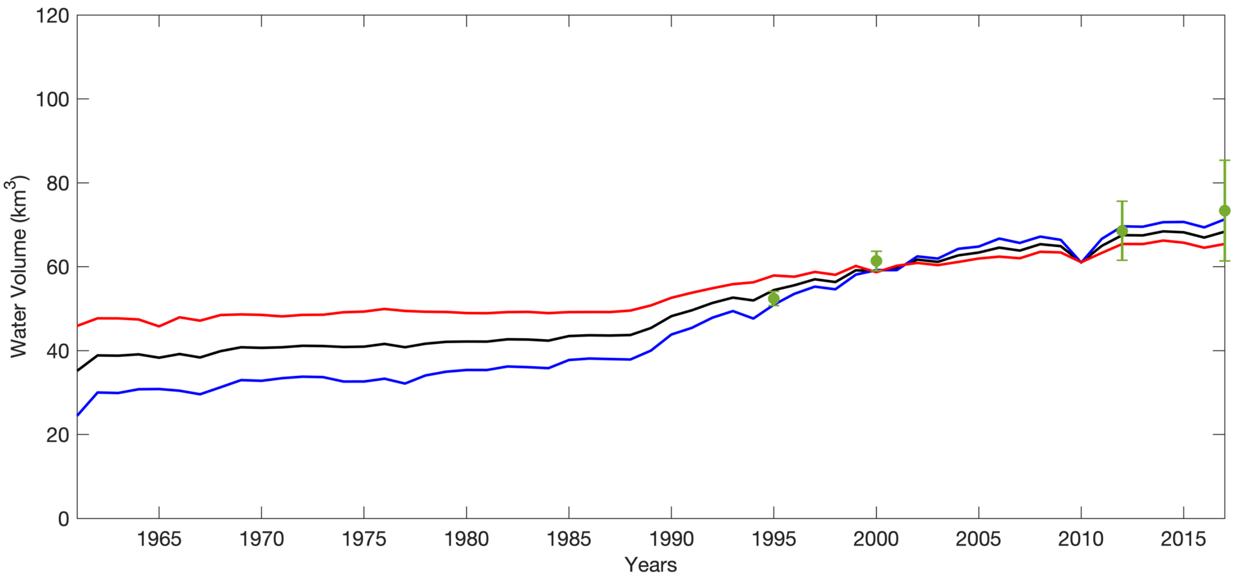

SUPPLEMENTARY FIG. 7: AGRICULTURAL IMPORTS Total import tonnage (million tonnes) and breakdowns of maize, wheat, and soybean imports (million tonnes). Data Source: (11)

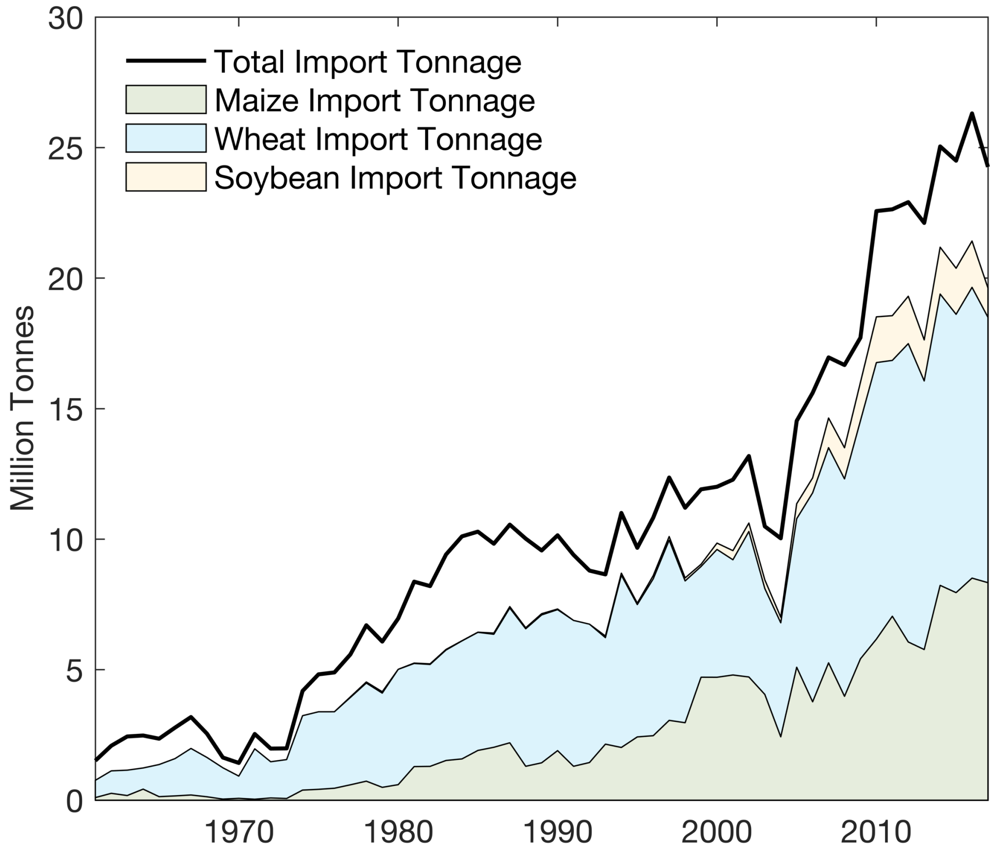

SUPPLEMENTARY FIG. 8: PER CAPITA WATER CONSUMPTION a) Historical per capita water consumption (m^3/capita/year) for agricultural production [black solid] and historical per capita water embodied in primary and secondary crop imports (m^3/capita/year) [black dashed]. Data Source: (1) (2) (4) (7) (8) (11)

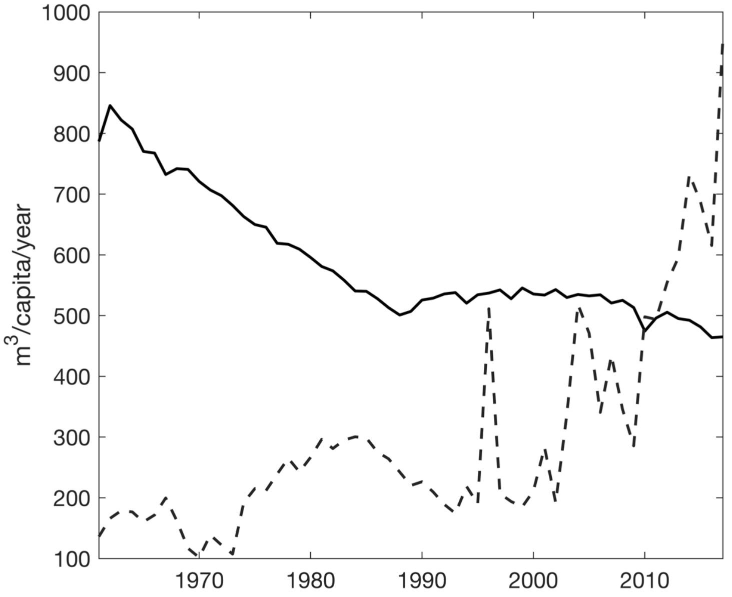

SUPPLEMENTARY FIG. 9: NON-AGRICULTURAL WATER USES AND REUSE
Historical estimates for (a) municipal use (km^3) and industrial use (km^3) and (b) three components of reuse (km^3).

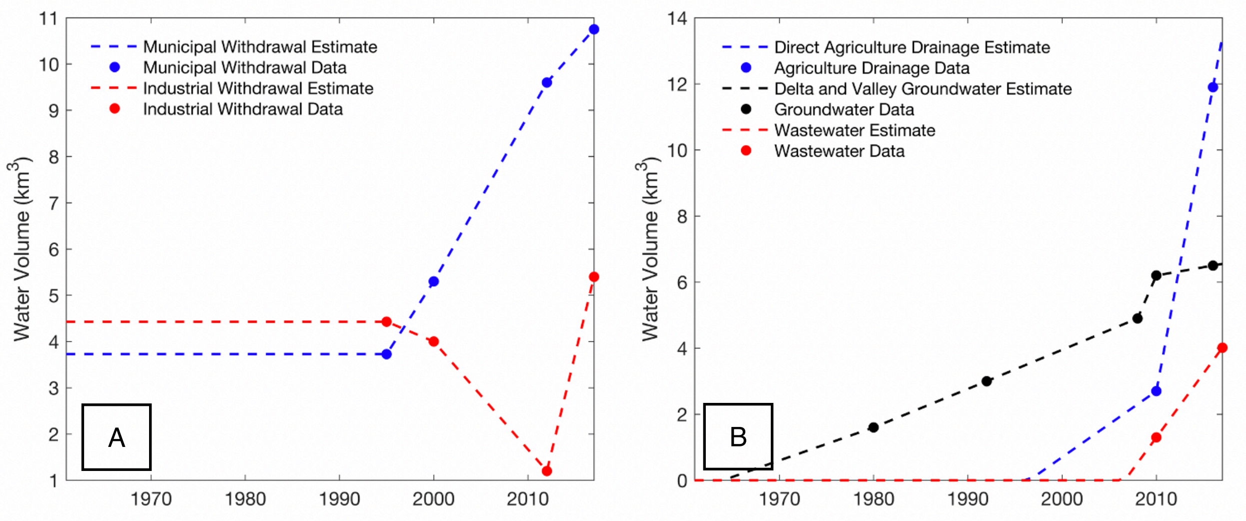

Data Sources:

(A) Municipal – (16)
(A) Industrial – (16)
(B) Direct Agricultural Drainage Reuse: 2010 (16); 2016 (16)
(B) Wastewater Reuse: 2010 (16); 2017 (16)
(B) Delta and Valley Groundwater Reuse: 1980 (Sallam et al., 2018); 1992 (Survey/Morsy); 2008 (Survey/Morsy); 2010 (Mahmoud et al., 2017); 2016 (AQUASTAT CFS)

SUPPLEMENTARY FIG. 10: FEED AND ANIMAL PRODUCTS (a) Feed products in Egypt (tonnes) (b) Estimates of water consumption (km^3) associated with feed products in panel (a) vs. consumption associated with animal product production in Egypt (meat, milk, and eggs) (c) Total virtual water import (km^3) (consumption equivalent) associated with animal products [black] and historical per capita water embodied in animal product imports (m^3/capita/year) [black dashed]. Data Source: (1) (3) (4) (7) (8) (9) (10) (11)

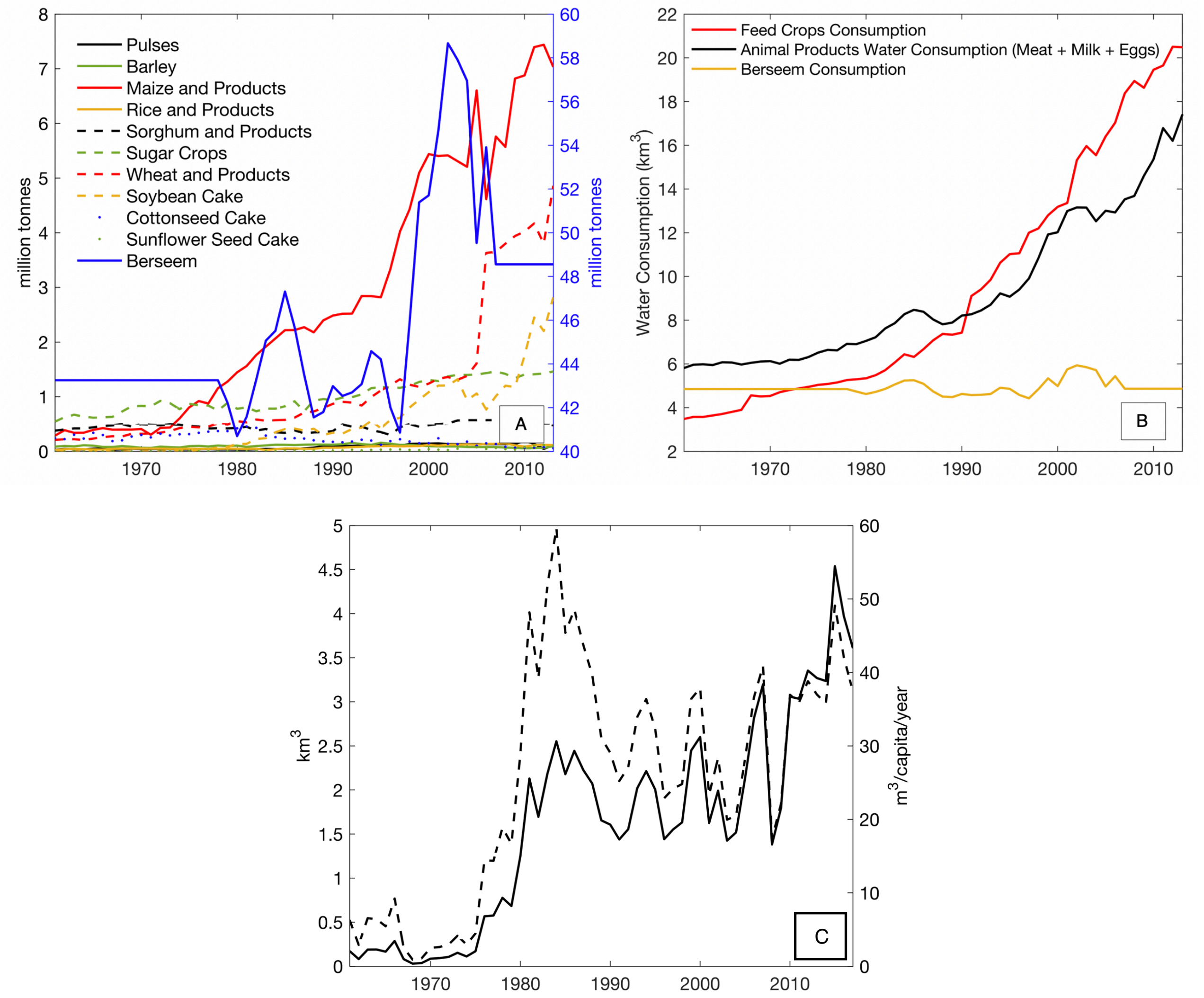

SUPPLEMENTARY FIG. 11: PER CAPITA GDP AND POPULATION SCENARIOS (a) High and low projections of GDP per capita growth (%) used in the study (black) along with the projection that follows the past 30-yr average growth (red) used as the nominal scenario. (b) High and low projections of population used in the study (black) alongside the high, medium, and low variant UN population projections (blue). Also plotted is the projection that follows the UN Medium projection scenario (red) and is used as the nominal scenario. Data Source: (6) (4)

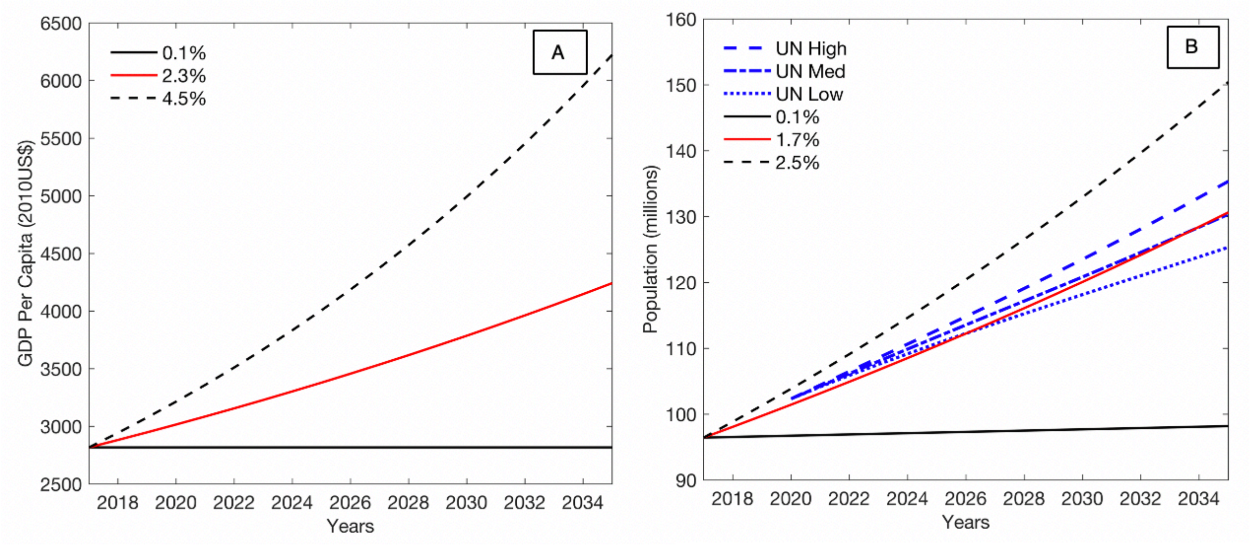

SUPPLEMENTARY FIG. 12: MUNICIPAL WATER USE PROJECTIONS Municipal water use projections (km^3) based on population projections in Supplementary Fig. 11b. Data Source: (4) (16)

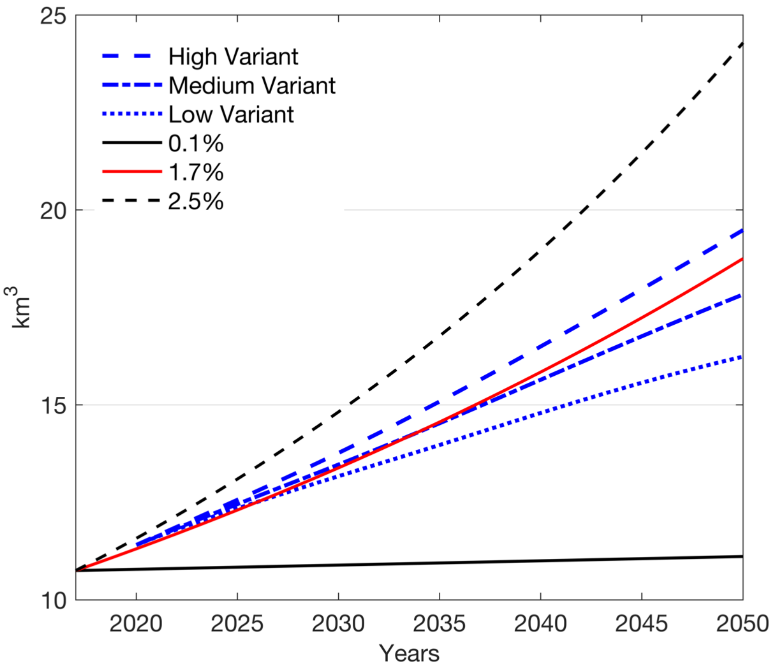

SUPPLEMENTARY FIG. 13: FUTURE DEMAND TONNAGE Future additional demand (million tonnes) in a given year vs. 2017 for primary crops and cotton lint bounded by two scenarios: 4.5% GDP per capita growth and 2.5% population growth [black] and 0% GDP per capita growth and 0.1% population growth [blue]. The nominal growth scenario (2.3% GDP per capita growth and 1.7% population growth is shown in red. Data Source: (1) (2) (4) (6) (7) (8) (11)

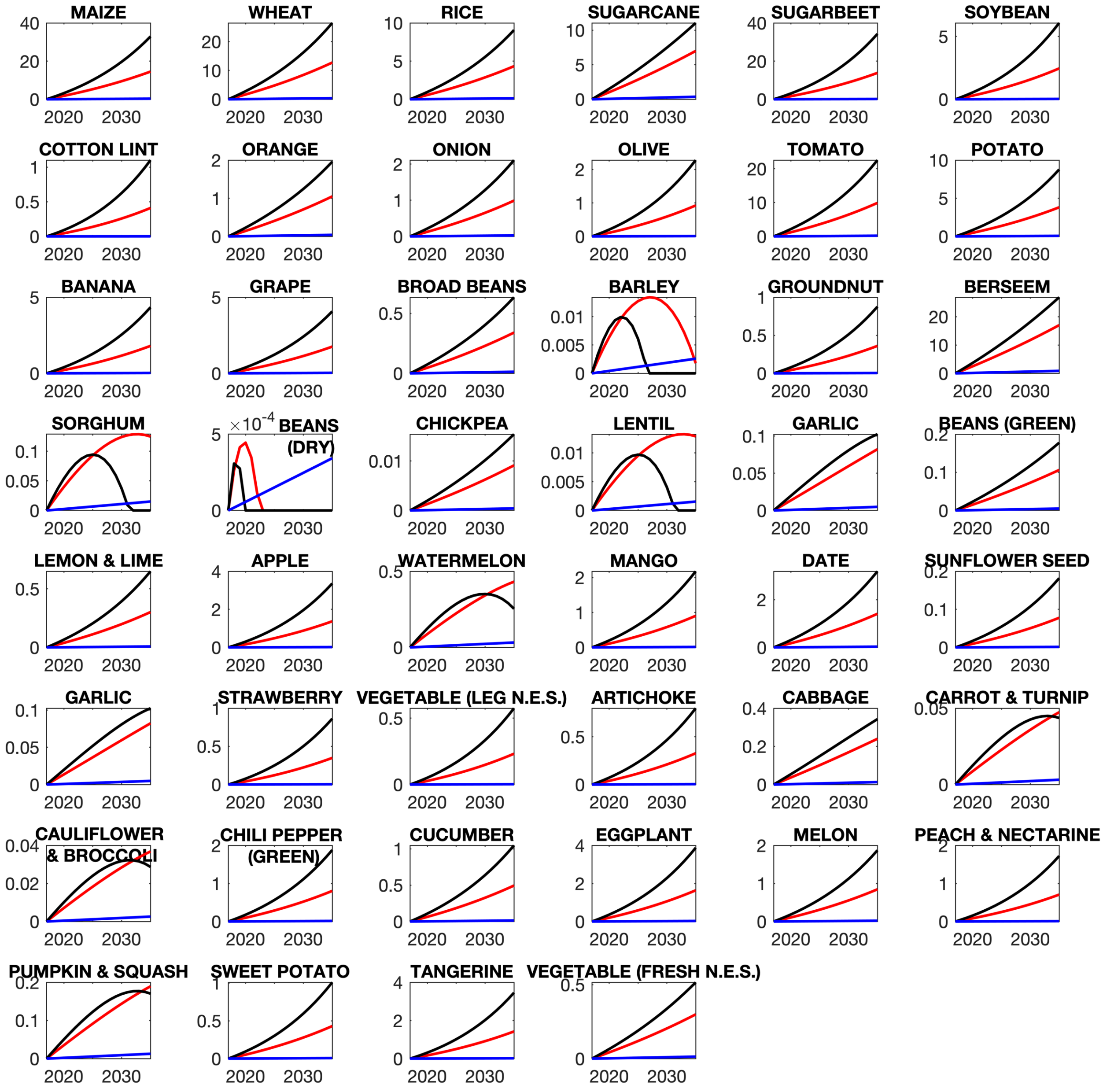

SUPPLEMENTARY FIG. 14: CEREAL IMPORT DEPENDENCY a) Cereal Import Dependency Ratio (%) using FAO data (2000-2017) and projected data for maize, wheat, rice, barley, and sorghum in the nominal scenario (2.3% GDP per capita growth and 1.7% population growth) (2017-2035) [black]. This is compared to the historical ratio for Japan [blue] and S. Korea [red]. (b) 2003-2012 Avg. Cereal Import Dependency Ratio vs. 2003-2012 Avg. GDP per capita. African countries [red] and other select countries important for import and export relationships [black], Projected ratio for Egypt [blue x] in the nominal scenario (2018-2035). Data Source: (6) (12)

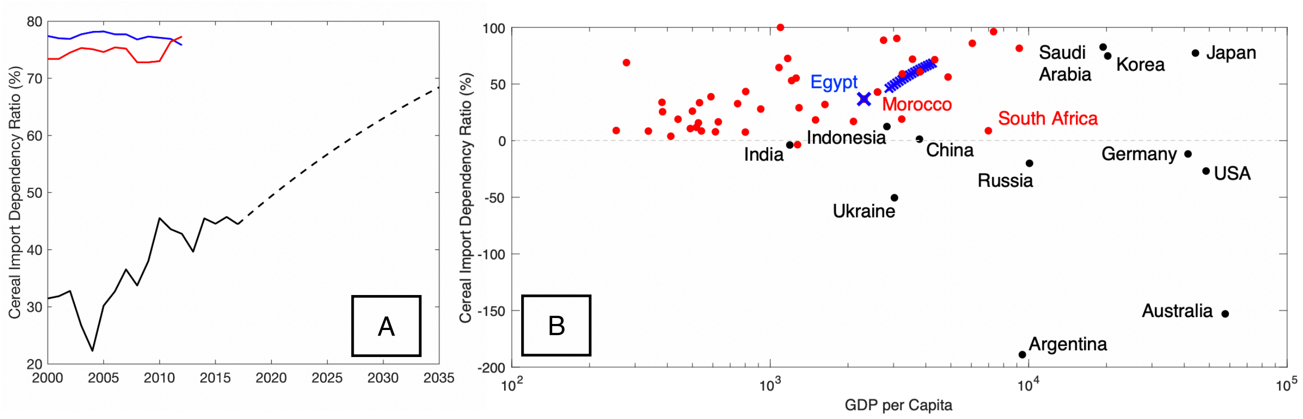

SUPPLEMENTARY FIG. 15: REUSE AND EFFICIENCY (a) Volume of water withdrawn for agriculture but not used directly for crop evapotranspiration [black] and amount of reuse (treated municipal wastewater, direct use of agricultural drainage, use of local groundwater recharge) [red]. Note that the reuse exceeds the water surplus because a small amount of reuse is initially withdrawn for municipal purposes (treated municipal wastewater). (b) a measure of the true system efficiency by using net losses (application losses minus reuse).

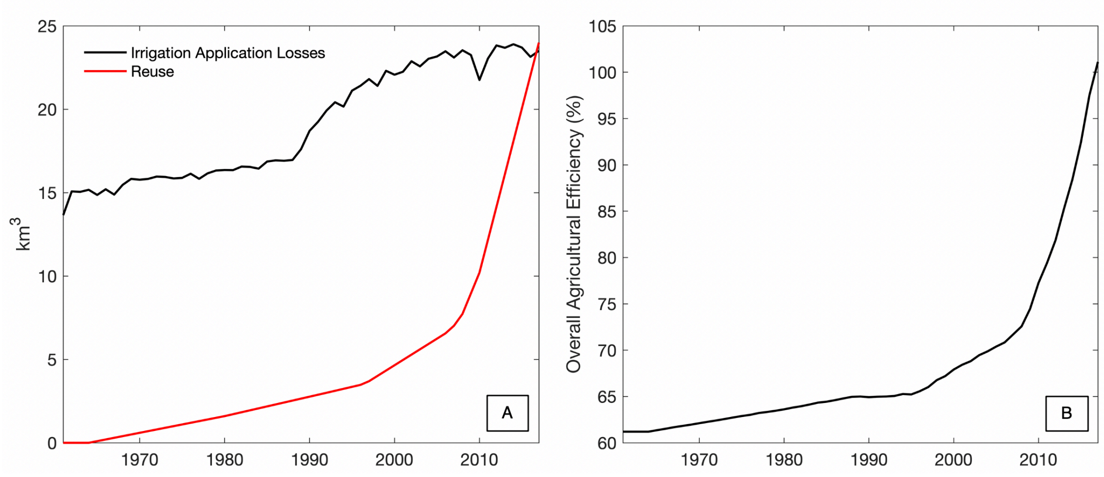

SUPPLEMENTARY FIG. 16: 1988-2017 EGYPTIAN NILE WATER SYSTEM FLUXES

Egyptian Nile Water System 1988-2017 average annual fluxes (km^3/yr). Red values are sinks, blue are sources, and purple indicates reuse. See Figure 4 for Data Sources.

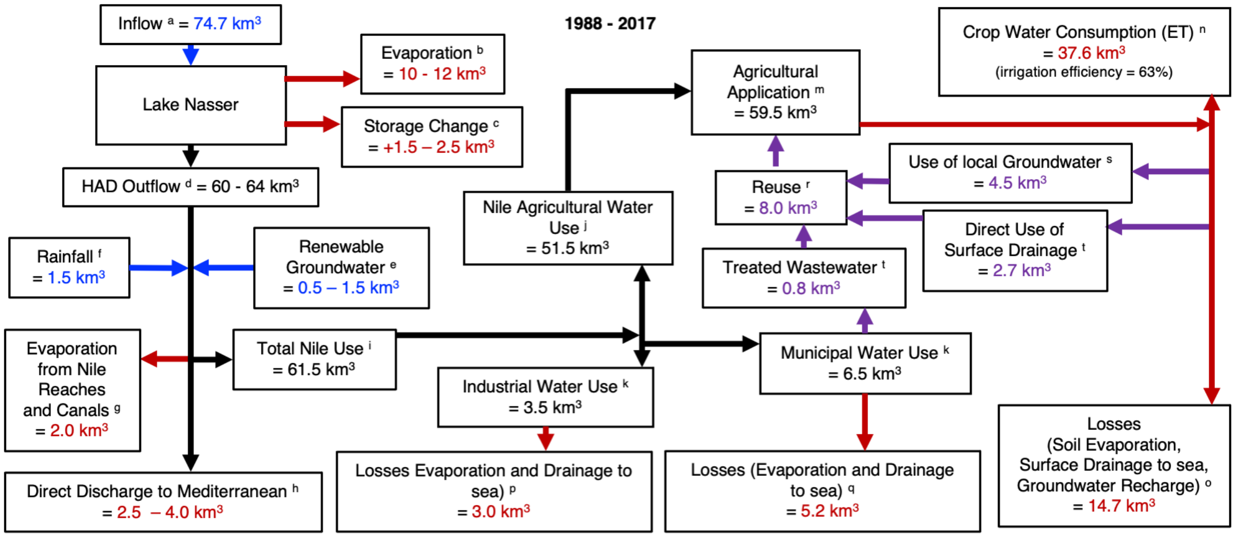

SUPPLEMENTARY REFERENCES

[1] Mekonnen, M. M. & Hoekstra, A. Y. The green, blue and grey water footprint of crops and derived crop products. *Value of Water Research Report Series* No. **47**, Vol. I, (UNESCO-IHE, Delft, the Netherlands, 2010). https://research.utwente.nl/en/publications/the-green-blue-and-grey-water-footprint-of-crops-and-derived-crop-3

[2] Global Water Intelligence. Egypt to slash subsidies in water and wastewater https://www.globalwaterintel.com/news/2017/31/egypt-to-slash-subsidies-in-water-and-wastewater?dm_i=36G3,IGWK,2MX9NG,1XJV0,1

[3] Cornish, G., Bosworth, B., Perry, C., & Burke, J. Annex 1: Water Charging for Irrigation – data from the literature. In *Water Charging in Irrigated Agriculture: An analysis of international experience* (2004). http://www.fao.org/3/y5690e/y5690e0b.htm

[4] Lixia H., Wallace E. T., Rachid D. & Gamal S.. Policy Options to Improve Water Allocation Efficiency: Analysis on Egypt and Morocco. *Water International*, **31**, 3 (2006) http://doi.org/10.1080/02508060608691935

[5] Hopwood, D, Goldschmidt, A. E. et al., Egypt: Agriculture and Fishing, In *The Encyclopædia Britannica*. Published December 15, 2020 https://www.britannica.com/place/Egypt. [Accessed January 11, 2021]

[6] Mekonnen, M.M. & Hoekstra, A.Y. The green, blue and grey water footprint of crops and derived crop products. Value of Water Research Report Series No. 47, Vol. II, App. II. (UNESCO-IHE, Delft, the Netherlands, 2010). https://research.utwente.nl/files/59480760/Report47_WaterFootprintCrops_Vol2.pdf

[7] Omar, M. E. D. M, & Moussa, A. M. A. Water management in Egypt for facing the future challenges, *Journal of Advanced Research*. **7**, 3 (2016) https://doi.org/10.1016/j.jare.2016.02.005.

[8] Takouleu, J. M.. EGYPT: Lining irrigation canals to save 5 billion m^3 of water. Afrik21 (2020). Available Online at https://www.afrik21.africa/en/egypt-lining-irrigation-canals-to-save-5-billion-m%C2%B3-of-water/

[9] Grafton, R.Q., Williams, J., Perry, C. J., et al. The paradox of irrigation efficiency. *Science*. **361**, 6404 (2018). http:/doi.org/10.1126/science.aat9314

[10] United Nations, Department of Economic and Social Affairs, Population Division. Total fertility by region, subregion and country, 1950-2100 (live births per woman). World Population Prospects 2019, Online Edition. Rev. 1. (File FERT/4). (2019) https://population.un.org/wpp/Download/Files/1_Indicators%20(Standard)/EXCEL_FILES/2_Fertility/WPP2019_FERT_F04_TOTAL_FERTILITY.xlsx

[11] Hassan, E.. Egypt to plant 100 million olive trees across the country by 2022. Aldiplomasy (2019). Available Online at https://www.aldiplomasy.com/en/?p=17158;

[12] Nassar, A. Comparison of micro irrigation systems for olive trees. *Misr J. Ag. Eng*., **26**, 1 (2009)

[13] Antonelli, M. & Tamea, S. Food-water security and virtual water trade in the Middle East and North Africa. *International Journal of Water Resources Development*, **31**, 3 (2015) http://doi.org/10.1080/07900627.2015.1030496

[14] World Bank. Employment in Agriculture (%) of total Employment. (modeled ILO estimate) – Egypt, Arab Rep. Data from International Labour Organization, ILOSTAT database. (2020) [Retrieved January 11, 2021]. Available Online at https://data.worldbank.org/indicator/SL.AGR.EMPL.ZS?locations=EG

[15] Food and Agriculture Organization of the United Nations (FAO). Egypt Country Programming Framework (2013). Available Online at http://www.fao.org/publications/card/en/c/0603d9fd-85c9-4557-8772-eb40602666a6

[16] Gaulier, G. & Zignago, S..”BACI: International Trade Database at the Product-Level. The 1994-2007 Version," CEPII Working Paper 2010-23, October 2010, CEPII.

[17] RFE/RL. Russia Cuts Off Wheat, Other Grain Exports. Radio Free Europe/Radio Liberty (2020). Available Online at https://www.rferl.org/a/russia-cuts-off-wheat-other-grain-exports/30577633.html

[18] Zeitoun, M., Allan, J. A., & Mohieldeen, Y. Virtual water 'flows' of the Nile Basin, 1998–2004: A first approximation and implications for water security. *Global Environmental Change*. **20**, 2 (2010). http://doi.org/10.1016/j.gloenvcha.2009.11.003

[19] Food and Agriculture Organization of the United Nations (FAO). AQUASTAT Country Profile – Sudan. (Rome, Italy, 2016). Available Online at http://www.fao.org/3/i9808en/I9808EN.pdf

[20] Dost, M., Bimal, M., El-Nahrawy, M., Khan, S., & Serkan, A. Egyptian Clover (Trifolium alexandrinum). King of Forage Crops. (Food and Agriculture Organization of the United Nations, Regional Office for the Near East and North Africa, Cairo, 2014) http://www.fao.org/3/a-i3500e.pdf
