# Supplementary material for: Past and future trends of Egypt’s water consumption and its sources
Source: Nat Commun. 2021 Jul 23;12:4508. doi: 10.1038/s41467-021-24747-9 (PMC8302683; doi:10.1038/s41467-021-24747-9)
Supplement: Supplementary file 3 — Description of Additional Supplementary Files [file 41467_2021_24747_MOESM3_ESM.pdf]

Description of Additional Supplementary Files

File name: Supplementary Data 1
Description: Tab 1: Supplementary Table 3: CROP AND ANIMAL PRODUCT DATA Key crop and animal product figures and water usage estimates (2007-2011 average). * Indicates commodities where the 1996- 2005 water consumption value was used in absence of yield and harvested area data for scaling. All total water use estimates are given in terms of consumption (without irrigation efficiency scaling). Import and Export Price/Quantity are computed individually for all years with official data and then averaged over the period.

File name: Supplementary Movie 1
Description: This movie uses data to show the increases in population in Egypt, and satellite imagery to show the development of Cairo, and the expansion of agricultural areas at the edges of the Nile Delta.
