# Supplementary material for: Past and future trends of Egypt’s water consumption and its sources
Source: Nat Commun. 2021 Jul 23;12:4508. doi: 10.1038/s41467-021-24747-9 (PMC8302683; doi:10.1038/s41467-021-24747-9)
Supplement: Supplementary file 2 — Peer Review File [file 41467_2021_24747_MOESM2_ESM.pdf]

REVIEWER COMMENTS

Reviewer #1 (Remarks to the Author):

The article provides an important contribution to the literature, particularly in our understanding of Egypt's increasing dependency on imported food as 'virtual water' in addition to depending on the Nile. The authors do a good job with this assertion, and an excellent job elucidating a new water balance for Egypt. The article however has several items that are problematic. Foremost is a structural issue where the authors introduced new analyses throughout the discussion that were not described in the results. In this article, the Results sets up the problem and the Discussion proposes the solutions, but the solutions required a significant amount of analyses. My feeling is that the authors are trying to pack in too much into this paper. My recommendation is to restructure this paper in more succinct way that fits more of the analysis into the results, or perhaps references portions of the work published elsewhere (either the establishment of the problem or the solutions). I understand the challenges this author is facing here of too much information, but it negatively affects the readability of the work.

Detailed comments are below after each specific quotation:

15: "Egypt is likely to import more virtual water than is supplied by the Nile, bringing into question the historical characterization of Egypt as “the gift of the Nile”."
This framing makes for an interesting read and talking points, but its value is uncertain. Egypt will be dependent on both the Nile and imports for the foreseeable future, with that balance shifting. The discussion should address the implications of this shift of perception vis-à-vis basin wide development.

29: “The current policies on water productivity, water reuse, and population control will not be enough to close the demand gap in the future.”
A citation should be provided to justify this statement, or the authors should state that their analysis demonstrates this.

31: “Externally, the conflict with the Nile Basin countries over water allocation and storage which focuses on construction of the Grand Ethiopian Renaissance Dam (GERD) and the sustainability of the allocations to Egypt adds further strains [2].”
Poorly worded sentence. The author should not use the term ‘allocations’ unless they explain that this volume is only agreed between Egypt and Sudan.

46-47: “Second, Egypt passed the carrying capacity of the Nile in the 1980’s and was importing the use
equivalent of at least 40 km3 of virtual water in the late 2010’s [3]. Assuming persistence of the recent socioeconomic trends, we project that Egypt will likely reach an import of 61.5 km3 during this decade of the 2020s.”
This quotes an import of 40 km3 in the late 2010’s, but only gives a reference of a book from 1997. It needs to be clearer where this number came from.

55-61: “In order to manage this process in a sustainable fashion, we recommend adoption of a hybrid

national policy consisting of both Water Value Appreciation (WVA): a robust water pricing system leading to more efficient cropping patterns and enhanced irrigation application efficiency, as well as Water Share Amplification (WSA): an approach of smart and efficient management of agricultural export and import (virtual water), applicable to arid countries in general, that amplifies Egypt's share of natural water resources."
Are there citations to these methods (WVA and WSA) or are these newly introduced here?

70 "Egypt's millennia long existence…"
This makes it sound like Egypt has only existed for 1000 years. Please reword.

76: "practically the only water resource"
Can the author quantify this percentage dependency on the Nile?

79-82: Records of flood heights from the Rhoda Nileometer show these levels remained relatively constant over the 800-year record [5] [6]. The flood heights over this period are comparable. Research has suggested "very little downcutting in Nubia since [the time of the New Kingdom some 3000 years ago]" [7].
These sentences are repetitive. No context for what "downcutting in Nubia" is supposed to mean.

83: "Dongola have been slightly decreasing, as a result of increased withdrawal of natural flows upstream [8] [9]."
This should be quantified and backed up with data.

Figure 1a. On the plot, please provide a brief explanation/mention of missing data from 1400s until 1800s. Label "Discharge from Aswan Dam" and "Flow at Dongola" to clarify that these are mixed unit plots.

95: What is "CUT" statement?

99: Reference 13 is unstable.

107: "The rapid changes in Egypt's water demand have spurred equally large responses…"
Rapid and large are not synonymous. Please reword.

109-112: "… an attempt to control and manage water supply and demand. Egypt's efforts (documented in Figure 2) have been substantial in five areas"
In the introduction there were 4 "avenues" mentioned and did not include the attempt to control population growth. These two lists should align, possibly by adding this element to the introduction list.

Figure 2. The inconsistency in date ranges should be remedied.

Figure 2a: This is confusing to me. First, it looks like the annual flows at Aswan dropped from an average between 80 to 100 bcm, to around 50 bcm. Are these only a result of Evaporation, which is estimated to be 10-12 bcm. Perhaps Sudanese withdrawals? The author needs to quantitatively explain the difference. The data sources for this is from 1998. The authors should extend this to include the most recent years. Also, the storage dots do not say much. This would be better if it

showed the increasing storage in the entire basin up until the recent time (Roseries, Sennar, Merowe, Khashm El Girba etc.).

Figure 2e: What is the data source for Virtual Water imports?

120-121: “M.M. Mekonnen and A.Y. Hoekstra” – Names are not needed. It should be sufficient to show reference numbers.

Figure 3. The total volume from of water use in Figure 3a is somewhat different than 3b, which should always be higher since it combines municipal and industrial. What is the data source of the use line in Figure 3b? Please make figures clear that this only for Egypt.

125: “Egypt began fully utilizing available water resources in the 1980’s and has only met increasing agricultural water demand through virtual water imports (dark grey shading) and increasing reuse (light red shading).”
This makes no mention of the increasing uses is Sudan, which is a highly debated subject, particularly the volume of current upstream depletions. The wording of “available water resources” allows this statement to be technically accurate, however there should at minimum be a mention that these available water resources include all water not consumed upstream, which is dynamic. Bringing in the topic of ‘basin closure’ would also be worthwhile here.

Falkenmark, M., Fraiture, C.d. and Vick, M.J. (2009), Global change in four semi-arid transnational river basins: Analysis of institutional water sharing preparedness. Natural Resources Forum, 33: 310-319. doi:10.1111/j.1477-8947.2009.01254.x

132: “we show that Egypt’s direct consumption of the Nile is roughly 61.5 km3 on average from 1988 to 2017.”
Does the 61.5 bcm relate to the data on Figure 3a or 3b?

132-135: “Adding the environmental flow to the Mediterranean of about 2-4 km3 [26], Egypt consumes 8.0 to 10.0 km3 more than the share of 55.5 km3 allocated through the 1959 Nile Agreement between Egypt and Sudan.”
I believe the author is considering the flows to the sea as a consumption. Perhaps “releases” is a better word than consumption. This should be backed up with any available data.

135: “This additional water comes partially from Sudan’s unutilized share of 4-5 km3 [27], and partially from increases in the Nile flow of about 5-6 km3 [28] [29].”
The author should specify that this share is relative to the 1959 agreement, which is not recognized by other riparians. The authors should also recognize the uncertainty of the current Sudanese depletions. The link to the NBI source is not working.

138-140: “This schematic relies on several independent sources of data, each flux is uncertain, and hence does necessarily satisfy strict water balance.”
These schematics are a major contribution, that will get a lot of attention. However, I’m concerned that they do not satisfy a water balance and the sources are not sufficiently documented.

Figure 4: Are the storage changes positive or negative?

Figure 4b: Mass balance for 2010-2017: Total Nile Use does not fall in the possible ranges provided. How can the total Nile use be 62.3 when the water available (HAD outflows (60.1)+rainfall (1.5)+ max GW (1.5)) = 63.1 bcm and the losses (Reach and Canal evap (2.0) + min Med Sea discharge (2.5)) are 4.5 bcm, leaving only 58.6 bcm to divert? I understand that it is impossible to get exact numbers, but the ranges provided should allow a possible mass balance. Overall, I'm concerned that the lack of mass balance will lead to confusion. Please review all the numbers in Figure 4 carefully and provide a citation for each in the supplementary materials so the reader can understand how they are derived.

Figure 5: What does (use eq.) mean in the caption? On Figure 5b, what are the units of the numbers on the dashed lines of the plot (bcm I presume).

142 and Figure 6: The authors must be explicit of their assumptions on further upstream development in Sudan and elsewhere.

182-190: "We recommend water demand management through the lens of Water Value Appreciation (WVA) including: (1) implementing new water pricing strategy, (2) improving application efficiency at the field scale, and (3) adjusting cropping patterns to emphasize ecological suitability and water use efficiency. In addition, we recommend further changes that are guided by a Water Share Amplification (WSA) which focuses on (1) amplifying virtual water share in the global food market, (2) increasing cooperation with Nile basin neighbors to import animals and animal products (meat) instead of using valuable water in producing them locally, and (3) bringing fertility measures in line with the rest of the world to control population growth and amplify per capita water share."
It seems strange that the authors are introducing these topics in the discussion, yet they have not been analyzed in the results. WVA and WSA are described in the methods and supplementary materials, but they since they seem to be central to the arguments, they should be considered results that are reflected upon in the discussion.

200-202: "There is therefore an argument to implement water pricing for farmers and tie it to either the marginal cost of increasing water supplies or to the cost of increasing water use efficiency (though upgrading or maintaining delivery systems and irrigation methods) [32]."
Use of passive voice is problematic here because it masks if you are advocating this or whether you are simply pointing out that He et al (2006) is suggesting this.

204: "Egypt dramatically increased its crop yields through the use of more water and the application of agricultural technologies such as fertilizer."
The author should quantify this by referring to the results or other literature.

207-208: "Water productivity (tonnes/m3) did not improve as drastically until the more recent aggressive perusal of reuse technologies."
The author should cite evidence in their analysis or other sources if possible.

215: "Reducing conveyance and application losses are key in increasing water"
While true in principle, the governance challenges to making efficiency improvements. The authors should note these obstacles.
Grafton, R. Q., Williams, J., Perry, C. J., Molle, F., Ringler, C., Steduto, P., . . . Allen, R. G. (2018). The paradox of irrigation efficiency. Science, 361(6404),

748-750. doi:10.1126/science.aat9314

218: “There is a limit to this efficiency increase, as more efficient application reduces the amount of available reuse.”
Is this itself a limitation or simply an implication of becoming more efficient?

221-222: “their amount of reuse equaled the excess with drawn that is not used in direct plant evapotranspiration”
What is the value of this comparison?

226-230: “Subsequently, selection of crops that are ecologically suited and easily irrigated using high efficiency measures should be a key goal for future agricultural policy. For example, the expansion of olives is an option as a crop that is well suited to the regional climate and to the use of drip irrigation methods. This expansion is already planned for Egypt, and their efficiency under drip irrigation suggests a promising role in Egypt’s export portfolio [37] [38].”
To what extent (quantitatively) can efficiency improve the situation? Without numbers, this becomes an obvious assertion.

234: “Egypt needs to strive further to reduce fertility rates and slow population growth.”
Yes, this an important issue, but why is this included in Water Share Amplification?

253: “Water Share Amplification (WSA) is introduced here as a concept of maximizing the water available to a country, both in terms of water productivity and in terms of market value.”
Introducing an analytical method into the discussion? Why wasn’t this presented in the results? Is this a new method?

261-263: "Using a set of water share amplification metrics calculated for current crops produced and traded we develop a WSA proposal presented in Supplementary Table 5."
The author is still presenting new analysis and solutions even at the end of the discussion. Seems like they are trying to pack in too much.

297-300: “Egypt will need to compensate for roughly 15-20 km3 of losses over the next several years (4.9 km3 and 13.5 km3 of filling related losses in the first and second years respectively) from the filling of the Grand Ethiopian Renaissance Dam (GERD) [44].”
The notion of a loss due to the GERD that needs to be compensated during the filling is highly dependent on the hydrologic conditions during this period, management practices, agreements between the countries, and what constitutes a loss. For example, it is unlikely that the first-year filling had any negative implications with respect to water shortages, and a subsequent hear in 2021 might result in the same. The focus of this article should remain on the long-term supply-demand imbalances and the authors should not casually analyze the transient implications of the GERD. I also note the authors have acknowledged in previous publications that increased storage may have positive implications for Egypt as well. Referencing news articles (with broken links especially) on this topic is not appropriate when much peer-reviewed literature exists on this topic.

303: “Demand and supply of water will also be affected by climate change.”
A mention of climate change is appropriate here because of the author knowledge, citations used, and the potential implications on the supply-demand imbalance, but the paper should explicitly

mention that these elements are not analyzed here.

The discussion should expound on the results and the conclusions should summarize the research findings convincingly rather than introduce new results.

Reviewer #2 (Remarks to the Author):

This paper makes the indisputable argument that Egypt will need to address existing, and increasing, water security through reducing demand (e.g. through reducing per capita consumption of water, reducing agricultural/municipal/industrial water losses, and relying more on virtual water imports).

Some suggestions for the authors to consider:

* Including discussion of the potential impacts of sea level rise on Nile delta agriculture earlier in the paper (in now only comes up in the conclusion) as an added dimension to water insecurity.

* Change the language around (or clarify) "aggressive population control". The language, along with the reference to Asia's dramatic population decrease, seem to imply that the author's are encouraging the use of the one child policy in Egypt. I find this discussion to be the most problematic part of the suggested recommendations and seems like it was inserted last minute.

* The paper mentions Egypt's reliance on the unused portion of Sudan's water allocation under the 1959 Nile Agreement. 1-2 sentences on how regularization of the flow of the Nile downstream of the GERD may increase Sudan's water use for agriculture (there are several papers on this) would strengthen this discussion.

* I don't agree with the assertion that Egypt importing more virtual water challenges its historical characterization of Egypt as "the gift of the Nile", and I worry that the title will distract people from the theme of the paper and that you may actually lose your target audience (i.e. Egyptian water policymakers). [But that's a stylistic difference in opinion.]

* Figure 4 is a really interesting - I haven't seen such a clear schematic on water use in Egypt and this by itself is an exciting contribution. I can't comment on the accuracy of the numbers, but assuming they are accurate, it is a very rich figure, but you should explicitly include the sources for these numbers (and how they're calculated).

* It may be outside the purview of this paper, but some discussion of which policies may be more politically feasible than others would be valuable.

* Some reference to Zeitoun et al's (2009) work on virtual water trade in the Nile basin could strengthen the paper, especially in its discussion of increasing cooperation with Nile basin neighbors to import animals and animal products.

I've included additional comments on the pdf but most the rest of the comments are minor.

- Yasmin Zaerpoor

To the reviewers of manuscript NCOMMS-20-38940A,

We thank you for your careful reading of our manuscript originally titled *When Egypt is No Longer the "Gift of the Nile"* and your valuable comments on how it can be improved. We have provided detailed point-by-point responses to all of your comments that include a detailed description of changes made. All comments are numbered C# by reviewer and answers are marked A# accordingly. All changes can be seen in the revised manuscript as well, marked in red, and the line numbers and references that are given refer to the revised manuscript. We hope that our revisions to the manuscript satisfy any concerns you may have had and thank you again for your respected input.

Sincerely,
Catherine A. Nikiel and Elfatih A. B. Eltahir

Reviewer 1

General Comments

C1: The article provides an important contribution to the literature, particularly in our understanding of Egypt's increasing dependency on imported food as 'virtual water' in addition to depending on the Nile. The authors do a good job with this assertion, and an excellent job elucidating a new water balance for Egypt. The article however has several items that are problematic. Foremost is a structural issue where the authors introduced new analyses throughout the discussion that were not described in the results. In this article, the Results sets up the problem and the Discussion proposes the solutions, but the solutions required a significant amount of analyses. My feeling is that the authors are trying to pack in too much into this paper. My recommendation is to restructure this paper in more succinct way that fits more of the analysis into the results, or perhaps references portions of the work published elsewhere (either the establishment of the problem or the solutions). I understand the challenges this author is facing here of too much information, but it negatively affects the readability of the work.

A1: Thank you for your careful reading of the manuscript and taking the time to suggest changes to the structure. In accordance with your comments, we have restructured some portions of the Results and Discussion to ensure that we do not present new information too late in the manuscript. In part this involves moving some analysis completely to the supplementary material, as it may be of interest to readers, but its absence in the main article does not weaken the conclusions. We describe these moves in further detail in the answers below.

Specific Comments

C2: Line 15: "Egypt is likely to import more virtual water than is supplied by the Nile, bringing into question the historical characterization of Egypt as “the gift of the Nile”." This framing makes for an interesting read and talking points, but its value is uncertain. Egypt will be dependent on both the Nile and imports for the foreseeable future, with that balance shifting. The discussion should address the implications of this shift of perception vis-à-vis basin wide development.

A2: After consideration of your comment, we have changed the title from an imperative to an interrogative statement: WILL EGYPT CONTINUE TO BE “THE GIFT OF THE NILE”? Our intent in using this framing was to consider a different way to look at Egypt’s past and future water, but this softens the statement while maintaining it as a valid examination lens.

We use the framing of the 'Gift of the Nile' for several reasons: in part because Egypt's cultural and historical hegemony on Nile use still influences the way that it engages with other basin countries on the topic of water allocations, and also because the phrase suggests that Egypt's existence is possible only through the water provided in the Nile; while this may have been true in the past, the increase in trade of agricultural products and other goods makes this less true. Essentially, we point to the tipping point in the balance between internal and external water consumption as the tipping

point of the Nile being the dominant force in sustaining Egypt's continued existence and growth.

~

C3: Line 29: "The current policies on water productivity, water reuse, and population control will not be enough to close the demand gap in the future." A citation should be provided to justify this statement, or the authors should state that their analysis demonstrates this.

A3: We change the phrase "water productivity" to "irrigation application efficiency" (Line 38) for more accuracy. Irrigation application efficiency policies refer to the requirements on certain types of irrigation in certain areas (i.e., Old Lands surface irrigation vs. New Lands drip and sprinkler irrigation) that determine the country average field scale efficiency.

It is difficult to say what the effect of one change in the agricultural system could make in isolation. As we have seen above with the interplay between reuse and irrigation efficiency in primary application, there is a tradeoff in where water is used in the system. A 90% irrigation efficiency (say from a country-wide adoption of drip irrigation systems) would mean that the reuse recaptured would be smaller but quantifying that tradeoff is beyond our scope - which was an attempt to provide a reconstruction of the actual historical development. For that reason, we do not provide a hypothetical higher efficiency scenario in the paper but can present some numbers here.

As a simple case, we will look at the amount of water required for crop evapotranspiration scaled by irrigation efficiency (what we show in Figure 3a). This comparison doesn't require us to decide if this water comes from new withdrawals or reuse, just that this volume needs to be applied to the field. In our historical case, with a 2017 efficiency of 66% the efficiency scaled water application for agriculture is 68 km^3. If this efficiency was 90% this volume would be just 50 km^3.

The impact of the first two policies (irrigation efficiency and water reuse) in the historical period can be seen in

Supplementary Figure 15, where we find the effective agricultural efficiency is ~100%. Therefore, an increase in reuse in the future would be difficult and improving irrigation efficiency reduces the amount of reuse (reducing losses is of course the ideal option). Historical efforts on population control can be seen through Figure 6a where even a zero-population growth scenario (the unshaded area) adds ~50 km^3 of demand by 2035. This is due to the increased per capita demand driven by projections of economic growth in a nominal scenario (2.3% annual GDP per Capita growth).

~

C4: Line 31: "Externally, the conflict with the Nile Basin countries over water allocation and storage which focuses on construction of the Grand Ethiopian Renaissance Dam (GERD) and the sustainability of the allocations to Egypt adds further strains [2]." Poorly worded sentence. The author should not use the term 'allocations' unless they explain that this volume is only agreed between Egypt and Sudan.

A4: This sentence above is reworded as follows, to remove the comment about allocations (which are covered elsewhere): (Lines 39-42)

Externally, the conflict with the Nile Basin countries surrounding construction of the Grand Ethiopian Renaissance Dam (GERD) adds further strains [2].

~

C5: Lines 46-47: "Second, Egypt passed the carrying capacity of the Nile in the 1980's and was importing the use equivalent of at least 40 km3 of virtual water in the late 2010's [3]. Assuming persistence of the recent socioeconomic trends, we project that Egypt will likely reach an import of 61.5 km3 during this decade of the 2020s." This quotes an import of 40 km3 in the late 2010's, but only gives a reference of a book from 1997. It needs to be clearer where this number came from.

A5: The virtual water imports numbers given above (40 km^3 and 61.5 km^3) were calculated by us using the same methodology as the

other agricultural use estimates presented in the study. The reference provided is a useful but not necessary comparison to a mid 1990's literature value of virtual water imports and can be eliminated.

In accordance with this, the first sentence above is reworded as follows (Lines 111-113):

"Second, we show through our own bottom-up reconstruction of *current* water-use that Egypt passed the carrying capacity of the Nile in 1980's and was importing the use equivalent of at least 40 km^3 of virtual water in the late 2010's."

We have also added a few sentences to the results section, where we discuss the development of the historical virtual water estimate (Lines 164-167):

"Much of the increased demand has been met by virtual water imports, which reached 40 km^3 in the 2010's (Figure 2e), a figure supported by other studies that quantified historical virtual water trade [16] [19] [26]. Virtual water import is calculated identically to in-country use which is detailed in the Methods section."

~

C6: Lines 55-61: "In order to manage this process in a sustainable fashion, we recommend adoption of a hybrid national policy consisting of both Water Value Appreciation (WVA): a robust water pricing system leading to more efficient cropping patterns and enhanced irrigation application efficiency, as well as Water Share Amplification (WSA): an approach of smart and efficient management of agricultural export and import (virtual water), applicable to arid countries in general, that amplifies Egypt's share of natural water resources." Are there citations to these methods (WVA and WSA) or are these newly introduced here?

A6: WVA and WSA are newly introduced here as a way of grouping the six proposed future strategies that we present for a sustainable Egyptian water future. Water Value Appreciation

(WVA) strategies revolve around recognizing and leveraging the true value of water as a limited resource. Water Share Amplification (WSA) strategies revolve around maximizing per capita water share, which include both maximizing the utility of each water share but ensuring that issues of per capita water scarcity are managed.

We have restated how these two concepts are introduced in the paper, adding clarification for their purpose (Lines 121-131):

"In order to manage this process in a sustainable fashion, we recommend adoption of a hybrid of national policies under two umbrellas. (i) Water Value Appreciation (WVA) strategies revolve around recognizing and leveraging the true value of water as a limited resource through a robust water pricing system leading to more efficient cropping patterns and enhanced irrigation application efficiency. (ii) Water Share Amplification (WSA) strategies focus on maximizing per capita water share through an approach of smart and efficient management of agricultural export and import (virtual water), applicable to arid countries in general, that amplifies Egypt's share of natural water resources. This policy arm also employs population control measures that attempt to bring national fertility rates closer to global average levels, and external sourcing of meat from the region. These policy grouping are explored further in the discussion."

~

C7: Line 70 "Egypt's millennia long existence…" This makes it sound like Egypt has only existed for 1000 years. Please reword.

A7: We use millennia as an accepted plural to "millennium", however millenniums (offered as an acceptable substitute in the Cambridge Dictionary) may be used if it better aligns with the copy-editing practices of the journal.

~

C8: Line 76: "practically the only water resource". Can the author quantify this percentage dependency on the Nile?

A8: Yes, we change the above sentence to the following (Lines 50-51):

"At the same time, water supply from the Nile, which accounts for 98% of renewable water resources in Egypt, has remained relatively steady (Figure 1b) [4]."

[4] Food and Agriculture Organization of the United Nations. AQUASTAT Country Profile – Egypt. Water Resources Factsheet. (Rome, Italy, 2016). Available Online at https://storage.googleapis.com/fao-aquastat.appspot.com/countries_regions/factsheets/water_resources/en/EGY-WRS.pdf

~

C9: Lines 79-82: Records of flood heights from the Rhoda Nileometer show these levels remained relatively constant over the 800-year record [5] [6]. The flood heights over this period are comparable. Research has suggested "very little downcutting in Nubia since [the time of the New Kingdom some 3000 years ago]" [7]. These sentences are repetitive. No context for what "downcutting in Nubia" is supposed to mean.

A9: Sentences are rearranged as follows (Lines 54-58):

"Records of flood heights from the Rhoda Nileometer show these levels remained relatively constant over the 800-year-record [5] [6]. Research has suggested that "very little downcutting [in the riverbed has occurred] in Nubia since [the time of the New Kingdom some 3000 years ago]" suggesting that flood heights across the full 700-year record are directly comparable [7]."

~

C10: Line 83: "Dongola have been slightly decreasing, as a result of increased withdrawal of natural flows upstream [8] [9]." This should be quantified and backed up with data.

A10: We expand on this slightly, noting that Sudan's withdrawal in 1959 was 4 km^3 versus the current figure of 13-16 km^3 cited later. We edit Lines 58-60:

"In the modern record, flows recorded at Aswan and Dongola have been slightly decreasing, as a result of increased withdrawal of natural flows upstream from Sudan's withdrawal of 4 km^3 in 1959 [2] [8] [9]."

While most research in this region has focused on the basin as an interconnected system, a primary goal of this study was to conduct an Egypt-centric analysis. To do this we have used the historical record of streamflow at Dongola Station as a southern boundary condition representing the inflow of Nile water into Egypt:

Republic of Sudan Ministry of Irrigation and Water Resources (2020). Monthly Streamflow at Dongola (1963-2020). Processed and Compiled by Elzeinn, A. S.

We anchor any discussion of supply in this data and the exact mechanics of any upstream change do not affect our analysis or results. As our analysis spans a continuous time series from 1961-2017, any changes in upstream use are captured by the streamflow time series and therefore are taken into account in our analysis as a change in available supply.

~

C11: Figure 1a. On the plot, please provide a brief explanation/mention of missing data from 1400s until 1800s. Label "Discharge from Aswan Dam" and "Flow at Dongola" to clarify that these are mixed unit plots.

A11: We have changed the figure caption and plot legend to be more descriptive and have added labels to the plot to further differentiate data. Please refer to updated figure 1b shown below.

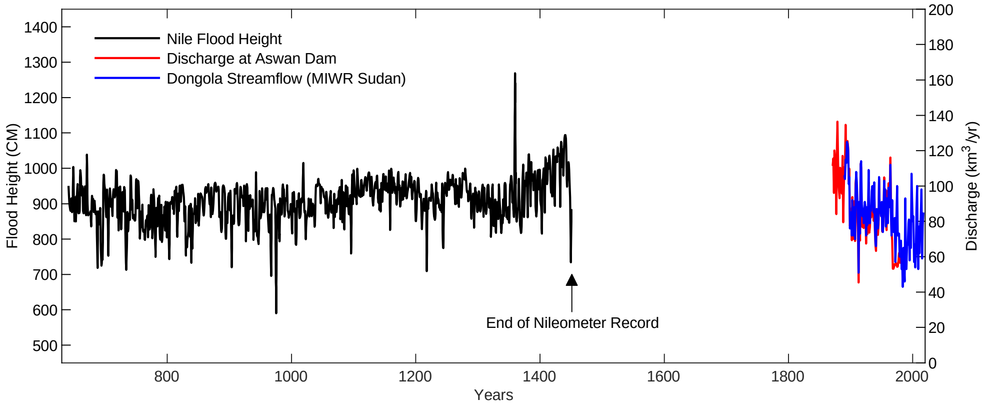

~

C12: Line 95: What is "CUT" statement?

A12: Error in the text, we have removed the statement.

~

C13: Line 99: Reference 13 is unstable.

A13: We have ensured that the link provided is active, and added more description to the reference in question, adding the figure title and year of the graphic that we are referencing. Additionally, we have added that the data and graphic was accessed in approximately August 2020. While this page is updated with new data as it becomes available, this should be able to point the reader to the appropriate figure. Please let us know if you think more should be done or if we have misunderstood your comment. The new reference [13]

Ritchie, H. Meat and Dairy Production: Meat Consumption vs. GDP per Capita, 2017. Our World in Data. (2017; Accessed August 2020) [https://ourworldindata.org/grapher/meat-consumption-vs-gdp-per-capita]

~

C14: Line 107: "The rapid changes in Egypt's water demand have spurred equally large responses…" Rapid and large are not synonymous. Please reword.

A14: Removed “equally” from the sentence

~

C15: Lines 109-112: “… an attempt to control and manage water supply and demand. Egypt’s efforts (documented in Figure 2) have been substantial in five areas” In the introduction there were 4 “avenues” mentioned and did not include the attempt to control population growth. These two lists should align, possibly by adding this element to the introduction list.

A15: The introductory paragraph has been changed to be more consistent (Lines 104-108):

“demand through five avenues: improving water infrastructure and management by building the High Aswan Dam; increasing in-country agricultural production through harvested area expansion and improving crop yields; expansion of water reuse; reducing fertility rates to control population growth; and increasing import of agricultural products - especially staples such as wheat and maize.”

~

C16: Figure 2. The inconsistency in date ranges should be remedied.

A16: The differences in data ranges are due to the differences in data availability and our desire to show all data that may be relevant. However, changing the data ranges for panel d to 1961-2017 will align it with panels b and e does not alter the picture presented through the data. The other panels either have a much shorter range of available data (panel c) or require a larger range to make the point that we are trying to make with their inclusion (panel a). Please see the modified Figure 2 below:

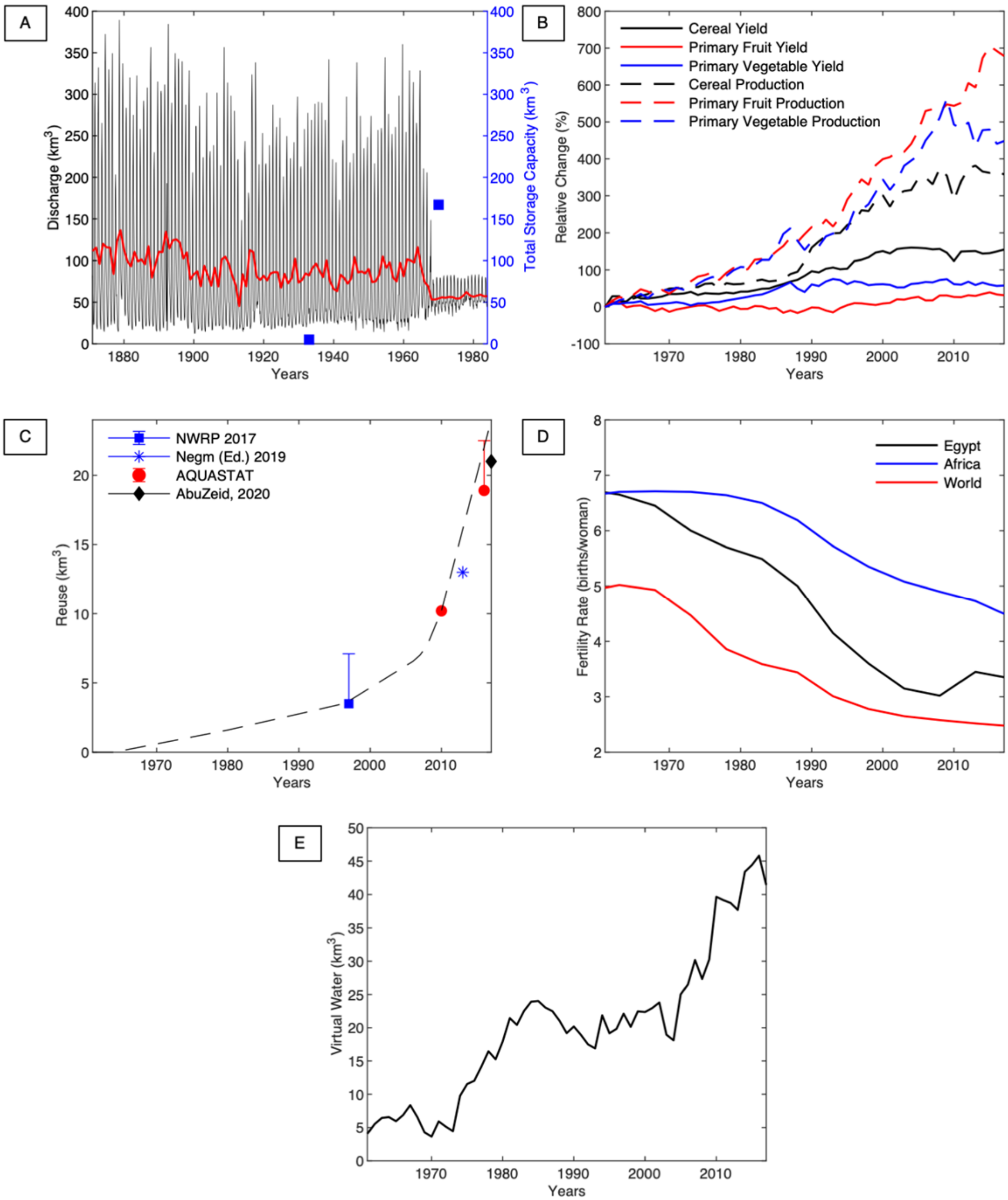

~

C17: Figure 2a: This is confusing to me. First, it looks like the annual flows at Aswan dropped from an average between 80 to

100 bcm, to around 50 bcm. Are these only a result of Evaporation, which is estimated to be 10-12 bcm. Perhaps Sudanese withdrawals? The author needs to quantitatively explain the difference. The data sources for this is from 1998. The authors should extend this to include the most recent years. Also, the storage dots do not say much. This would be better if it showed the increasing storage in the entire basin up until the recent time (Roseries, Sennar, Merowe, Khashm El Girba etc.).

A17: In Figure 2a the average flow at Aswan from 1900-1959 is 84 km^3, which is also the amount enumerated as the long-term flow in the 1959 Agreement. The flow at Aswan post dam completion from 1970-1984 is 57 km^3. This leaves 10 km^3 as evaporation, and the remaining 17 km^3 partitioned between Sudan's usage, and further increases to the storage volume of Lake Nasser. The citation below shows that the level of the lake continued to rise since the dam's completion in 1970 to the mid 1980's:

Gammal, E. A. E., Salem, S.M., and Gammal, A. E. A. E. Change detection studies on the world's biggest artificial lake (Lake Nasser, Egypt). *The Egyptian Journal of Remote Sensing and Space Science*, **13** (2) 89-99, (2010). https://doi.org/10.1016/j.ejrs.2010.08.001.

The data for the flow at Aswan is available until 1984 at the source used, and demonstrates what we intended within the provided range -- that Egypt exerted substantial control on the flow of the Nile measured at Aswan. The exact timing of the releases from the High Aswan Dam during and after this period are not investigated here, since this is a management issue rather than a supply issue. Please refer to answer A10 as to our use of the data at Dongola for the analysis.

We use the blue data points to show the substantial increase in storage volume represented by Lake Nasser, and we have not expanded the storage picture so as to not get into issues of storage and management changes in the Basin as a whole.

~

C18: Figure 2e: What is the data source for Virtual Water imports?

A18: Please see answer A5

~

C19: Lines 120-121: "M.M. Mekonnen and A.Y. Hoekstra" - Names are not needed. It should be sufficient to show reference numbers.

A19: Removed "by M.M. Mekonnen and A.Y. Hoekstra

~

C20: Figure 3. The total volume from of water use in Figure 3a is somewhat different than 3b, which should always be higher since it combines municipal and industrial. What is the data source of the use line in Figure 3b? Please make figures clear that this only for Egypt.

A20: Figure 3a shows the necessary agricultural water withdrawal -- i.e., the crop ET need modified by the country average field scale efficiency. However, in order to preserve the focus on the water share of individual crops, this panel does not include the withdrawal savings that come from *reuse.* The inclusion of this reuse amount, despite the addition of other uses such as municipal and industrial, actually causes a decrease in the projected withdrawal needed when all of these components are considered in panel b (red line). We have added clarification to the caption for Figure 3 that these figures depict water use in Egypt, and that the red line in panel b takes into account reuse volume.

~

C21: Line 125: "Egypt began fully utilizing available water resources in the 1980's and has only met increasing agricultural water demand through virtual water imports (dark grey shading) and increasing reuse (light red shading)." This makes no mention

of the increasing uses is Sudan, which is a highly debated subject, particularly the volume of current upstream depletions. The wording of “available water resources” allows this statement to be technically accurate, however there should at minimum be a mention that these available water resources include all water not consumed upstream, which is dynamic. Bringing in the topic of ‘basin closure’ would also be worthwhile here. Falkenmark, M., Fraiture, C.d. and Vick, M.J. (2009), Global change in four semi‐arid transnational river basins: Analysis of institutional water sharing preparedness. Natural Resources Forum, 33: 310-319. doi:10.1111/j.1477-8947.2009.01254.x

A21: Sudan’s usage is indeed uncertain, however understanding Sudan’s usage through time is beyond the scope of the study. Please refer to answer A10 for our usage of streamflow at Dongola as an inflow boundary condition that reflects any relevant upstream changes.

A full investigation of basin water balance at the scale of the Eastern Nile is important but is beyond the scope of this Egypt-centric study.

In order to emphasize that the black line in 3b (available supply) includes the variable amount of water not consumed upstream we have modified the sentence below (Lines 141-143)

“Figure 3b shows the agricultural use estimates combined with other water demands (municipal, industrial, reuse), compared to the available Nile water flow into Egypt which encompasses all water not consumed upstream.”

~

C22: Line 132: “we show that Egypt’s direct consumption of the Nile is roughly 61.5 km3 on average from 1988 to 2017.” Does the 61.5 bcm relate to the data on Figure 3a or 3b?

A22: 61.5 km^3 refers to the 1988-2017 average Historical total Nile Water use number (average of the red dotted line) in figure 3b. Please refer to answer A20 for further clarification of the

difference between Figure 3a and 3b. We have added a reference to Figure 3b at the end of the quoted line above in the manuscript.

~

C23: Lines 132-135: "Adding the environmental flow to the Mediterranean of about 2-4 km3 [26], Egypt consumes 8.0 to 10.0 km3 more than the share of 55.5 km3 allocated through the 1959 Nile Agreement between Egypt and Sudan." I believe the author is considering the flows to the sea as a consumption. Perhaps "releases" is a better word than consumption. This should be backed up with any available data.

A23: Changed the word "consumes" to "utilizes", since the Mediterranean discharge is indeed more of a use than a consumption. We were not able to find public data for measured releases into the Mediterranean and therefore rely on the cited literature [once 26 now 20] for the value. The other 6 km^3 is documented in the results through our reconstruction of total historical Nile Use in agriculture and other areas.

~

C24: Line 135: "This additional water comes partially from Sudan's unutilized share of 4-5 km3 [27], and partially from increases in the Nile flow of about 5-6 km3 [28] [29]." The author should specify that this share is relative to the 1959 agreement, which is not recognized by other riparians. The authors should also recognize the uncertainty of the current Sudanese depletions. The link to the NBI source is not working.

A24: We have edited this section to read (Lines 160-164):

"This additional water comes partially from Sudan's unutilized share of 4-5 km^3 out of the 18.5 km^3 enumerated in the 1959 Agreement [21], and partially from increases in the Nile flow of about 5-6 km^3 [9] [22] [23]. An accounting of Sudan's historical use of the Nile water is beyond the scope of this Egypt centric study but is reported to be 13-16 km^3/yr [24] [25]."

And have added the following references to Sudan's estimated water withdrawals from the Nile:

Omer, A. M. Water resources and freshwater ecosystems in Sudan. *Renewable and Sustainable Energy Reviews.* **12**, 8 (2008) https://doi.org/10.1016/j.rser.2007.01.005.

Salman M.A. Salman. The new state of South Sudan and the hydro-politics of the Nile Basin. *Water International*. **36**, 2 (2011) http://doi.org/10.1080/02508060.2011.557997

We have also fixed the link to the NBI source.

~

C25: Lines 138-140: "This schematic relies on several independent sources of data, each flux is uncertain, and hence does necessarily satisfy strict water balance." These schematics are a major contribution, that will get a lot of attention. However, I'm concerned that they do not satisfy a water balance and the sources are not sufficiently documented.

A25: We have added a specific list of references and calculation methods for every element of Figure 4 (See caption) and have updated some of the analysis to show a balance that closes while acknowledging the uncertainty within the estimates (i.e. added ranges of possible values). Chief in this update is a use of published Lake Nasser height data for 1988-1992 from the source below:.

Gammal, E. A. E., Salem, S.M., and Gammal, A. E. A. E. Change detection studies on the world's biggest artificial lake (Lake Nasser, Egypt). *The Egyptian Journal of Remote Sensing and Space Science,* **13** (2) 89-99, (2010). https://doi.org/10.1016/j.ejrs.2010.08.001.

Additionally, we use new equations for the calculation of Lake Nasser Area and Volume from the citation below rather than our previous approximation from rating curves:

Shafik, N. M. Updating the surface area and volume equations of Lake Nasser using multibeam system. Presented at 19th International Water Technology Conference (2016) [http://iwtc.info/wp-content/uploads/2016/05/41.pdf]

We have also updated our inflow dataset of Dongola streamflow to use a more recent, more carefully calibrated version provided to us by the MIWR in Sudan:

Republic of Sudan Ministry of Irrigation and Water Resources (2020). Monthly Streamflow at Dongola (1963-2020). Processed and Compiled by Elzein, A. S.

Please see the updated Figure 4 below.

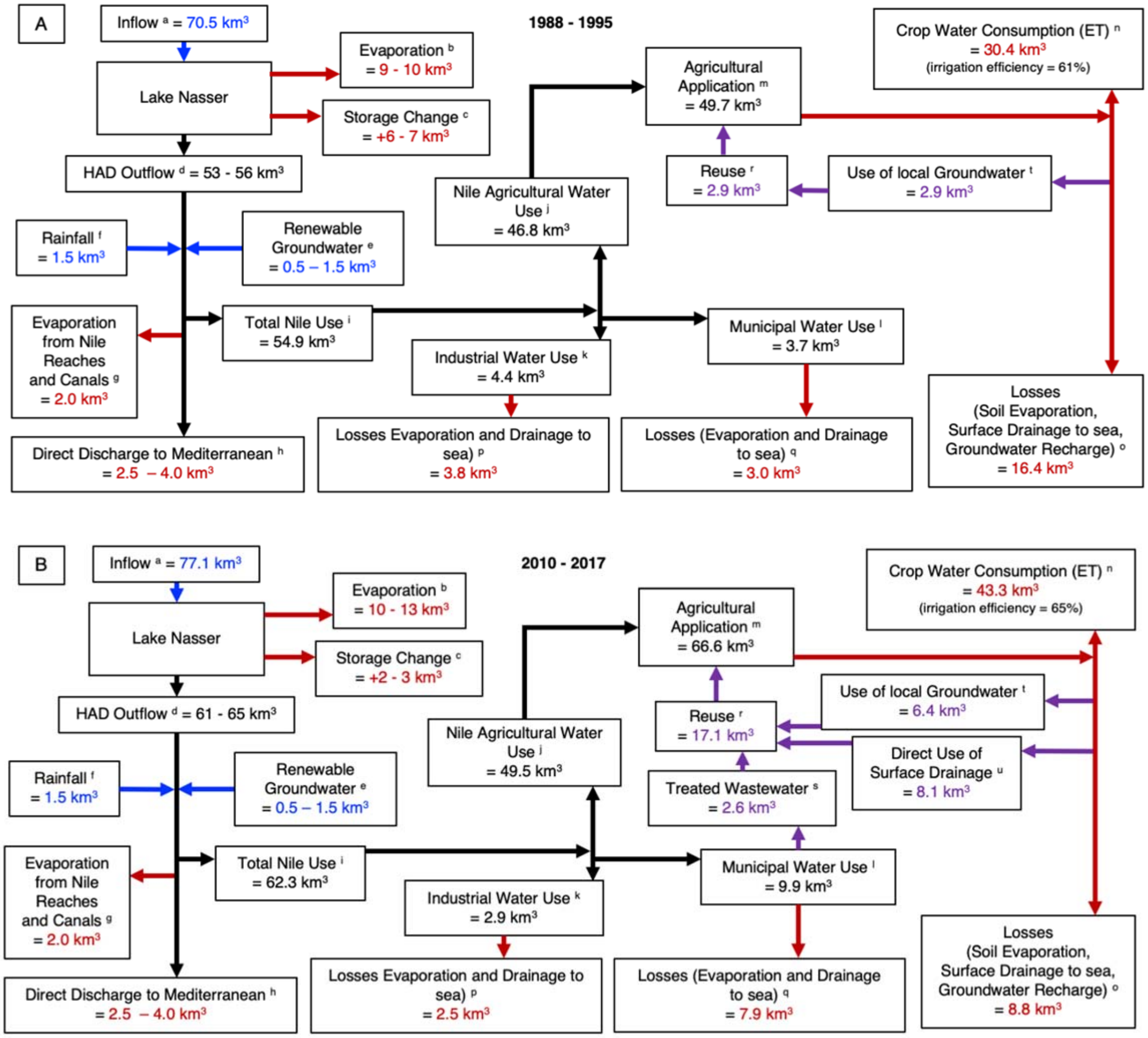

~

C26: Figure 4: Are the storage changes positive or negative?

A26: The changes are positive and plus signs have been added to indicate this is Figure 4.

~

C27: Figure 4b: Mass balance for 2010-2017: Total Nile Use does not fall in the possible ranges provided. How can the total Nile use be 62.3 when the water available (HAD outflows (60.1)+rainfall (1.5)+ max GW (1.5)) = 63.1 bcm and the losses

(Reach and Canal evap (2.0) + min Med Sea discharge (2.5)) are 4.5 bcm, leaving only 58.6 bcm to divert? I understand that it is impossible to get exact numbers, but the ranges provided should allow a possible mass balance. Overall, I'm concerned that the lack of mass balance will lead to confusion. Please review all the numbers in Figure 4 carefully and provide a citation for each in the supplementary materials so the reader can understand how they are derived.

A27: Please refer to A25.

~

C28: Figure 5: What does (use eq.) mean in the caption? On Figure 5b, what are the units of the numbers on the dashed lines of the plot (bcm I presume).

A28: Deleted (use eq.) and added to the caption of Figure 5b that the units of the dashed lines are km^3.

~

C29: Line 142 and Figure 6: The authors must be explicit of their assumptions on further upstream development in Sudan and elsewhere.

A29: Please refer to answer A10 on the framing of our analysis as Egypt centric and the use of inflow boundary conditions that capture all upstream changes that are relevant to our analysis.

Our projections on future water needs in Egypt focus only on projected demand and are not impacted by changes in assumed supply. We do note the measures that can be taken to increase the supply and circumstances that may reduce it (building of the GERD, climate change, etc.) however the quantitative evaluation of these developments is not covered here because it does not impact our analysis of future demand, which is developed as a function of population and economic growth in Egypt.

~

C30: Lines 182-190: "We recommend water demand management through the lens of Water Value Appreciation (WVA) including: (1) implementing new water pricing strategy, (2) improving application efficiency at the field scale, and (3) adjusting cropping patterns to emphasize ecological suitability and water use efficiency. In addition, we recommend further changes that are guided by a Water Share Amplification (WSA) which focuses on (1) amplifying virtual water share in the global food market, (2) increasing cooperation with Nile basin neighbors to import animals and animal products (meat) instead of using valuable water in producing them locally, and (3) bringing fertility measures in line with the rest of the world to control population growth and amplify per capita water share." It seems strange that the authors are introducing these topics in the discussion, yet they have not been analyzed in the results. WVA and WSA are described in the methods and supplementary materials, but they since they seem to be central to the arguments, they should be considered results that are reflected upon in the discussion.

A30: Please see answer A6 for the use of WVA and WSA as groupings of proposed qualitative solutions. We realize that we have used WSA in two related but confusing ways as our thinking evolved - one as a grouping of three solutions, and also as a quantitative metric for Egypt's virtual water trade balance. We have removed the sections under *Water Share Amplification* that relate to this quantitative metric (Lines 297-308, starting "Water Share Amplification (WSA) is introduced here as a concept…), renaming them Virtual Water Trade Balance (VWTB) and adding a Supplementary Note 2 where this analysis may be useful to readers. We have kept a section discussing the balance of trade in the paper but kept the discussion qualitative. This should also remove the introduction of new results from the discussion section.

~

C31: Lines 200-202: "There is therefore an argument to implement water pricing for farmers and tie it to either the marginal cost of increasing water supplies or to the cost of increasing water use efficiency (though upgrading or maintaining delivery systems

and irrigation methods) [32]." Use of passive voice is problematic here because it masks if you are advocating this or whether you are simply pointing out that He et al (2006) is suggesting this.

A31: Sentence has been rewritten in active voice. An additional sentence has been added to clarify the use of reference [32] now [29] (Lines 231-235):

"though other have noted the obstacles to increasing this efficiency [29]."

~

C32: Line 204: "Egypt dramatically increased its crop yields through the use of more water and the application of agricultural technologies such as fertilizer." The author should quantify this by referring to the results or other literature.

A32: First we add a note pointing the reader to Supplementary Figure 2 which shows the yields of all major primary crops used in the analysis. We also added a reference regarding Egypt's crop yield increases [30]. The sentence now reads (Lines 237-239):

"From 1980 to 2000, Egypt dramatically increased its crop yields through the use of more water and the adoption of agricultural technologies such as fertilizer (Figure 2b; Supplementary Fig. 2) [30]."

~

C33: Lines 207-208: "Water productivity (tonnes/m3) did not improve as drastically until the more recent aggressive perusal of reuse technologies." The author should cite evidence in their analysis or other sources if possible.

A33: We show the reconstructed water consumption (m^3/tonne) requirements of each crop in Supplementary Figure 3.

Additionally, we show the relative change in water consumption requirement for major crops (tonnes/m^3) in Supplementary Figure 4. In panel 4c, when considering both changes in country average irrigation efficiency and reuse amounts, the change is dramatic and accelerates after the 1990's when reuse amounts began to increase (Fig 2c). A reference to these supplementary figures has been added at the end of this line (Lines 240-242).

~

C34: Line 215: "Reducing conveyance and application losses are key in increasing water" While true in principle, the governance challenges to making efficiency improvements. The authors should note these obstacles.   Grafton, R. Q., Williams, J., Perry, C. J., Molle, F., Ringler, C., Steduto, P., . . . Allen, R. G. (2018). The paradox of irrigation efficiency. Science, 361(6404),

A34: We have expanded on this section and included the reference provided to note potential challenges in formulating policy in this area. (Lines 249-251):

"Reducing conveyance and application losses are key in increasing water productivity in agriculture [26] [32] however there are policy challenges in incentivizing change and ensuring that savings in one sector do not incentivize waste in another [33]."

~

C35: Line 218: "There is a limit to this efficiency increase, as more efficient application reduces the amount of available reuse." Is this itself a limitation or simply an implication of becoming more efficient?

A35: It is an implication more than a limitation, since ideally water would be efficiently used at the point of initial application, and not be required to be recaptured and possibly treated before being reused. However, we still think the following reduce statement is worth saying:

More efficient application necessarily reduces the amount of available reuse.

~

C36: Lines 221-222: "their amount of reuse equaled the excess with drawn that is not used in direct plant evapotranspiration" What is the value of this comparison?

A36: The sentence was reworded as follows, to emphasize that the large amounts of reuse catch most of the excess withdrawal (Lines 256-258):

"… in 2017 they captured and reused as much as the applied agricultural water that did not go to plant evapotranspiration (Supplementary Figure 15)."

It emphasizes that although the average field efficiency (i.e., the amount of water withdrawn from the Nile that is actually consumed by the plants through ET) is relatively low (~65%) the reuse of that water through drainage capture, or shallow groundwater pumping actually increases the effective efficiency of the system significantly.

~

C37: Lines 226-230: "Subsequently, selection of crops that are ecologically suited and easily irrigated using high efficiency measures should be a key goal for future agricultural policy. For example, the expansion of olives is an option as a crop that is well suited to the regional climate and to the use of drip irrigation methods. This expansion is already planned for Egypt, and their efficiency under drip irrigation suggests a promising role in Egypt's export portfolio [37] [38]." To what extent (quantitatively) can efficiency improve the situation? Without numbers, this becomes an obvious assertion.

A37: It is difficult to say what the effect of one change in the agricultural system could make in isolation. As we have seen above with the interplay between reuse and irrigation efficiency in primary application, there is a tradeoff in where water is

used in the system. A 90% irrigation efficiency (say from a country-wide adoption of drip irrigation systems) would mean that the reuse recaptured would be smaller but quantifying that tradeoff if beyond our scope - which was an attempt to provide a reconstruction of the actual historical development. For that reason, we do not provide a hypothetical higher efficiency scenario in the paper but can present some numbers here.

As a simple case, we will look at the amount of water required for crop evapotranspiration scaled by irrigation efficiency (what we show in Figure 3a). This comparison doesn't require us to decide if this water comes from new withdrawals or reuse, just that this volume needs to be applied to the field. In our historical case, with a 2017 efficiency of 66% the efficiency scaled water application for agriculture is 68 km^3. If this efficiency was 90% this volume would be just 50 km^3.

Please see A3 for related discussion.

~

C38: Line 234: "Egypt needs to strive further to reduce fertility rates and slow population growth." Yes, this an important issue, but why is this included in Water Share Amplification?

A38: We include population growth in the water share amplification umbrella because it directly relates to per capita water availability (i.e. per capita water share) and how that will change in the future.

~

C39: Line 253: "Water Share Amplification (WSA) is introduced here as a concept of maximizing the water available to a country, both in terms of water productivity and in terms of market value." Introducing an analytical method into the discussion? Why wasn't this presented in the results? Is this a new method?

A39: Please refer to answer A30

~

C40: Lines 261-263: "Using a set of water share amplification metrics calculated for current crops produced and traded we develop a WSA proposal presented in Supplementary Table 5." The author is still presenting new analysis and solutions even at the end of the discussion. Seems like they are trying to pack in too much.

A40: Please refer to answer A30

~

C41: Lines 297-300: "Egypt will need to compensate for roughly 15-20 km3 of losses over the next several years (4.9 km3 and 13.5 km3 of filling related losses in the first and second years respectively) from the filling of the Grand Ethiopian Renaissance Dam (GERD) [44]." The notion of a loss due to the GERD that needs to be compensated during the filling is highly dependent on the hydrologic conditions during this period, management practices, agreements between the countries, and what constitutes a loss. For example, it is unlikely that the first-year filling had any negative implications with respect to water shortages, and a subsequent hear in 2021 might result in the same. The focus of this article should remain on the long-term supply-demand imbalances and the authors should not casually analyze the transient implications of the GERD. I also note the authors have acknowledged in previous publications that increased storage may have positive implications for Egypt as well. Referencing news articles (with broken links especially) on this topic is not appropriate when much peer-reviewed literature exists on this topic.

A41: We agree that analyzing the acute impacts of the GERD filling are beyond this paper and we have removed this section and reference [44]. We have also removed Supplementary Note 2 (Filling of the Grand Ethiopian Renaissance Dam (GERD)) from the Supplementary Material PDF.

~

C42: Line 303: "Demand and supply of water will also be affected by climate change." A mention of climate change is appropriate here because of the author knowledge, citations used, and the potential implications on the supply-demand imbalance, but the paper should explicitly mention that these elements are not analyzed here.

A42: The first sentence of the paragraph discussing potential climate impacts has been changed to "Demand and supply of water will also be affected by climate change, although we do not analyze those changes in this study." (Lines 349-350)

Reviewer 2

C43: Including discussion of the potential impacts of sea level rise on Nile delta agriculture earlier in the paper (in now only comes up in the conclusion) as an added dimension to water insecurity.

A43: We prefer to keep the potential impacts of climate change grouped together as an additional consideration in the conclusions. This is partly because we do not analyze the effect that this rise will have in the paper (similar to any other climate impacts), but also because our future analysis is wholly focused on changes in demand based on population and economic growth and does not delve into issues of supply change

~

C44: Change the language around (or clarify) "aggressive population control". The language, along with the reference to Asia's dramatic population decrease, seem to imply that the author's are encouraging the use of the one child policy in Egypt. I find this discussion to be the most problematic part of the suggested recommendations and seems like it was inserted last minute.

A44: We agree that the framing of population control as "aggressive" is unnecessarily strong and have changed the

language accordingly. Asia was included as a comparison as the most recent continent to go through similar agricultural and demographic change to what Egypt has experienced in the last several years. However, it may be more appropriate to stick to the comparisons with overall African and Global fertility that we show in figure 2d. Therefore, we rephrase “aggressive population control” as “population control measures that attempt to bring national fertility rates closer to global average levels” (Lines 129-131). We also note that “Asia, another region that has developed substantially in the last several decades and experienced an agricultural revolution has managed to reduce its fertility rate from 5.8 to 2.2 from 1952 to 2018, albeit through the use of more aggressive and controversial strategies [36].” (Lines 272-276).

~

C45: The paper mentions Egypt's reliance on the unused portion of Sudan's water allocation under the 1959 Nile Agreement. 1-2 sentences on how regularization of the flow of the Nile downstream of the GERD may increase Sudan's water use for agriculture (there are several papers on this) would strengthen this discussion.

A45: Please refer to C/A10, C/A24, and C/A41

~

C46: I don't agree with the assertion that Egypt importing more virtual water challenges its historical characterization of Egypt as "the gift of the Nile", and I worry that the title will distract people from the theme of the paper and that you may actually lose your target audience (i.e. Egyptian water policymakers). [But that's a stylistic difference in opinion.]

A46: Please refer to C/A2

~

C47: Figure 4 is a really interesting - I haven't seen such a clear schematic on water use in Egypt and this by itself is an

exciting contribution. I can't comment on the accuracy of the numbers, but assuming they are accurate, it is a very rich figure, but you should explicitly include the sources for these numbers (and how they're calculated).

A47: Please refer to C/A25 & C/A27

~

C48: It may be outside the purview of this paper, but some discussion of which policies may be more politically feasible than others would be valuable.

A48: Yes, unfortunately a more in-depth analysis of these policies is beyond the scope of this paper but are certainly interesting avenues for future work.

~

C49: Some reference to Zeitoun et al's (2009) work on virtual water trade in the Nile basin could strengthen the paper, especially in its discussion of increasing cooperation with Nile basin neighbors to import animals and animal products.

A49: We have added the following sentence and a reference to Zeitoun et al. (2010) (Lines 316-318):

"On average in 1998-2004, Egypt imported the equivalent of 18.3 Mm^3/yr of virtual water in sheep from Sudan, although livestock movement across the Egypt-Sudan border is often under documented [42]."

[42] Zeitoun, M., Allan, J. A., & Mohieldeen, Y. Virtual water 'flows' of the Nile Basin, 1998-2004: A first approximation and implications for water security. *Global Environmental Change*. **20**, 2 (2010). http://doi.org/10.1016/j.gloenvcha.2009.11.003

Comments from PDF:

C50: “was importing the use equivalent of at least 40 km3 of virtual water in the late 2010’s [3]. Assuming persistence of the recent socioeconomic trends, we project that Egypt will likely reach an import of 61.5 km3 during this decade of the 2020s.” The most recent official figure I heard from Egypt was 35 BCM of virtual water. Where does 40 BCM in late 2010s come from? It can't be from the reference, because that was written in 1997.

A50: The 40 km^3 was calculated by us, using the methodology detailed in the Results and Methods section. The discrepancy could be for several reasons, likely due to how the virtual water amount was calculated. We make several assumptions in our Virtual Water calculation in order to make the numbers consistent with numbers involved with in country use and agricultural production. First, the virtual water is given in terms of Egypt use equivalent. The calculation of virtual water depends on where a crop was grown, and what cultivar that crop may be. For consistency we put everything in terms of how much water it would have taken to grow those imports in Egypt. In line with this, we also apply the irrigation efficiency of the system to this water so that we can compare these hypothetical withdrawals to the historical construction of withdrawals for in-country agriculture, etc. For further info please see C/A5.

~

C51: Line 100: Some discussion of what that translates to in terms of water use would be helpful... eg. average water use of producing red meat is X times greater than the average water use of producing white meat.

A51: We prefer to keep the discussion in this section focused on the changes in diet alone, but the reader may refer to Supplementary Table 3 (In “Supplementary Data”) which provides the water requirements for all major commodities used in the analysis, including beef (red meat) and chicken (white meat).

~

C52: Figure 4: Include the years here (in addition to the subtext below). Direct discharge to the Mediterranean has remained the same for these two time periods?

A52: We add the years to the figures themselves. Unfortunately, we were not able to locate a reliable source for the change in discharge to the Mediterranean over time and therefore use the single number. However, it is reasonable to assume that flow to the Mediterranean has been much smaller and less variable since the completion of the High Aswan Dam in 1970.

~

C53: Some discussion of existing agreements to import livestock from Ethiopia and other upstream Nile countries would be helpful.

A53: Please refer to A49

~

C54: Lines 199: The paper hasn't mentioned the share of agricultural employment in Egypt yet - some discussion of why agricultural production is important to livelihoods (and how that is related to the political feasibility of raising tariffs) would be helpful.

A54: The importance of agriculture in the labor sector is definitely a concern when considering changes. To note this, we have added to the following sentence (279-281):

“There is resistance to increasing imports of food products - especially staple goods such as wheat - due to national security concerns, as well as the importance of agriculture in the labor sector [38].”

~

C55: Interesting point. Also, Egypt considers using groundwater as reuse (i.e. does it count groundwater use as part of its

agricultural runoff water recapture)? That is surprising/misleading.

A55: Reuse here has three components, which are all reported separately in AQUASTAT and other sources: direct collection of agricultural drainage water, the pumping of shallow groundwater recharge (predominantly in the Delta), and the treatment and reuse of wastewater (mostly municipal). We account for all three of these reuse sources as separate components to overall reuse (Supplementary Figure 9).

~

C56: Why are you comparing with Asia instead of other parts of MENA or SSA? Also, how much of this reduction was related to the one-child policy in China? Culturally, this is not a feasible option for Egypt (or politically). Also, without explicitly suggesting alternative ways in which population can be reduced, the paper seems like it is suggesting implementing something like the one child policy in Egypt.

A56: Please see C/A44

~

C57: Line 242: CPF?

A57: Country Programming Framework. We have written out the acronym instead.

~

C58: Can you add some text to explain why you're focusing on wheat imports (and Russia)? Also, have you reviewed Zeitoun et al's (2009) work on virtual water trade in the Nile basin? It may be helpful to make some reference to their paper and briefly describe how your paper is adding to their discussion.

A58: We focus on wheat because it is the largest import, and Russia is the largest supplier. We have edited the main sentence in this section slightly to reflect this choice (Lines 286-288):

"Currently 69% of Egypt's main import, wheat, comes from Russia, and roughly 90% from Russia and former Soviet Republics [40]."

A reference to Zeitoun et al. (2009) has been added in response to C49.

~

C59: Line 266-267: "One opportunity for Egypt to increase their imports of virtual water while saving water internally is through the import of meat from neighboring countries, particularly Sudan." Great idea, but then Sudan would potentially use more water, reducing the amount of unutilized water from the 1959 Nile Agreement (which is something Egypt is already concerned about w/respect to Sudan increasing its agricultural development in response to regularization of Nile flow due to the GERD). What about increasing their meat imports from other upper Nile countries to promote more regional cooperation in general?

A59: Please see C/A41 and C/A45 for more information on Sudan's uses and interaction with the GERD, and C/A49 for references to meat imports.

While it wasn't a topic of much research in this paper, Sudan's higher level of rainfall also provides a better opportunity for animal grazing on rainfed pasture lands versus Egypt's drier climate.

~

C60: It exceeded full utilization, according to the paper (i.e. exceeded its allocation of 55.5 BCM). Some discussion of how Sudan's future agricultural production may affect Egypt's reliance on the unutilized portion would be helpful to include somewhere.

A60: Please see C/A29 and C/A45

~

C61: Worth mentioning that if the EN countries do reach agreement on the GERD, storing water in Ethiopia could reduce evaporative losses compared to storing water at HAD (although it currently seems politically infeasible).

A61: Please see C/A41

REVIEWER COMMENTS

Reviewer #1 (Remarks to the Author):

The article has been significantly improved in this revision. The analysis itself shows snapshots in time of Egypt's water use, and this is the biggest contribution of this paper. There are some additional issues that need to be addressed however. Most are editorial in nature, and few of substance.

The main concern is messaging of the work. The argument of highlighting the greater dependency of Egypt on the Virtual water compared to the Nile is interesting, but it does not mean that Egypt isn't going to be dependent on the Nile. This should be clear.

The second thing I struggled with are the 'recommendations', which are not really part of the analysis done. WVA and WSA are proposed solutions, but not described in this paper, leaving some of the recommendations somewhat normative. Are there any references that the reader can go to to learn more about these methods? If the authors really want to promote these as solutions, then a more explicit description should be provided in the supplementary materials. It would be preferable however to have these methods published elsewhere before introducing them here superficially. I recognize that adding any kind of an in-depth analysis here of solutions would greatly exceed the permitted length of the article.

The third issue is the values used for inflows into Lake Nasser. These has changed since the previous draft.
In the previous version:
1988-1995 = 71.4 km3; 2010-2017 = 73.1 km3
In the revised version:
1988-1995 = 70.5 km3; 2010-2017 = 77.1 km3
Existing data sources indicate:
1988-1995 = 70.2 km3; 2010-2017 = 72.4 km3

These differences must be reconciled before this can be published. The authors need to confidentially provide the original data for Dongola flows used in this paper for a comparative review with other reliable sources.

Note your framing is being used, but its still a good one. "9.6.7 Can the Nile Take Its 'Gift' Back?" in https://link.springer.com/chapter/10.1007/978-3-030-03698-0_9

Additional line-by-line comments are provided below:

Line 10-12 – Eliminate the first sentence of the abstract. It doesn't add value.

13 – Prospers? That is debatable, depending on your frame of reference. Use another word?

14 – Carry? That is a strange word to use.

14 – Best to specify "on how water demand for water in Egypt outstripped…."

15-16 – Strange to put what is well documented in the abstract. Words are precious. Use them succinctly and powerfully here

17 – "engages the drivers…" – strange wording

18 – "project show"?

31 – Avoid insinuating 'pressures on water rights' when that is under the topic under debate. 'perceived water rights' is a better way to go.

34 – "Current policies on irrigation…" You need a citation here. Watch your wording on "population control". That can get backlash. 'Family planning?' Use the most politically acceptable term that is used in Egypt.

35 – Citation 2 is the 1959 Treaty, not on the GERD. Use something recent. Also, you might want to mention that the impacts from the GERD on Egypt are under extensive debate.

37 – The first sentence is strange. How does Egypt's long existence actually give us insight on current water pressures? Are you talking in a relative sense? Nothing it the paragraph helps make that connection, other than stating the obvious. More people, more demand. That is not 'insight'. This paragraph seems strangely incomplete.

52 – "From Sudan's withdrawal of 4 km3 in 1959". A current estimated use in Sudan is 16.7 bcm, which does not include evaporation losses (cite https://www.nature.com/articles/s41467-020-19089-x). I understand you may want to avoid this difficult issue, but there is no need to state the withdrawal in 1959. This must reflect the latest research.

54 – You've gone from a brief mention of demand, to discussion supplies, and now back to the drivers of demand. Seems odd. This long paragraph is all about food and the evolving diet of Egypt. All good information, but it needs to keep relating back to water. I suggest reversing this paragraph with the previous one, so you talk all about demands and the drivers, then about the supplies.
Figure 1a/b. Would be great to add when Herotodus said the quote on the plots, but I believe it was ~400 BC. So that might distort things too much.

Figure 1b – Is there any basis by which these two y axes and scales were aligned? Could be misleading. Please explain!

Figure 1d – What is the basis for the relative increase? Is it the 1960 single point value? An average over a period? This needs to be clear.

84 – "This response is discussed…" I'm not sure if you are talking about one of these 5 (these) or just the last one.

87 – This paragraph on positioning your paper amongst existing literature is awkward. It oscillates between stating what it does in addition to existing work but doesn't really spell out much of what that is, except for citation 16 (Abdeldakir et al 2018). Nothing else in the history of Egypt? Government planning documents?
Egypt MWRI. (2005). Water for the Future: National Water Resources Plan for Egypt - 2017. Retrieved from http://extwprlegs1.fao.org/docs/pdf/egy147082.pdf, Cairo:
NWRP. (2017). The national water resources plan NWRP 2017-2030-2037 Egypt. Cairo: The Ministry of Irrigation and Water Management.
I'm not disagreeing with the value, but the paragraph doesn't make a very convincing argument.

115 – Seems like you should mention the 3 periods of analysis, past, present and future, in the introduction.

116-120 – This list of 5 items was just described in the introduction (lines 81-85). Seems a bit strange.

126 – "Reach an import"… ? Grammar.

141 – Terms like "Population control" may raise concerns.

Figure 3 - The "dark grey shading" needs to be more visible and explained better. Why is there a range? In 1960, the red line (total Nile Water Use) is the same as the Virtual water import? Is this a coincidence? Is the top of the grey shading the total demand for water from the Nile and from Virtual water imports?

158 – Make sure the reader understand this is the first conclusion

167 – How do these numbers align with Egypt's own mass balance estimates? (For example, Egypt estimates their outflows the sea around 12.5 bcm.)
Molle, F. (2019). Egypt. In F. Molle, C. Sanchis-Ibor, & L. Avellà-Reus (Eds.), Irrigation in the Mediterranean: Technologies, Institutions and Policies (pp. 243-277). Cham: Springer International Publishing.
NWRP. (2017). The national water resources plan NWRP 2017-2030-2037 Egypt. Cairo: The Ministry of Irrigation and Water Management.

169 – It is suggested to not try to quantify how much of Sudan's share is 'underutilized' since there is significant debate on that issue with respect to accounting for evaporation losses.

172 – Include up to 16.7 bcm.

177 – Figure 3 - No need to list all the fruit and vegetables independently here. I imagine this could be in an appendix or methods somewhere. Does Figure 3a 'application' include water from the Nile + re-use? I assume the Total Nile Water Use line in Figure 3b is derived using the same information on Figure 3a, but with municipal and industrial added, and reuse subtracted. By that logic, I guess this is why the red line in Figure 3b starts higher, but ends lower than the sum of the applications in Figure 3a. I guess this begs the question then why is the top of the red band in Figure 3b over 80 bcm in 2017, but the total water applied to agriculture is around 70 bcm in 2017 (Figure 3a).

198 – What happened to the "current" section as you stated back at the beginning of the results? Everything through Figure 3 (1960-2017) and Figure 4 is really all about past (1988-1995 and 2010-2017). You may want to consider simply using 'Past uses vs. Future projected needs'. It looks to me like you are trying to adhere too much to the 'historical, current, future' framing, but didn't say anything about current. I don't think you need to either.

200 – It takes me awhile to figure out that the entire purpose of this paragraph is to explain when the virtual water imports will reach 61.5 km3. You should state this at the beginning of the paragraph rather than near the end. You are not projecting demand here; you are seeking the date when the total imported water exceeds what the Nile can provide, thereby making imports more important than the Nile for Egypt. This should be clearer. One simple way is to put in a paragraph break in line 206. "In keeping with our….", which starts a new paragraph.

204 – Economic population scenarios? What are these?

211 – "… in this decade of the 2020 and most likely in the coming few years" is strangely redundant.

214 – What are scenarios here? I think you mean "…in most population and economic growth scenarios."

Figure 4. Looks like you can combine some footnotes that are identical.

Figure 6. "Grey shading represents the additional growth in a 0% GDP growth, …" is the first growth meaning the growth in water demand/virtual water trade? It's confusing for the reader.

256 – watch the term "control population growth". Perhaps "manage population growth" is more politically correct.

271 – "This effort was focused on land productivity…." Was the effort increasing the productivity with fertilizer, or increasing the land under cultivation? "… a resource that is much less constrained than water." Don't you need both?

267 – Reuse from groundwater? I can see reuse of wastewater and agriculture drainage that might have originated as groundwater, but what does it mean to re-use groundwater?

315 – “Future import policies need to ensure that Egypt is not totally dependent on one import or export crop, and not dependent on one country for buying or selling goods.” This seems obvious and somewhat hyperbolic. I don’t thing Egypt has ever pondered only one import or export crop, nor has only one country for buying and selling goods. I don’t know how many crops or countries are at play in reality, but you need to be accurate with wording.

329 – If you are going to recommend increasing imports of virtual water from Sudan in the form of meat, you need to mention that it might require additional irrigation water drawn from the Nile, which is exactly what Egypt does not want to happen. The risk needs to be mentioned. Rainfed grass for cattle perhaps?

345 – “decreased human fertility RATES,” I’m sure the fertility of most Egyptians is doing just fine.

363 – If you mention the GERD, you should remind readers here that it is a non-consumptive project, and the risk of increased water stress would be the result of increases in water usage in Sudan because of the GERD.
https://www.sciencedirect.com/science/article/abs/pii/S004896971830648X

Reviewer #2 (Remarks to the Author):

Overall, very interesting analysis and overview of Egypt's past, present, and future water needs. Most of my concerns were addressed in the edits.

The discussion of population control, as a policy intervention, still seems misplaced and poorly integrated into the paper. Listing population control as a main WSA strategy (on par with importing more meat) detracts from the otherwise water-specific focus of the paper. You might instead refer to the need to reduce population growth as a general strategy in the discussion (i.e. water policies will only be effective up to a point - we also need to curb population growth globally, not just in Egypt - especially since Egypt's population is following the same trajectory as world population growth (lines 45-47)).

If the authors do want to advocate for population control as a policy intervention, I recommend including more discussion of the population growth measures recommended by others (rather than citing their paper).

The following is a list of suggested minor edits.

Minor edits:
* Line 82: Capitalize "c" in "Controlling".
* Line 89: Redundant to say "adds additional"...
* Lines 90 - 91: Strengthen by summarizing the findings "we use water data to illustrate an evolving Egypt" -> evolving how?
* Line 91: Correct to "its" challenges.
* Lines 93 - 97: Edit.
* Line 103: Rather than writing "three main periods," which suggest that you're looking at specific periods of time, consider changing to "This paper focuses on past, current, and future trends in Egyptian water management."
* Line 131-33: Population control (as a policy) is still not in the same category as importing meat. While I appreciate that the language has changed from "aggressive population control," it still needs to be explained somewhere what you mean and why these two policies emerge as the two WSA strategies you've decided to highlight. It doesn't fit with the technical (and water) focus of the paper.
* line 241: Consider substituting "application" for "perusal"
* 275 - 276: Worth describing what their suggested policies are in this paper (and what you are proposing).

To the Reviewers,

Thank you again for revisiting our manuscript titled "Will Egypt Continue to be "the Gift of the Nile"?" (NCOMMS-20-38940A) and providing additional comments. We address your concerns in the point-by-point response below and hope that the updates are satisfactory. The changes are also shown in an accompanying tracked-changes document.

Sincerely,
Catherine A. Nikiel and Elfatih A.B. Eltahir

~ ~ ~

REVIEWER 1

"The article has been significantly improved in this revision. The analysis itself shows snapshots in time of Egypt's water use, and this is the biggest contribution of this paper. There are some additional issues that need to be addressed, however. Most are editorial in nature, and few of substance."

Thank you for your review - We have addressed all remaining concerns, following your recommendations closely.

~

C1: "The main concern is messaging of the work. The argument of highlighting the greater dependency of Egypt on the Virtual water compared to the Nile is interesting, but it does not mean that Egypt isn't going to be dependent on the Nile. This should be clear."

A1: We acknowledge that we have not really discussed our framing of "the gift of the Nile" beyond the title and mention in the abstract and so have adding a section discussing this again in the conclusions (Lines 750-759). The paragraph is as follows:

"In the framing of this study, we posed the question of whether Egypt will continue to be the gift of the Nile. Historically and culturally the two are synonymous, and Egypt was, is, and will continue to be dependent on the resources the Nile provides. However, historical reconstruction and future projections show that the level of dependance has been and must continue to change. In the near future, Egypt will be dependent on external virtual water to the same level as its level of dependence on the River, and policies and attitudes will need to reflect and

adapt to this new reality. Through the reconstruction of Egypt’s water demand we have shown here that Egypt is approaching a threshold between the Nile as a dominant force in sustaining Egypt’s growth and existence, and a new paradigm characterized by an equally important role for basin and global interconnection and cooperation.”

We modify the last paragraph of the introduction to achieve the same objective of message clarity:

“In this work we identify and quantify actions that Egypt has taken over the past six decades to manage internal pressures on water resources. A detailed, long term picture of the changes in water demand and water use is constructed and used as a foundation to project demand on water in the near future, and further to propose solutions that can be explored towards more efficient water use. While much past work [22] [23] [24] [25], including governmental literature, has presented snapshots in time of water use and virtual water trade in Egypt, we use water and crop data to quantitatively describe in significant detail water use in Egypt, over a period of six decades. The key innovations of our study are in the detailed year-by-year reconstruction of trends in water use down to the individual crop level, the improved understanding of the factors that drive these trends, and the use of this context to project water demand into the near future based on empirical demand relationships. The detailed diagnosis of water use in Egypt facilitates identification of opportunities for water saving, water reuse, and improved water use efficiency in general.”

~

C2: “The second thing I struggled with are the 'recommendations', which are not really part of the analysis done. WVA and WSA are proposed solutions, but not described in this paper, leaving some of the recommendations somewhat normative. Are there any references that the reader can go to learn more about these methods? If the authors really want to promote these as solutions, then a more explicit description should be provided in the supplementary materials. It would be preferable however to have these methods published elsewhere before introducing them here superficially. I recognize that adding any kind of an in-depth analysis here of solutions would greatly exceed the permitted length of the article.”

A2: We have come to agree with the recommendation to remove WSA and WVA completely as proposed policy umbrellas in the paper. The policy ideas that they contained are only broached in the discussion but not discussed in depth in the paper, and we do not analyze their potential impact in the future. The paragraph below shows this in the discussion (Lines 563-576):

"Egypt's responses to increasing water demand in the past have demonstrated the severity of the water scarcity situation, and historical adaptations to rising demand on water will need to continue and strengthen. Efforts targeting an increase in productivity of agriculture should pivot to recognizing and leveraging the true value of water as a limited resource through a robust water pricing system [37]. As virtual water imports increase, smart management of agricultural export and import portfolios can leverage Egypt's high agricultural yields and amplify their share of natural water resources through the export of high value, high water efficiency crops (fruits and vegetables) and the import of low value, low water efficiency crops (grains). Inter-basin connectivity will be key in the future and using these relationships to import water-intensive commodities like meat can allow allocation of water elsewhere [38]. Finally, studies have shown that a high rate of population growth is one of the most important factors in worsening future water deficits [35], and further reducing rates of growth through proven methods like healthcare expansion and education [34] will slow decreases in per capita water share, a key metric of water scarcity. No single solution will be able to compensate for all additional needs."

We move the discussion of the policy proposals to a new Supplementary Note 2. Additionally, we pull the original Supplementary Note 1 from the Supplementary Materials into the main paper, as it relates to Figure 2 and gives more information on the historical changes in Egypt over the last six decades (See heading "Egypt's Response to Increasing Water Demands" in the Results; Starting Line 372)

~

C3: "The third issue is the values used for inflows into Lake Nasser. These has changed since the previous draft.

In the previous version:
1988-1995 = 71.4 km3; 2010-2017 = 73.1 km3
In the revised version:
1988-1995 = 70.5 km3; 2010-2017 = 77.1 km3

Existing data sources indicate:
1988-1995 = 70.2 km3; 2010-2017 = 72.4 km3

These differences must be reconciled before this can be published. The authors need to confidentially provide the original data for Dongola flows used in this paper for a comparative review with other reliable sources."

A3: we note again that the inflow data used in the revised version is the most up to date and accurate data available from the Ministry of Irrigation and Water Resources, Republic of Sudan. The data was compiled and validated by a Senior official at the Ministry, Mr. Abdelrahman Saghayroon (a.saghayroon@gmail.com). The updated data corrects a previous version of inflow data from the Ministry where processing errors for several years were fixed, with the average annual error between the two versions being 1 km^3 (1961-2017 average of differences) but having significant errors in three years. We have provided this data (Monthly flows from 1890-2020) in an EXCEL file called "MIWR_DongolaFlow" along with this response and revision. We would again like to emphasize that this update increases the accuracy of our results, while not changing the main conclusions.

~

C4: "Note your framing is being used, but it's still a good one. "9.6.7 Can the Nile Take Its 'Gift' Back?" in https://link.springer.com/chapter/10.1007/978-3-030-03698-0_9"

A4: We have looked at the citation provided and acknowledge that the natural use of the framing in different studies make it a relatable entry point for readers. Please see C/A1 for further discussion of the framing and an additional paragraph in the conclusion.

~

C5: Line 10-12 - Eliminate the first sentence of the abstract. It doesn't add value.

A5: We have removed this sentence from the abstract but retained it in the introduction as it provides a historical context for the work.

~

C6: Line 13 - Prospers? That is debatable, depending on your frame of reference. Use another word?

A6: We have replaced this with "expanded" (Line 10)

~

C7: Line 14 - Carry? That is a strange word to use.

A7: We have replaced this with "present" (Line 10)

~

C8: Line 14 - Best to specify "on how water demand for water in Egypt outstripped…."

A8: We have modified this statement as follows (Lines 10-13):

"Here, we present a comprehensive analysis to reconstruct how total demand on water outstripped supply of the Nile water in the late 1970s, starting from a surplus of about 20 km^3 per year in the 1960s leading to a deficit of about 40 km^3 per year by the late 2010s."

~

C9: Line 15-16 - Strange to put what is well documented in the abstract. Words are precious. Use them succinctly and powerfully here

A9: We clarify this statement to reflect that these things are documented in this study, by us. "The role of economic growth in driving per capita demand on water is quantified based on detailed analysis of water use by agriculture and other sectors." (Lines 13-15)

~

C10: Line 17 - "engages the drivers…" - strange wording

A10: Reworded as "We develop and test an empirical model of water demand in Egypt that relates demand on water to growth rates in the economy and population". (Lines 15-16)

~

C11: Line 18 - "project show"?

A11: Update to be "project" (Line 17)

~

C12: Line 31 - Avoid insinuating 'pressures on water rights' when that is under the topic under debate. 'perceived water rights' is a better way to go.

A12: Added "perceived" to this statement. (Line 97)

~

C13: Line 34 - "Current policies on irrigation…" You need a citation here. Watch your wording on "population control". That can get backlash. 'Family planning?' Use the most politically acceptable term that is used in Egypt.

A13: We adjust this phrasing to be "The current policies regarding irrigation in the New Lands, the current rate of water reuse, and the level of success achieved in reducing fertility rates will not be enough to close the demand gap in the future [2]."

We adjust "population control" to "success in reducing fertility rates" but have also moved to deemphasize population as a policy component. Please see C/A47 on further adjustments to our discussion of future population measures in response to concerns from Reviewer 2 on the topic.

~

C14: Line 35 - Citation 2 is the 1959 Treaty, not on the GERD. Use something recent. Also, you might want to mention that the impacts from the GERD on Egypt are under extensive debate.

A14: We eliminate this statement entirely and leave only the mention of the GERD in the conclusion, with the note that it is a subject of debate, with the potential for increased upstream withdrawals.

~

C15: Line 37 - The first sentence is strange. How does Egypt's long existence actually give us insight on current water

pressures? Are you talking in a relative sense? Nothing it the paragraph helps make that connection, other than stating the obvious. More people, more demand. That is not 'insight'. This paragraph seems strangely incomplete.

A15: We have removed the first sentence of this paragraph and expanded on this paragraph. See Lines 103-110 below:

"Egypt's population has been growing rapidly in recent decades, at a rate of 2.1% annually from 1989 to 2018 [3], following a similar trajectory of world population growth (Figure 1a). This added population places pressure on limited water resources, both through direct consumption and through increased demand for food and other products. In 2017, the total renewable water resource per capita was 628 m3/yr already below the level for water scarcity according to the Falkenmark Index [4] [5]. This pressure due to population growth, while straightforward, is essential to include while drawing the picture of historical and future demand for water, as Egypt faces increasing scarcity of natural resources."

~

C16: Line 52 – "From Sudan's withdrawal of 4 km3 in 1959". A current estimated use in Sudan is 16.7 bcm, which does not include evaporation losses (cite https://www.nature.com/articles/s41467-020-19089-x). I understand you may want to avoid this difficult issue, but there is no need to state the withdrawal in 1959. This must reflect the latest research.

A16: Although we discuss it later, we have added the current withdrawals to this statement in order to emphasize the change in withdrawal by Sudan that has occurred throughout the analysis period. "…as a result of increased withdrawal of natural flows upstream from Sudan's withdrawal of 4 km3 in 1959 to current withdrawals of 13-16.7 km3 [16] [17] [18] [19] [20] [21]." (Lines 190-198)

This range is updated with the new reference:

Wheeler, K.G., Jeuland, M., Hall, J.W. *et al.* Understanding and managing new risks on the Nile with the Grand Ethiopian Renaissance Dam. *Nat Commun* **11,** 5222 (2020). https://doi.org/10.1038/s41467-020-19089-x

~

C17: Line 54 - You've gone from a brief mention of demand, to discussion supplies, and now back to the drivers of demand. Seems odd. This long paragraph is all about food and the evolving diet of Egypt. All good information, but it needs to keep relating back to water. I suggest reversing this paragraph with the previous one, so you talk all about demands and the drivers, then about the supplies.

A17: Agreed, we have reversed the order of the second and third paragraphs, so discussion of demand leads into discussion of supply.

~

C18: Figure 1a/b. Would be great to add when Herotodus said the quote on the plots, but I believe it was ~400 BC. So that might distort things too much.

A18: Yes, he wrote his histories in the 5th century B.C. so we cannot easily put them on the figures.

~

C19: Line Figure 1b - Is there any basis by which these two y axes and scales were aligned? Could be misleading. Please explain!

A19: We have edited the caption for this figure to explicitly tell the reader that there is not a 1:1 correlation between the axes, that the black line belongs to the left axis while the red and blue belong to the right axis, and that we have chosen to present the two separate sets of data on the same plot to reflect that there has been little change in the Nile based on geologic survey [15].

~

C20: Line Figure 1d - What is the basis for the relative increase? Is it the 1960 single point value? An average over a period? This needs to be clear.

A20: The increase is relative to the 1961 value which we provide in the figure caption for 1d.

~

C21: Line 84 – "This response is discussed…" I'm not sure if you are talking about one of these 5 (these) or just the last one.

A21: We are referring to all five of the responses, however, we have eliminated this statement as we have brought the discussion of the five responses into the main paper (See heading "Egypt's Response to Increasing Water Demands" in the Results; Beginning line 372)

~

C22: Line 87 – This paragraph on positioning your paper amongst existing literature is awkward. It oscillates between stating what it does in addition to existing work but doesn't really spell out much of what that is, except for citation 16 (Abdeldakir et al 2018). Nothing else in the history of Egypt? Government planning documents?
Egypt MWRI. (2005). Water for the Future: National Water Resources Plan for Egypt - 2017. Retrieved from http://extwprlegs1.fao.org/docs/pdf/egy147082.pdf, Cairo:
NWRP. (2017). The national water resources plan NWRP 2017-2030-2037 Egypt. Cairo: The Ministry of Irrigation and Water Management.
I'm not disagreeing with the value, but the paragraph doesn't make a very convincing argument.

A22: We have rearranged and adjusted this paragraph to hopefully clarify our purpose in including it – a summarization of the value of our study in the context of existing literature. Please see the adjusted paragraph below, or in Lines 216-228:

"In this work we identify and quantify actions that Egypt has taken over the past six decades to manage internal pressures on water resources. A detailed, long term picture of the changes in water demand and water use is constructed and used as a foundation to project demand on water in the near future, and further to propose solutions that can be explored towards more efficient water use. While much past work [22] [23] [24] [25], including governmental literature, has presented snapshots in time of water use and virtual water trade in Egypt, we use water and crop data to quantitatively describe in significant detail water use in Egypt, over a period of six decades. The key innovations of our study are in the detailed year-by-year reconstruction of trends in water use down to the individual crop level, the improved understanding of the factors that drive

these trends, and the use of this context to project water demand into the near future based on empirical demand relationships. The detailed diagnosis of water use in Egypt facilitates identification of opportunities for water saving, water reuse, and improved water use efficiency in general."

~

C23: Line 115 - Seems like you should mention the 3 periods of analysis, past, present and future, in the introduction.

A23: We have consolidated these periods into historical and future, in order to be consistent in the way that we have split our analysis throughout. This also aligns with the past/future framing we have been using in the previous sections. We have changed the section heading to "Water in Egypt: Historical & Future" (Line 328)

~

C24: Line 116-120 - This list of 5 items was just described in the introduction (lines 81-85). Seems a bit strange.

A24: Agreed that this is repetitive. We eliminate the earlier section and keep the later introduction to the five avenues of historical adaptation in the results. (Lines 330-336)

~

C25: Line 126 - "Reach an import"… ? Grammar.

A25: We have rephrased this into "… we project that Egypt will import 61.5 km^3/yr during this decade of the 2020's." (Lines 343-344)

~

C26: Line 141 - Terms like "Population control" may raise concerns.

A26: Eliminated this statement.

~

C27: Figure 3 - The "dark grey shading" needs to be more visible and explained better. Why is there a range? In 1960, the red

line (total Nile Water Use) is the same as the Virtual water import? Is this a coincidence? Is the top of the grey shading the total demand for water from the Nile and from Virtual water imports?

A27: The shaded areas demonstrate additional demand, and we have edited the caption of Figure 3b to clarify this. The red shading shows the amount of withdrawal that would be necessary without the reuse of water. Similarly, the grey shading shows the additional demand that is satisfied through imports. In other words, if Egypt were to not reuse water, and produce all goods currently imported in country, then the 2017 total Nile Use would be ~130 km^3 as opposed to the current value of 61.5 km^3. The Virtual water amount was very small in 1961 and therefore the shaded portion is much thinner than in the later period.

~

C28: Line 158 – Make sure the reader understand this is the first conclusion.

A28: We add “First” to the paragraph starting on Line 443 to indicate that this is the first conclusion, and that the paragraph starting Line 449 holds the second conclusion --that Egypt direct consumption of the Nile is 61.5 km^3/yr.

~

C29: Line 167 – How do these numbers align with Egypt’s own mass balance estimates? (For example, Egypt estimates their outflows the sea around 12.5 bcm.)
Molle, F. (2019). Egypt. In F. Molle, C. Sanchis-Ibor, & L. Avellà-Reus (Eds.), Irrigation in the Mediterranean: Technologies, Institutions and Policies (pp. 243-277). Cham: Springer International Publishing.
NWRP. (2017). The national water resources plan NWRP 2017-2030-2037 Egypt. Cairo: The Ministry of Irrigation and Water Management.

A29: We define the direct discharge to the Mediterranean as the “surplus of Nile freshwater reaching the Mediterranean” in the reaches, given as 2.5-4.0 in the reference below [32]. However, this does not include the addition of outflow through drains (listed as a major outflow source in the national water resources plan (NWRP, 2017) Table 4-11. This outflow is still consistent with our estimates, as boxes marked as “Losses”

(either Evaporative or Drainage) in Figure 4 amount to a total of 23.2 km^3 in 1988-1995 and 19.2 km^3 in 2010-2017.

[32] Hamza, W. The Nile Estuary. In P. Wangersky (ed), Estuaries; Handbook of Environmental Chemistry. Vol. 5 Part H, pp. 149-173 (Springer Verlag, Heidelberg, 2006)

~

C30: Line 169 – It is suggested to not try to quantify how much of Sudan's share is 'underutilized' since there is significant debate on that issue with respect to accounting for evaporation losses.

A30: We amend this to "unconsumed" as we are pointing to the difference between the 1959 allocation of 18.5 km^3 and the estimated consumption of 13-16.7 km^3 per year. We do update this unconsumed range to 1.8-5.5 to better align with these numbers. (Line 454)

~

C31: Line 172 – Include up to 16.7 bcm.

A31: We update our range for Sudanese withdrawals to 13 – 16.7 and add the reference below here and where we briefly acknowledge the potential impacts of the GERD on upstream withdrawals.

Wheeler, K.G., Jeuland, M., Hall, J.W. *et al.* Understanding and managing new risks on the Nile with the Grand Ethiopian Renaissance Dam. *Nat Commun* **11,** 5222 (2020). https://doi.org/10.1038/s41467-020-19089-x

~

C32: Line 177 – Figure 3 - No need to list all the fruit and vegetables independently here. I imagine this could be in an appendix or methods somewhere. Does Figure 3a 'application' include water from the Nile + re-use? I assume the Total Nile Water Use line in Figure 3b is derived using the same information on Figure 3a, but with municipal and industrial added, and reuse subtracted. By that logic, I guess this is why the red line in Figure 3b starts higher, but ends lower than the sum of the applications in Figure 3a. I guess this begs the question then why is the top of the red band in Figure 3b over

80 bcm in 2017, but the total water applied to agriculture is around 70 bcm in 2017 (Figure 3a).

A32: We have eliminated the listing of fruits and vegetables in the caption and made sure that this grouping is still defined in the methods. Please see C/A27 that clarifies the shadings as additional demand captured in those categories, rather than ranges of values.

~

C33: Line 198 – What happened to the "current" section as you stated back at the beginning of the results? Everything through Figure 3 (1960-2017) and Figure 4 is really all about past (1988-1995 and 2010-2017). You may want to consider simply using 'Past uses vs. Future projected needs'. It looks to me like you are trying to adhere too much to the 'historical, current, future' framing, but didn't say anything about current. I don't think you need to either.

A33: Please refer to C/A23. We have edited the framework to be historical & future only, eliminating current.

~

C34: Line 200 – It takes me awhile to figure out that the entire purpose of this paragraph is to explain when the virtual water imports will reach 61.5 km3. You should state this at the beginning of the paragraph rather than near the end. You are not projecting demand here; you are seeking the date when the total imported water exceeds what the Nile can provide, thereby making imports more important than the Nile for Egypt. This should be clearer. One simple way is to put in a paragraph break in line 206. "In keeping with our….", which starts a new paragraph.

A34: We make the paragraph break suggested, separating the discussion of how we project demand, from the ultimate purpose of that projection as stated above. (Line 520)

~

C35: Line 204 – Economic population scenarios? What are these?

A35: Typing error, this should be "economic and population scenarios" and can be seen separately in Supplementary Figure 11. (Line 516)

~

C36: Line 211 – "… in this decade of the 2020 and most likely in the coming few years" is strangely redundant.

A36: We have shortened this statement to "this important benchmark will be reached in this decade of the 2020's." (Line 525)

~

C37: Line 214 – What are scenarios here? I think you mean "…in most population and economic growth scenarios."

A37: Agreed, we have edited this statement to read "in most population and economic growth scenarios" (Line 529)

~

C38: Figure 4. Looks like you can combine some footnotes that are identical.

A38: Footnotes have been consolidated and superscripts have been updated accordingly.

~

C39: Figure 6. "Grey shading represents the additional growth in a 0% GDP growth, …" is the first growth meaning the growth in water demand/virtual water trade? It's confusing for the reader.

A39: Agreed that this is a confusing statement, it has been edited to "Grey shading represents the additional virtual water imports in a 0% GDP growth …" (Figure 6 caption; Lines 551)

~

C40: Line 256 – watch the term "control population growth". Perhaps "manage population growth" is more politically correct.

A40: We have changed this phrasing to "reducing population growth rate" (Line 334)

~

C41: Line 271 – "This effort was focused on land productivity…."

Was the effort increasing the productivity with fertilizer, or increasing the land under cultivation? "… a resource that is much less constrained than water." Don't you need both?

A41: Increasing the area under cultivation would increase the total production, but we focus in this section on measures that increase the yield (i.e. tonnes/unit area cultivated) through management strategies (i.e. fertilizer application).

~

C42: Line 267 – Reuse from groundwater? I can see reuse of wastewater and agriculture drainage that might have originated as groundwater, but what does it mean to re-use groundwater?

A42: The groundwater component here is considered shallow groundwater from the Nile Valley and Delta. We count this as reuse because often this shallow groundwater comes from over-application at the field scale. Other sources also categorize this groundwater as a secondary use (reuse). See Table 3.1 in the reference below, which is modified from Egypt's Vision 2030 Sustainable Development Strategy

[22] Abu Zeid, K. M. Existing and Recommended Water Policies in Egypt. *Water Policies in MENA Countries*, Global Issues in Water Policy 23, S. Zekri (ed.) (2020) https://doi.org/10.1007/978-3-030-29274-4_3

~

C43: Line 315 – "Future import policies need to ensure that Egypt is not totally dependent on one import or export crop, and not dependent on one country for buying or selling goods." This seems obvious and somewhat hyperbolic. I don't thing Egypt has ever pondered only one import or export crop, nor has only one country for buying and selling goods. I don't know how many crops or countries are at play in reality, but you need to be accurate with wording.

A43: We say this statement on the basis of the next section: "This is especially important in light of difficulties imposed by the COVID-19 pandemic. Currently 69% of Egypt's main import, wheat, comes from Russia, and roughly 90% from Russia and former Soviet Republics [16]. As exporters of staple products, such as Russia, cut back in light of their own production concerns during the coronavirus pandemic [17], Egypt needs to re-examine their relationships and dependencies." In order for this to be

clear we move the quoted statement to after this section. Also note that this entire paragraph and all discussion of policy proposals is moved to the Supplementary Material (Supplementary Note 2)

~

C44: Line 329 – If you are going to recommend increasing imports of virtual water from Sudan in the form of meat, you need to mention that it might require additional irrigation water drawn from the Nile, which is exactly what Egypt does not want to happen. The risk needs to be mentioned. Rainfed grass for cattle perhaps?

A44: This section has been moved to the supplementary material (Note 2) along with discussion of the other policy proposals. However, we do add the following sentence and reference "Sudan currently raises livestock almost entirely under rain-fed, nomadic conditions although this could change with future irrigation development."

Food and Agriculture Organization of the United Nations (FAO). AQUASTAT Country Profile – Sudan. (Rome, Italy, 2016). Available Online at http://www.fao.org/3/i9808en/I9808EN.pdf

~

C45: Line 345 – "decreased human fertility RATES," I'm sure the fertility of most Egyptians is doing just fine.

A45: Agreed and updated.

~

C46: Line 363 – If you mention the GERD, you should remind readers here that it is a non-consumptive project, and the risk of increased water stress would be the result of increases in water usage in Sudan because of the GERD. https://www.sciencedirect.com/science/article/abs/pii/S004896971830648X

A46: We modify this statement to "…but is acknowledged as an additional potential stressor, especially with regards to increased upstream withdrawals [21] [39]." (Lines 583) And added the following references:

[44] Basheer, M., Wheeler, K. G., Ribbe, L., Majdalawi, M., Abdo G. & Zagona, E. A., Quantifying and evaluating the impacts of cooperation in transboundary river basins on the Water-Energy-Food nexus: The Blue Nile Basin, *Science of The Total Environment,* **630**, 1309-1323 (2018) https://doi.org/10.1016/j.scitotenv.2018.02.249.

[21] Wheeler, K.G., Jeuland, M., Hall, J.W. *et al.* Understanding and managing new risks on the Nile with the Grand Ethiopian Renaissance Dam. *Nat Commun* **11,** 5222 (2020). https://doi.org/10.1038/s41467-020-19089-x

REVIEWER 2:

"Overall, very interesting analysis and overview of Egypt's past, present, and future water needs. Most of my concerns were addressed in the edits."

Thank you for your review, and we hope that we have addressed your remaining concerns. The comments about population were given special care.

~

C47: The discussion of population control, as a policy intervention, still seems misplaced and poorly integrated into the paper. Listing population control as a main WSA strategy (on par with importing more meat) detracts from the otherwise water-specific focus of the paper. You might instead refer to the need to reduce population growth as a general strategy in the discussion (i.e. water policies will only be effective up to a point - we also need to curb population growth globally, not just in Egypt - especially since Egypt's population is following the same trajectory as world population growth (lines 45-47)).

If the authors do want to advocate for population control as a policy intervention, I recommend including more discussion of the population growth measures recommended by others (rather than citing their paper).

A47: To start in addressing your concerns, we have removed the use of WSA and WVA as policy umbrellas from the paper, and therefore no longer unintentionally equate population measures

and importing meat as policy areas. Since population is woven through the paper as both a strong driver of historical demand, and as an area where Egypt has made big changes in the past, we leave these mentions (making sure to moderate the language) and only note population growth reduction as a key consideration, and one that can be achieved through further education and healthcare expansion. We have also eliminated the section comparing Egypt's population with that of Asia, to avoid delving into more controversial areas that steer away discussion from the pressure that population growth places on water scarcity.

"Finally, studies have shown that a high rate of population growth is one of the most important factors in worsening future water deficits [35], and further reducing rates of growth through proven methods like healthcare expansion and education [34] will slow decreases in per capita water share, a key metric of water scarcity."

~

C48: Line 82: Capitalize "c" in "Controlling".

A48: This section was redundant based on a later section and so was removed. Please refer to C/A24.

~

C49: Line 89: Redundant to say "adds additional"...

A49: This sections was removed.

~

C50: Lines 90 - 91: Strengthen by summarizing the findings "we use water data to illustrate an evolving Egypt" -> evolving how?

A50: We update this statement as "While much past work [22] [23] [24] [25], including governmental literature, has presented snapshots in time of water use and virtual water trade in Egypt, we use water and crop data to quantitatively describe in significant detail water use in Egypt, over a period of six decades." (Lines 220-223)

~

C51: Line 91: Correct to "its" challenges.

A51: Corrected.

~

C52: Lines 93 - 97: Edit.

A52: We have rewritten this section as follows:

“The key innovations of our study are in the detailed year-by-year reconstruction of trends in water use down to the individual crop level, the improved understanding of the factors that drive these trends, and the use of this context to project water demand into the near future based on empirical demand relationships. The detailed diagnosis of water use in Egypt facilitates identification of opportunities for water saving, water reuse, and improved water use efficiency in general.” (Lines 223-228)

~

C53: Line 103: Rather than writing "three main periods," which suggest that you're looking at specific periods of time, consider changing to "This paper focuses on past, current, and future trends in Egyptian water management."

A53: We have adjusted this opener to “This paper focuses on historical and future trends in Egyptian water management” (Line 330) We have also reframed the separation of the discussion and analysis into a historical and a future period and have eliminated the separation of the present.

~

C54: Lines 131-33: Population control (as a policy) is still not in the same category as importing meat. While I appreciate that the language has changed from "aggressive population control," it still needs to be explained somewhere what you mean and why these two policies emerge as the two WSA strategies you've decided to highlight. It doesn't fit with the technical (and water) focus of the paper.

A54: We understand the concern and have worked to remedy the unintentional pairing/equating of these two policies. Please see C/A47 for further information on the changes made.

~

C55: Line 241: Consider substituting "application" for "perusal"

A55: Removed this section.

~

C56: Lines 275 - 276: Worth describing what their suggested policies are in this paper (and what you are proposing).

A56: The paper cited (Abdelkader et al., 2018) finds that their "sensitivity analysis revealed that the exceptionally high population growth rate in Egypt plays a critical role in pushing the national water and food gaps to alarming levels." But they propose few concrete strategies beyond advocating "that for solving Egypt's water and food problem non-water-based solutions like educational, health, and awareness programs aimed at lowering population growth will be an essential addition to the traditional water resources development solution." We have therefore added the statement quoted in C/A47.

REVIEWERS' COMMENTS

Reviewer #1 (Remarks to the Author):

The authors have done a good job refining the paper during this round of edits. The arguments are now better organized and well-founded. I appreciate the responses to each point raised throughout the review process.

Kevin Wheeler, PhD. P.E.
